# A reversible SRC-relayed COX2 inflammatory program drives resistance to BRAF and EGFR inhibition in BRAF^V600E^ colorectal tumors

**DOI:** 10.1038/s43018-022-00508-5

**Published:** 2023-02-09

**Authors:** Ana Ruiz-Saenz, Chloe E. Atreya, Changjun Wang, Bo Pan, Courtney A. Dreyer, Diede Brunen, Anirudh Prahallad, Denise P. Muñoz, Dana J. Ramms, Valeria Burghi, Danislav S. Spassov, Eleanor Fewings, Yeonjoo C. Hwang, Cynthia Cowdrey, Christina Moelders, Cecilia Schwarzer, Denise M. Wolf, Byron Hann, Scott R. VandenBerg, Kevan Shokat, Mark M. Moasser, René Bernards, J. Silvio Gutkind, Laura J. van ‘t Veer, Jean-Philippe Coppé

**Affiliations:** 1 Helen Diller Family Comprehensive Cancer Center, University of California, San Francisco, San Francisco, CA USA; 2 Departments of Cell Biology & Medical Oncology, Erasmus University Medical Center Rotterdam, Rotterdam, The Netherlands; 3 Division of Molecular Carcinogenesis and Oncode Institute, The Netherlands Cancer Institute, Amsterdam, The Netherlands; 4 Department of Pharmacology, University of California, San Diego, La Jolla, CA USA; 5 Moores Cancer Center, University of California, San Diego, La Jolla, CA USA; 6 Present Address: Department of Breast Surgery, Peking Union Medical College Hospital, Peking Union Medical College and Chinese Academy of Medical Sciences, Beijing, China; 7 Present Address: Faculty of Pharmacy, Medical University of Sofia, Sofia, Bulgaria; 8 Present Address: Institute for Computational Biomedicine, Heidelberg, Germany

**Keywords:** Cancer, Colon cancer, Proteomics, Cancer therapeutic resistance, Cancer therapy

## Abstract

*BRAF*^V600E^ mutation confers a poor prognosis in metastatic colorectal cancer (CRC) despite combinatorial targeted therapies based on the latest understanding of signaling circuitry. To identify parallel resistance mechanisms induced by BRAF–MEK–EGFR co-targeting, we used a high-throughput kinase activity mapping platform. Here we show that SRC kinases are systematically activated in BRAF^V600E^ CRC following targeted inhibition of BRAF ± EGFR and that coordinated targeting of SRC with BRAF ± EGFR increases treatment efficacy in vitro and in vivo. SRC drives resistance to BRAF ± EGFR targeted therapy independently of ERK signaling by inducing transcriptional reprogramming through β-catenin (CTNNB1). The EGFR-independent compensatory activation of SRC kinases is mediated by an autocrine prostaglandin E_2_ loop that can be blocked with cyclooxygenase-2 (COX2) inhibitors. Co-targeting of COX2 with BRAF + EGFR promotes durable suppression of tumor growth in patient-derived tumor xenograft models. COX2 inhibition represents a drug-repurposing strategy to overcome therapeutic resistance in BRAF^V600E^ CRC.

## Abstract (web summary)

Coppé and colleagues define a resistance mechanism to combination BRAF- and EGFR-targeted therapy in BRAF^V600E^ colorectal cancers that could be overcome in vitro and in vivo by repurposing COX2 inhibitors.

## Main

Presence of a *BRAF*^V600E^ kinase mutation predicts the form of metastatic colorectal cancer (mCRC) with the worst prognosis. About 8% of mCRCs harbor a *BRAF*^V600E^ mutation. Because mCRC is the second leading cause of cancer death, it is estimated that more patients die of BRAF^V600E^ mCRC than melanoma each year. Unlike melanoma, BRAF^V600E^ mCRC does not respond to BRAF inhibitor monotherapy, and it responds only poorly to conventional chemotherapy^1–3^. Response rates have been increased by combining BRAF inhibitors with MEK inhibitors and/or an anti-EGFR antibody; however, the majority of mCRC tumors still fail to regress and durability of disease control remains a challenge. Clinical outcomes have been remarkably similar with combinations of BRAF inhibitors (vemurafenib, dabrafenib or encorafenib) and/or MEK inhibitors (trametinib or binimetinib) and/or anti-EGFR antibodies (cetuximab or panitumumab), with a median confirmed response rate of ~20%, progression-free survival of ~4 months and overall survival of ~9 months^4–6^. In April 2020, the Food and Drug Administration (FDA) granted approval to the encorafenib plus cetuximab doublet for the treatment of patients with BRAF^V600E^ mCRC, on the basis of comparable outcomes with this doublet versus triplet combinations evaluated in clinical trials.

The objective of targeting BRAF ± MEK ± EGFR is to reinforce inhibition of the main oncogenic driver pathway (BRAF–MEK) while jointly shutting down the activation of a drug resistance mechanism (EGFR)^5,6^. However, the observed ceiling effect with this approach suggests that other prevalent mechanisms (that is, dependencies) must cooperate to circumvent therapeutic effectiveness. The rational next step is to systematically identify orthogonal vulnerabilities, independent of the MAPK pathway, to inform the design of novel combination strategies to address this ongoing unmet medical need.

To test the hypothesis that other parallel pathways act as compensatory mechanisms to drug treatments, and to initiate an expanded search for complementary drug targets, we leveraged a high-throughput kinase activity mapping (HT-KAM) platform. HT-KAM is a functional proteomic screening technology that enables direct measurement of the catalytic activity of many kinases in parallel^7–10^. This systematic process can help identify the most significantly and specifically perturbed kinase hubs, in turn revealing actionable vulnerabilities (kinases or otherwise) that lie within phospho-circuits of cancer cells and tissues. Strategic kinase dependencies with the highest therapeutic potential can then be chosen for further investigation in cell culture and xenograft models. The ultimate goal is to identify rational therapeutic combinations capable of producing greater than incremental improvements in clinical outcomes for patients with BRAF^V600E^ mCRC.

We found that a highly conserved SRC-relayed inflammatory program drives the adaptive response to targeted therapies in BRAF^V600E^ CRC. Specifically, SRC family kinases were activated upon treatment with BRAF ± MEK ± EGFR inhibitors in vitro and in vivo, thus uncovering an EGFR-independent mechanism of resistance. We found that, upon treatment with BRAF ± EGFR targeted therapy, activation of SRC kinases regulated the downstream phosphorylation of β-catenin (CTNNB1), which led to the reprogramming of cells’ transcriptional profiles. Upstream of SRC, we found that SRC kinases were activated by an autocrine prostaglandin E_2_ (PGE2)-regulated GNAS activation loop that cyclooxygenase-2 (COX2) inhibitors interrupted in both cell lines and patient-derived xenograft (PDX) mouse models^4,11^. This SRC-relayed mechanism of therapeutic resistance operated independently of ERK signaling. We showed that supplementing the current standard-of-care combination of the BRAF inhibitor encorafenib plus an anti-EGFR antibody (panitumumab) with the FDA-approved COX2 inhibitor celecoxib significantly and consistently improved tumor growth inhibition. Overall, our study demonstrates that SRC signaling is at the nexus of a cell-autonomous inflammatory program with pro-tumorigenic activities, which explains why BRAF^V600E^ colorectal tumors develop resistance to BRAF/MEK and EGFR inhibitors. Our results suggest a clinically actionable strategy, the addition of celecoxib to targeted therapies, to restore therapeutic response in BRAF^V600E^ CRC. This drug-repurposing approach is cost effective with minimal added toxicity and may be fast-tracked into clinical testing.

## Results

### BRAF/MEK/EGFR inhibition activates SRC in BRAF^V600E^ CRC

**Fig. 1:**
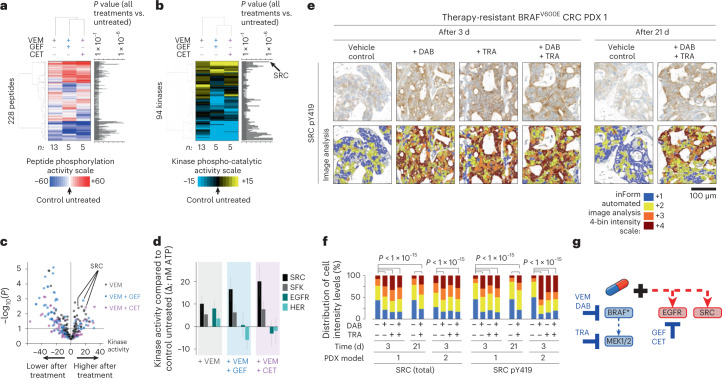
SRC is activated following BRAF/MEK/EGFR inhibition in BRAF^V600E^ CRC. **a**,**b**, Unsupervised hierarchical clustering of the phospho-catalytic activity signatures of WiDr cells treated with vemurafenib (VEM; *n* = 13 independent experiments) ± gefitinib (GEF; *n* = 5 independent experiments) or cetuximab (CET; *n* = 5 independent experiments) as compared to their untreated control counterparts (*n* = 23 independent experiments). **a**, ATP consumption in cell extracts using 228 peptide sensors. **b**, Kinase signatures deconvoluted from the peptide phosphorylation profiles in **a**. Bar graphs next to the heatmaps show the *P* values (two-sided Student’s *t* test) for each of the peptides (**a**) or kinases (**b**) comparing all treated samples to controls. **c**, Volcano plot of the data in **b** displaying the change in kinase activity versus *P* value for each treatment arm (same as **b**: VEM, *n* = 13; VEM + GEF, *n* = 5; VEM + CET, *n* = 5; as compared to their untreated control counterparts (*n* = 23), where *n* is the number of independent experiments). **d**, Bar graphs of the data in **b** representing the shift in activity of SRC, SFK, EGFR and HER family kinases when cells were treated with vemurafenib alone or in combination with gefitinib or cetuximab. Kinase activity is compared to that in untreated control cells, and data are displayed as the average ± standard error in nM of ATP. Same as in **b**,**c**: VEM, *n* = 13; VEM + GEF, *n* = 5; VEM + CET, *n* = 5; as compared to their untreated control counterparts (*n* = 23), where *n* is the number of independent experiments. **e**, Representative IHC images showing staining intensity for active SFK (phosphorylated Y419 epitope in the SRC activation site) following treatment of a BRAF^V600E^ CRC PDX model with vehicle control, dabrafenib (DAB) and/or trametinib (TRA) for 3 or 21 d. The color-coded bottom panel highlights differences in bin intensities from automated image analysis (see Methods for details). IHC images and intensity quantifications are representative of *n* = 2 independent PDX tumors per treatment condition and *n* = 20 independent tissue areas per tumor and per condition. **f**, Quantification of IHC staining intensity for total and activated SFK in two PDX models treated for 3 or 21 d with dabrafenib ± trametinib versus vehicle control (two-sided Student’s *t* test, *P* < 1 × 10^–15^). Using batch processing and automated analysis of IHC images, protein expression was measured at the single-cell level (that is, *n* ≥ 10,000 individual cancer cells per treatment condition and tumor). **g**, Proposed parallel mechanism of SRC activation in response to BRAF/MEK/EGFR therapies in BRAF^V600E^ CRC. BRAF*, BRAF^V600E^. Source data

SRC family kinases were identified as independent functional determinants of the adaptive response to BRAF/MEK/EGFR targeting in BRAF^V600E^ CRC through functional kinome screening. We used the HT-KAM platform to measure kinase activity in extracts processed from WiDr cells, a well-established vemurafenib-resistant BRAF^V600E^ CRC model^12–14^. Cells were treated with a BRAF inhibitor (vemurafenib) ± an EGFR inhibitor (gefitinib or cetuximab) for 8 h. Peptide-level data (Fig. 1a) were then transformed into kinase activity signatures (Fig. 1b) using previously described deconvolution methods^7^. We found that SRC showed the most significantly increased kinase activity in response to BRAF inhibitor-containing treatments (Fig. 1b). Notably, the increase in SRC activity was conserved even following combined treatment with vemurafenib and either EGFR inhibitor (Fig. 1b–d), indicating that SRC is not a surrogate for EGFR-mediated resistance to BRAF-targeted therapies.

**Extended Data Fig. 1:**
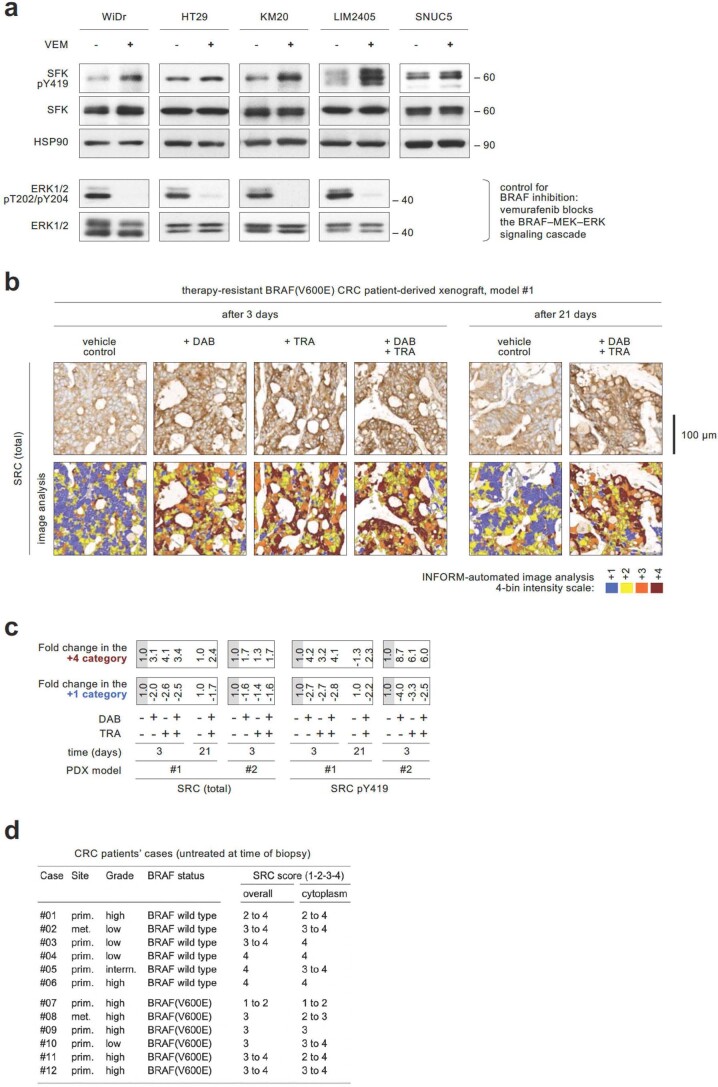
SRC is activated consequent to BRAF/MEK/EGFR inhibition in BRAF^V600E^ CRC specifically. **a**, BRAF^V600E^ CRC cell lines were treated with vemurafenib (VEM) for 7 to 8 hours. Vemurafenib was used at 1.75 uM in HT29, 2 uM in KM20, 0.15 uM in LIM2405, 2.25 uM in SNUC5, and 1.5 uM in WiDr (details of treatment conditions (concentration and time) are available in spreadsheets Supplementary Table 2 and 3 of the Supplementary Tables document). Cell lysates were assayed by western blot with the indicated antibodies. Upper panels: SFK activation is reflected by increased phosphorylation of the SRC activation site, Y419 (pY419). HSP90 is used as loading control. Bottom panel: reduction in ERK 1/2 phosphorylation as control of BRAF inhibition. Molecular weight/size markers are indicated on the right (kDa). The experiment was repeated ≥3 times with similar results. **b**, Representative IHC images showing total SRC staining intensity following treatment of a BRAF^V600E^ CRC PDX model with vehicle control, dabrafenib (DAB) and/or trametinib (TRA) for 3 or 21 days. The color-coded bottom panel highlights differences in bin intensities resulting from automated image analysis (see Methods for details). A scale bar is provided (100 micrometers). As in main Fig. 1e, IHC images and intensity quantifications are representative of n = 2 independent PDX tumors per treatment condition, and n = 20 independent tissue areas per tumor and per condition. **c**, Quantification of IHC staining intensity for total and activated SFK in two PDX models treated for 3 or 21 days with DAB ± TRA vs. vehicle control. As in main Fig. 1f, we used batch processing and automated analysis of IHC images to quantify protein expression at the single cell level (that is, n ≥ 10,000 individual cancer cells per treatment condition and tumor). **d**, SRC staining score by IHC in untreated patient CRC tumor specimens with or without a BRAF^V600E^ mutation, from primary (prim.) or metastatic (met.) sites. Source data

SRC belongs to the SRC family kinase (SFK) proteins, which include 11 membrane-associated, non-receptor tyrosine kinases that regulate cell proliferation, differentiation, apoptosis, migration and metabolism, among other processes^15–18^. We validated the observed kinase activity signature by western blot in a panel of BRAF^V600E^ CRC lines after vemurafenib treatment. Results showed increased SFK activation, as reported by the phosphorylation of Y419 (Extended Data Fig. 1a). We sought further substantiation of SRC-mediated resistance using rare xenograft models of BRAF^V600E^ CRC derived from patients who were subsequently treated with dabrafenib and trametinib on a clinical trial^4^. PDX 1, and the corresponding patient’s tumor biopsy, exhibited primary resistance to treatment with dabrafenib + trametinib, whereas patient/PDX 2 exhibited early tumor regression and eventual progression. Using automated image analysis of immunohistochemistry (IHC) profiles, we observed statistically significant increases in the levels of active and total SRC in both PDX models after 3 or 21 d of treatment with dabrafenib and/or trametinib (Fig. 1e,f and Extended Data Fig. 1b,c). These data place SRC activation as an early adaptive response to BRAF inhibition, which is maintained even in residual tumors following BRAF and/or MEK inhibitor treatment. Unlike EGFR levels, which are upregulated at baseline in BRAF^V600E^ cell lines^12,13^, comparison of untreated primary patient colorectal tumor specimens harboring or not harboring a *BRAF*^V600E^ mutation showed that patient tumors start with similar levels of total SRC (Extended Data Fig. 1d). Together, these findings led us to postulate that SRC is an EGFR-independent candidate drug target to overcome resistance to BRAF/MEK inhibitor therapies (Fig. 1g).

### SRC kinase inhibitors sensitize BRAF^V600E^ CRC to vemurafenib

**Fig. 2:**
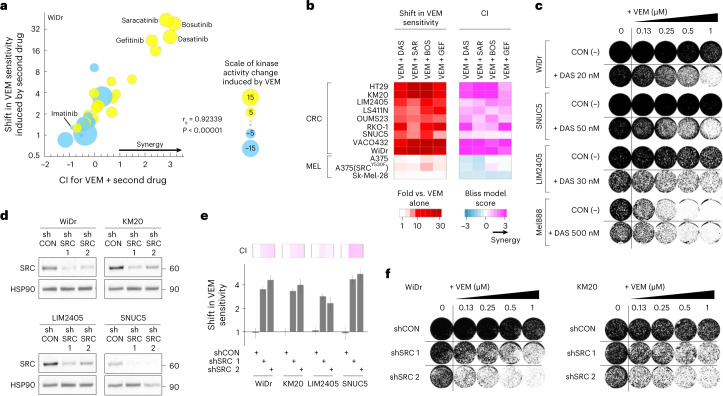
SRC kinase inhibition sensitizes BRAF^V600E^ CRC cell lines to vemurafenib. **a**, Cell viability assays evaluating WiDr cells treated with vemurafenib plus a panel of kinase inhibitors selected on the basis of the results in Fig. 1b. The size of each bubble indicates the magnitude of the change in kinase activity induced by vemurafenib treatment (Fig. 1b), with color signifying increased (yellow) or decreased (blue) activity; (*y* axis, log_2_ scale). *r*_s_ is the Spearman’s rho correlation, and the *P* value is from a two-tailed *t* test. Drug combinations were tested in *n* = 89 independent experiments. **b**, Shift in vemurafenib sensitivity, measured by cell viability assay (left) and calculation of the CI (right), upon treatment of BRAF^V600E^ CRC or melanoma (MEL) cell lines with vemurafenib together with a SRC inhibitor (dasatinib (DAS), saracatinib (SAR) or bosutinib (BOS)) or an EGFR inhibitor (gefitinib) for 3 d. CI scores are averaged from individual experimental CIs calculated at 1×GI_50_, 2×GI_50_ and 0.5×GI_50_ concentrations of each drug (*n* ≥ 3 independent experiments). **c**, Colony formation assays in which BRAF^V600E^ CRC or melanoma (Mel888) cells were treated with an increasing concentration of vemurafenib alone (control, CON) or with a fixed dose of dasatinib. Data are representative of *n* = 2 independent repeats. **d**, Western blots showing knockdown of SRC in BRAF^V600E^ CRC cell lines stably transfected with a control shRNA (shCON) or two different SRC-targeting shRNAs (shSRC). HSP90 serves as a loading control. Molecular weight/size markers are indicated on the right (kDa). The experiment was repeated three independent times with similar results. **e**, Bar graphs representing the fold change (log_2_ scale) ± standard error for change in sensitivity to vemurafenib with knockdown of SRC in 3-day cell viability assays. Top, CI, Bliss model score; colors as in **b** (*n* = 3 independent experiments per cell line). **f**, Colony formation in BRAF^V600E^ CRC cells treated with an increasing concentration of vemurafenib with or without SRC knockdown. Data are representative of *n* = 2 independent repeats. Source data

**Extended Data Fig. 2:**
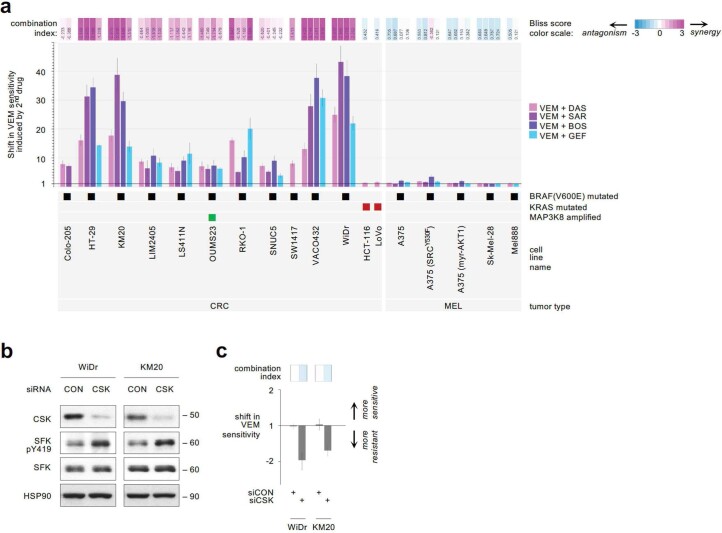
SRC kinase activity is inversely correlated with sensitivity to vemurafenib in BRAF^V600E^ CRC cell lines. **a**, Shift in vemurafenib (VEM) sensitivity (mean fold-change ± standard error) measured via cell viability assays and calculation of the combination index (CI score; top panel) upon treatment of BRAF^V600E^ or KRAS mutated or MAP3K8 amplified CRC, or melanoma (MEL) cell lines with VEM together with: a SRC inhibitor, dasatinib (DAS), saracatinib (SAR) or bosutinib (BOS), or an EGFR inhibitor, gefitinib (GEF), for three days. Same methods as in Fig. 2b (n = 3 independent experiments per cell line and per drug combination). **b**, BRAF^V600E^ CRC cell lines were transfected with a siRNA against CSK, a negative regulator of SFKs, and siRNA Control. Cell lysates were assayed by western blot with the indicated antibodies, showing the effect of CSK depletion on SFK activation. Molecular weight/size markers are indicated on the right (kDa). Experiment repeated 3 independent times with similar results. **c**, Bar graphs representing fold-change (log scale) ± standard error for change in sensitivity to VEM with knockdown of CSK in 3-day cell viability assays. Top panel: combination index, Bliss model score; colors as in Fig. 2b (n = 4 independent experiments). Source data

To test the hypothesis that SRC is a druggable vulnerability in BRAF inhibitor-resistant BRAF^V600E^ CRC, we first assessed the sensitivity of WiDr cells to two-drug combinations including a BRAF inhibitor and another kinase inhibitor, chosen on the basis of the kinase signatures in Fig. 1b. Consistently, the greatest increases in cell growth inhibition and the highest combination index (CI) scores, that is, synergy, were observed when the BRAF inhibitor was combined with a SRC inhibitor: dasatinib, saracatinib or bosutinib (Fig. 2a; all CI > 2.8). The potentiation in sensitivity to vemurafenib with the addition of a SRC inhibitor was conserved across various vemurafenib-resistant BRAF^V600E^ CRC cell lines (HT29, KM20, LIM2405, LS411N, OUMS23, RKO1, SNUC5, VACO432, WiDr), as shown in Fig. 2b; the EGFR inhibitor gefitinib was included for comparison. This observation was specific to BRAF^V600E^ CRC, as it was not recapitulated in vemurafenib-sensitive BRAF^V600E^ melanoma cells (A375, A375 (SRC^Y530F^), A375 (myr-AKT1), Sk-Mel-28, Mel888) or *BRAF*-wild-type CRC cells (HCT116, LoVo), as shown in Fig. 2b and Extended Data Fig. 2a. Furthermore, dual treatment with vemurafenib + dasatinib strongly inhibited colony formation in BRAF^V600E^ CRC cells, but not BRAF^V600E^ melanoma cells, at concentrations where dasatinib alone had a minimal effect (Fig. 2c). To further validate SRC’s role as a mediator of the response to vemurafenib, we knocked down SRC using short hairpin RNA (shRNA) (Fig. 2d) and found that SRC-deficient BRAF^V600E^ CRC cells were more sensitive to vemurafenib treatment in 3-day viability and colony formation assays (Fig. 2e,f), although, as expected, the effect was not as profound as when using SFK inhibitors. Conversely, small interfering RNA (siRNA) knockdown of C-terminal SRC kinase (CSK), a negative regulator of SFK, led to increased SFK activation (Extended Data Fig. 2b) and reduced sensitivity to vemurafenib (Extended Data Fig. 2c). Collectively, these data substantiate our hypothesis that SRC is a promising new target to overcome resistance to BRAF inhibitor therapies in BRAF^V600E^ CRC.

### Targeting of SRC with BRAF + EGFR increases efficacy

**Fig. 3:**
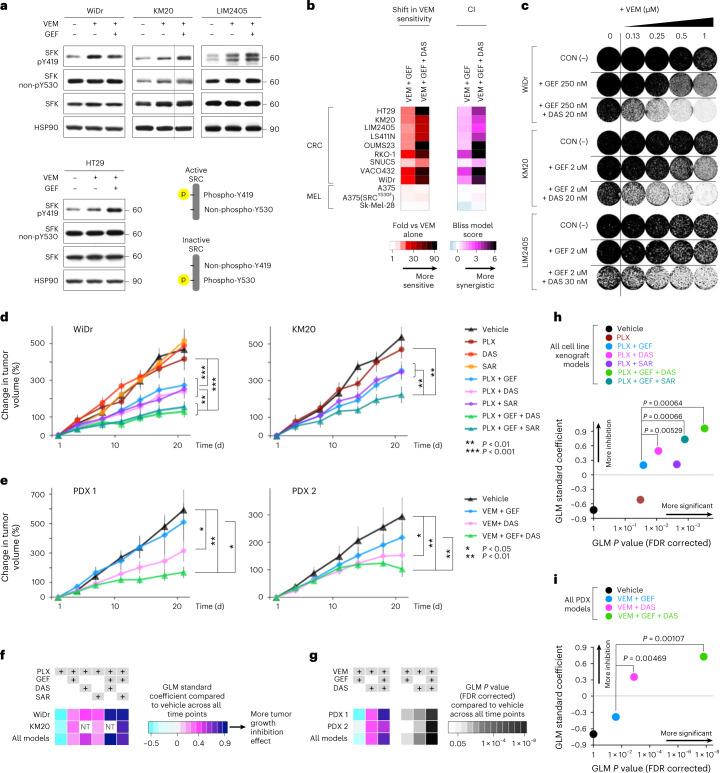
Coordinated targeting of SRC with BRAF + EGFR increases efficacy in BRAF^V600E^ CRC cell lines and xenografts. **a**, BRAF^V600E^ CRC cell lines treated with vemurafenib ± gefitinib were lysed and immunoblotted with the indicated antibodies. SFK activation is reflected by increased phosphorylation of the SRC activation site Y419 (pY419) and lack of phosphorylation of the inhibitory site Y530 (non-pY530). Active SRC can be deactivated by rephosphorylation of Y530 by CSK. HSP90 serves as a loading control. Molecular weight/size markers are indicated on the right (kDa). The experiment was repeated three times with similar results. **b**, Shift in vemurafenib sensitivity measured by cell viability assay (left) and calculation of the CI (right) upon treatment of BRAF^V600E^ CRC or melanoma cell lines with vemurafenib + gefitinib ± a SRC inhibitor, dasatinib, for 3 d (*n* = 4 independent experiments per cell line). **c**, Colony formation assays in which BRAF^V600E^ CRC cells were treated with an increasing concentration of vemurafenib alone (control) or with a fixed dose of gefitinib ± dasatinib. Data are representative of *n* = 2 independent repeats. **d**, Treatment of cell line-derived xenograft mouse models with a vemurafenib progenitor, PLX4720 (PLX); dasatinib; saracatinib; and/or gefitinib for 21 d (*n* = 7 mice per group). Plotted is the percent change in tumor volume relative to baseline (day 1). Data are displayed as the average for all mice in a specified treatment group ± standard error. **e**, Treatment of PDX models with vemurafenib ± gefitinib ± dasatinib for 21 d, with data plotted as in **d** (*n* = 8 mice per group). All raw and relative tumor volumes and exact *P* values shown in **d**,**e** are available as Source Data; *P* values are from a two-sided Student’s *t* test. **f**,**g**, GLMs testing the association of change in tumor volume between treatment arms and vehicle over time shown in **d**,**e**. Effect size is measured as the GLM standard coefficient. A GLM was applied to each tumor model separately or combined. Results for cell line xenografts and PDXs are shown in **f** and **g**, respectively. GLM *P* values corrected for FDR are shown in **g**. NT, not tested. **h**,**i**, Comparison of the effect sizes and FDR-corrected *P* values of treatment arms with and without a SRC inhibitor. The same number of mice per group shown in **d**,**e** was used for the analyses in **f**–**i** (that is, *n* = 7 mice per treatment group for WiDr and KM20 cell line xenografts and *n* = 8 mice per treatment group for PDX models 1 and 2). Source data

**Extended Data Fig. 3:**
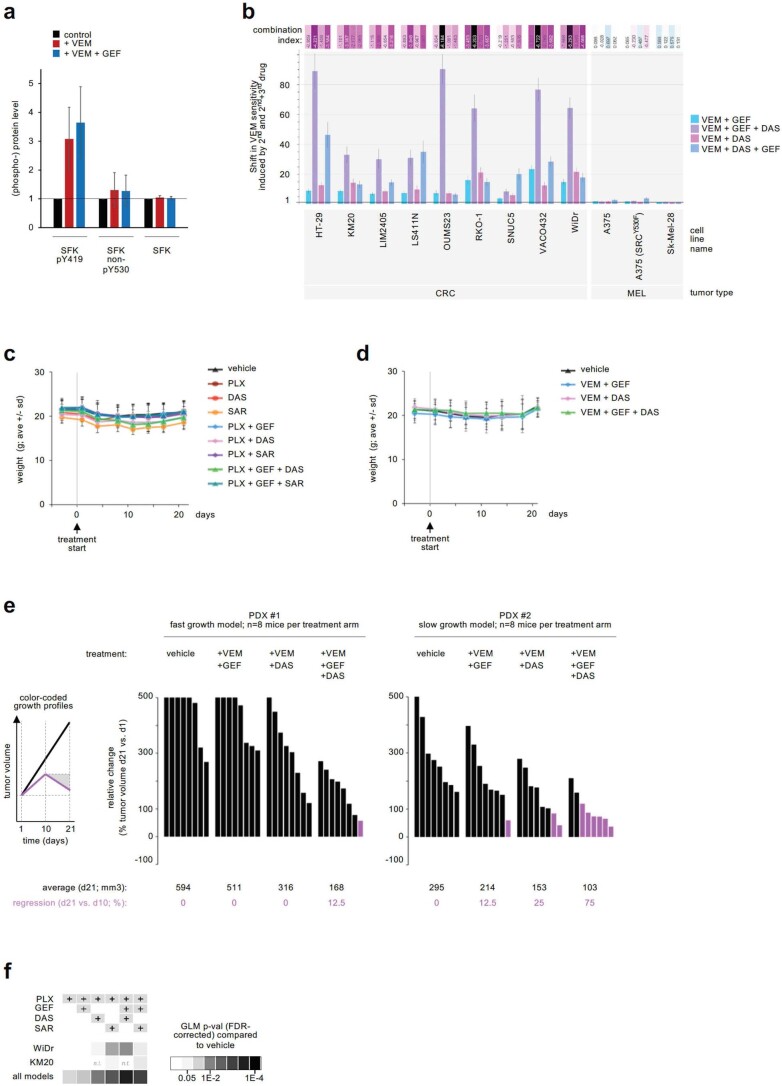
Coordinated targeting of SRC with BRAF ± EGFR improves efficacy in BRAF^V600E^ CRC cell lines and xenografts without increasing toxicity. **a**, Levels of pY419, non-pY530, and total SFK measured by western blot in BRAF^V600E^ CRC cell lines treated with vemurafenib (VEM) ± gefitinib (GEF) were quantitated. Data are normalized to control untreated per cell line and displayed as average ± standard deviation measured across HT29, KM20, LIM2405, WiDr (data available in spreadsheet ‘Fig. 3a’ of the Source Data document; n = 12 independent experiments). **b**, Shift in vemurafenib (VEM) sensitivity, measured via cell viability assays and calculation of the combination index (CI score; top panel) upon treatment of BRAF^V600E^ CRC or melanoma (MEL) cell lines with VEM + gefitinib (GEF) ± dasatinib (DAS) and VEM + DAS ± GEF, for three days. The addition of DAS to VEM + GEF increases sensitivity to VEM to a greater extent than the addition of GEF to VEM + DAS, highlighting the contribution of SFK and supporting that SFK activation upon VEM treatment is EGFR-independent. Same methods as in Fig. 2b and Extended Data Fig. 2a (n = 4 independent experiments per cell line). **c** and **d**, Mouse weight as a surrogate for toxicity following treatment of BRAF^V600E^ CRC cell line xenografts (**c**) (n = 14 mice per group), or patent-derived xenografts (**d**) (n = 16 mice per group), with vehicle control or the inhibitors listed. Data is displayed as the average weight in grams ± standard deviation. **e**, Tumor growth inhibition in BRAF^V600E^ CRC PDX models following treatment with VEM ± GEF ± DAS or vehicle (control). Waterfall plots show the relative change in tumor volume: each bar represents one tumor; and the height of the bar compares the final volume at day 21 to the starting volume at day 1. Volume changes are capped at 5-fold of the starting volume (that is 500%). Average final tumor volumes per treatment group are indicated underneath the graph (black font). Student t-test, two-sided, p-values are indicated when p < 0.05. Tumors that regressed by day 21 compared to volume at mid-treatment (that is, day 10) are shown in purple; percentages of regressing tumors per group are indicated underneath the graph. **f**, The GLM p-values corrected for false discovery rate (FDR) corresponding to the main Fig. 3f, are shown (n = 8 mice per treatment group per PDX model).

Because a BRAF + EGFR inhibitor doublet was the first FDA-approved molecularly targeted regimen for patients with BRAF^V600E^ mCRC^5^, we next asked whether the efficacy of BRAF and EGFR targeting can be improved by addition of a SRC inhibitor in vitro and in vivo. To begin, we verified SFK activation after vemurafenib + gefitinib treatment in a panel of BRAF^V600E^ CRC cell lines (Fig. 3a and Extended Data Fig. 3a). The analysis showed increased SFK activation, reflected by increased Y419 phosphorylation and Y530 dephosphorylation, which further substantiates the findings using HT-KAM in Fig. 1b–d. Moreover, triplet therapy with the addition of dasatinib to vemurafenib and gefitinib resulted in a synergistic increase in sensitivity to vemurafenib, greater than what was observed for either vemurafenib-containing doublet (Fig. 3b and Extended Data Fig. 3b). The synergistic impact on cell viability with the addition of a SRC inhibitor to BRAF + EGFR targeting was conserved across BRAF^V600E^ CRC cell lines, but not BRAF^V600E^ melanoma cells. Likewise, triplet treatment with vemurafenib plus gefitinib and dasatinib more effectively inhibited colony formation in BRAF^V600E^ CRC cells as compared to vemurafenib + gefitinib (Fig. 3c). Encouraged by these data, we tested combinations of BRAF ± EGFR and SRC inhibitors first in cell line-derived xenografts (Fig. 3d) and then in the same PDX models evaluated in Fig. 1 (Fig. 3e). In all four xenograft models, triplet combinations resulted in statistically significantly improved tumor growth inhibition as compared to BRAF + EGFR or BRAF + SRC inhibitor doublets. Toxicity, as assessed by mouse weight and distress, was negligible (Extended Data Fig. 3c,d). Moreover, tumor regression was observed beyond the midpoint of treatment in multiple PDX tumors treated with dasatinib-containing regimens (Extended Data Fig. 3e). Next, we used a generalized linear model (GLM) to quantify the effects of the drug combinations versus vehicle over time (Fig. 3f–i). On the basis of the results in Fig. 3d–i, we found that addition of a SRC inhibitor to a BRAF inhibitor had an equivalent or better effect on tumor growth inhibition in comparison to adding an EGFR inhibitor to a BRAF inhibitor. Furthermore, triple therapy with BRAF + EGFR + SRC inhibitors significantly improved tumor growth inhibition compared to any doublet (Fig. 3f,g; GLM standard coefficients >0.8 and >0.5 for cell line xenografts and PDXs, respectively; false discovery rate (FDR)-corrected *P* values in Extended Data Fig. 3f and in the right panel of Fig. 3g). Figure 3h,i highlights that the addition of a SRC inhibitor to a BRAF inhibitor + EGFR inhibitor systematically and significantly improves effect size in both cell line and PDX models.

### SRC regulates β-catenin transcriptional reprogramming

**Extended Data Fig. 4:**
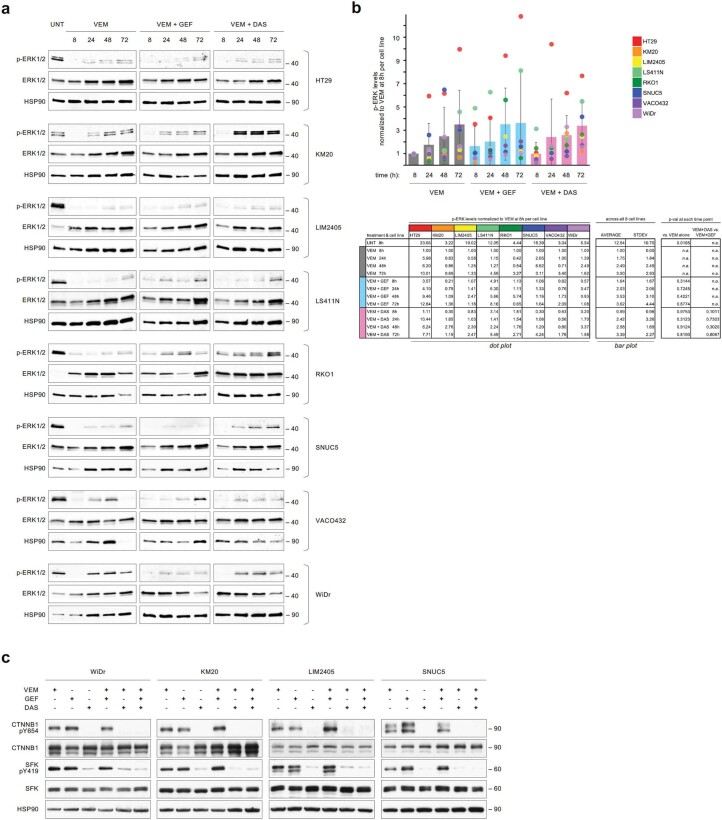
Mechanisms underlying the synergistic effects of co-targeting SRC and the BRAF ± EGFR pathways. **a**, Western blots to detect phospho-T202/Y204 ERK1/2 and total ERK1/2 in BRAF^V600E^ CRC cell lines treated with vemurafenib (VEM) ± gefitinib (GEF) or dasatinib (DAS) collected after 8 h, 24 h, 48 h or 72 h. HSP90 is used as a loading control. The experiment was repeated 2 independent times with similar results. **b**, Quantification of western blots shown in panel (**a**). The bar plot (averages and standard deviations per treatment condition across cell lines) was overlaid with a dot plot displaying individual measurements per cell line and condition. Data are normalized to p-ERK levels after 8 h treatment with VEM alone. See table below for detailed values and color codes; n = 8 cell lines. **c**, Western blots to detect total and phospho-Y654 beta-catenin (CTNNB1) in BRAF^V600E^ CRC cell lines treated with VEM, or GEF, or DAS, or combinations of VEM + GEF, or VEM + DAS, or VEM + GEF + DAS. The detection of phospho-Y419 and total SFK serves as a control for the effect of SFK-inhibition (with DAS). The experiment was repeated 3 independent times with similar results. In panels **a**, **c**, molecular weight/size markers are indicated on the right (kDa). Source data

Next, we sought to elucidate the mechanism underlying the synergistic effects of co-targeting SRC and the MAPK pathway ± EGFR shown in Figs. 2 and 3. MAPK signaling rebound is recognized as an important mechanism of resistance^12,13^, so we tested whether adding the SRC inhibitor dasatinib would inhibit phospho-ERK rebound more profoundly than BRAF inhibitor alone or BRAF inhibitor + EGFR inhibitor. Using eight BRAF^V600E^ CRC cell lines collected at four different times (up to 72 h) with BRAF inhibitor, BRAF inhibitor + EGFR inhibitor or BRAF inhibitor + SRC inhibitor, we found that SRC inhibition did not significantly impact rebound of ERK phosphorylation (Extended Data Fig. 4a,b). This suggests that SRC activation in response to BRAF ± EGFR targeted therapy acts through a distinct mechanism of drug resistance.

**Fig. 4:**
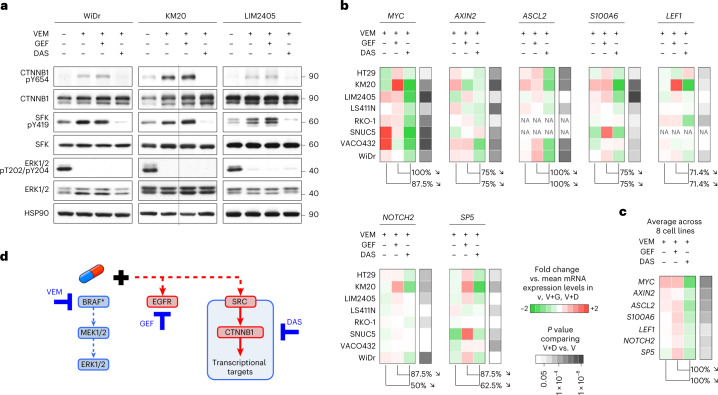
SRC regulates the phosphorylation of β-catenin. **a**, Western blots of BRAF^V600E^ CRC cell lines treated with vemurafenib ± gefitinib or dasatinib. The Y654 residue of CTNNB1 is a reported phospho-target site of SRC kinases^8^. ERK1/ERK2 phospho-T202/Y204 serves as a control for the effect of BRAF inhibition (with vemurafenib). SFK pY419 serves as a control for the effect of SFK inhibition (with dasatinib). Molecular weight/size markers are indicated on the right (kDa). The experiment was repeated three independent times with similar results. **b**, Color-coded expression levels of β-catenin target genes (*MYC*, *AXIN2*, *ASCL2*, *S100A6*, *LEF1*, *NOTCH2*, *SP5*) measured using qRT–PCR in BRAF^V600E^ CRC cell lines treated with vemurafenib ± gefitinib or dasatinib. Expression profiles are shown as fold change against the mean mRNA expression level with vemurafenib, vemurafenib + gefitinib, vemurafenib + dasatinib. Percentages indicate the proportion of measurements across eight cell lines where the expression of the indicated gene (top) was lower with vemurafenib + dasatinib than with vemurafenib + gefitinib or vemurafenib alone. The right-most columns indicate *P* values (Student’s *t* test) comparing gene expression for vemurafenib + dasatinib versus vemurafenib alone. NA, not available due to expression levels that were too low. *n* ≥ 3 independent experiments. **c**, The expression profiles in **b** averaged across all eight cell lines (*n* = 4 independent experiments). Exact *P* values for **b**,**c** are available as Source Data. **d**, Proposed mechanism regulated by SRC that drives resistance to BRAF/MEK/EGFR therapies in BRAF^V600E^ CRC. Source data

Because many kinases can propagate their pro-oncogenic activities through transcriptional reprogramming, we hypothesized that SRC kinases may regulate the phosphorylation state of transcription factors involved in the compensatory response to BRAF ± EGFR inhibition. We first used the PhosphoAtlas kinase–substrate interaction database^8^ to identify candidate transcription factor targets of SRC; we then assessed protein phosphorylation profiles by western blot. We found that the phosphorylation of β-catenin (CTNNB1) at Y654, which is an understudied phospho-target site of SRC kinases^19^, was increased upon BRAF inhibitor or BRAF inhibitor + EGFR inhibitor treatment, but was strongly decreased upon BRAF inhibitor + SRC inhibitor treatment (Fig. 4a and Extended Data Fig. 4c). To assess whether these changes in CTNNB1 Y654 phosphorylation impact the transcriptional activities of CTNNB1, we measured the expression levels of a series of CTNNB1 target genes^20^ using quantitative reverse transcription PCR (qRT–PCR). We found that adding the SRC inhibitor dasatinib to BRAF inhibitor led to a significant decrease in the mRNA levels of all β-catenin target genes we tested (that is, *MYC*, *AXIN2*, *ASCL2*, *S100A6*, *LEF1*, *NOTCH2* and *SP5*) in comparison to cells treated with BRAF inhibitor alone or BRAF inhibitor + EGFR inhibitor (Fig. 4b,c). This indicates that the activation of SRC kinases upon BRAF ± EGFR targeting regulates the function of β-catenin, a crucial transcription factor in CRC tumorigenesis, which can in turn drive transcriptional reprogramming of cells to sustain and adapt to therapeutic pressure. Altogether, SRC activation induces a tumor survival mechanism that acts in parallel to both the MAPK and EGFR signaling axes in BRAF^V600E^ CRC (Fig. 4d).

### COX2–PGE2 upregulation mediates SRC activation

**Fig. 5:**
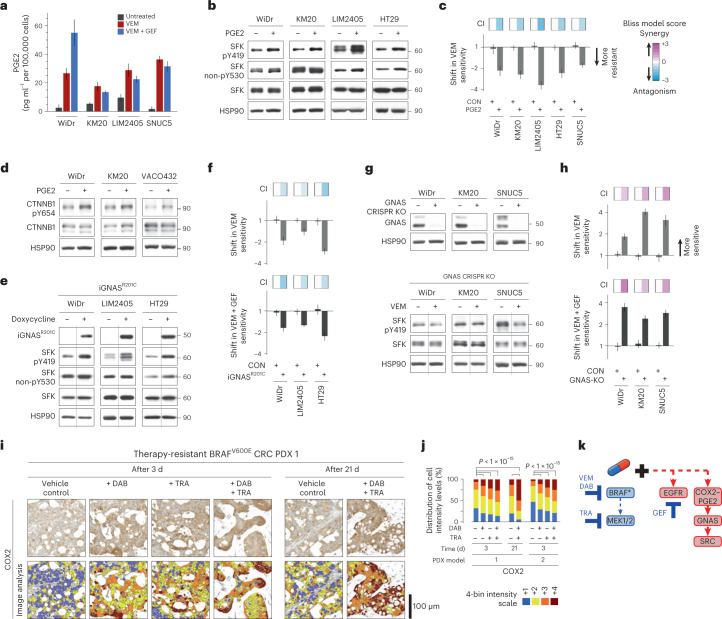
COX2–PGE2 upregulation mediates SRC activation in BRAF^V600E^ CRC cell lines and PDXs. **a**, Levels of secreted PGE2 were measured by ELISA in the conditioned medium of BRAF^V600E^ CRC cell lines treated with vemurafenib ± gefitinib. Data are displayed as the average PGE2 secretion in pg ml^–1^ per 100,000 cells ± s.d. (*n* = 3 independent experiments per cell line). **b**, BRAF^V600E^ CRC cell lines were treated with exogenous PGE2. Cell lysates were assayed by western blot as indicated. Y419 phosphorylation and lack of phosphorylation of Y530 (non-pY530) are used as readouts of SFK activation. HSP90 serves as a loading control. The experiment was repeated two independent times per cell line with similar results. **c**, Bar graphs representing fold change (log_2_ scale) ± standard error for change in sensitivity to vemurafenib upon further treatment with PGE2 or untreated control in 3-day cell viability assays. Top, CI, Bliss model. Same methods as in Fig. 2b (*n* = 3 independent experiments per cell line). **d**, Western blots to detect pY654 of CTNNB1 in BRAF^V600E^ CRC cell lines treated with exogenous PGE2. The experiment was repeated two independent times per cell line with similar results. **e**, Three BRAF^V600E^ CRC cell lines engineered with a doxycycline-inducible constitutively active GNAS construct, iGNAS^R201C^, were treated with doxycycline. Cell lysates were assayed by western blot as indicated. The experiment was repeated three times with similar results. **f**, Bar graphs representing fold change (log_2_ scale) ± standard error for change in sensitivity to vemurafenib or vemurafenib + gefitinib after iGNAS^R201C^ induction in 3-day cell viability assays. Top, CI, as in **c** (*n* = 3 independent experiments per cell line). **g**, GNAS was knocked out in BRAF^V600E^ CRC cells using CRISPR (GNAS-KO). GNAS knockout was validated by western blot (top). GNAS-KO cells were treated with vemurafenib, and cell lysates were assayed by western blot with the indicated antibodies (bottom). The experiment was repeated ≥2 times with similar results. In **b**,**d**,**e**,**g**, molecular weight/size markers are indicated on the right (kDa). **h**, Bar graphs representing fold change (log_2_ scale) ± standard error for change in sensitivity to vemurafenib or vemurafenib + gefitinib with GNAS knockout in 3-day cell viability assays. Top, CI, as in **c** (*n* = 3 independent experiments per cell line). **i**, Representative IHC images showing COX2 staining intensity following treatment of a BRAF^V600E^ CRC PDX model with vehicle control, dabrafenib and/or trametinib for 3 or 21 d (where *n* is the same as defined in Fig. 1e). The color-coded bottom panel highlights differences in bin intensities from automated image analysis (see Methods for details). **j**, Quantification of COX2 staining intensity by IHC for two PDX models treated for 3 or 21 d with dabrafenib ± trametinib versus vehicle control (two-sided Student’s *t* test, *P* < 1 × 10^–15^; *n* is the same as defined in Fig. 1f). **k**, Proposed mechanism of COX2–PGE2-mediated SRC-driven resistance to BRAF/MEK/EGFR therapies in BRAF^V600E^ CRC. Source data

Acknowledging that addition of a SRC inhibitor to BRAF ± MEK ± EGFR targeted therapies for treatment of BRAF^V600E^ mCRC is unlikely to be clinically acceptable owing to concerns about toxicity in patients, we asked whether characterization of upstream effectors of SFK could lead to a more clinically appropriate regimen. It has been reported previously that prostaglandin E_2_ (PGE2) can induce activation of SRC in CRC cells, without specific attention to *BRAF*^V600E^ mutation status^21,22^. Using a panel of BRAF^V600E^ CRC cell lines, an increase in PGE2 levels in the medium was consistently found as a result of BRAF ± EGFR inhibition (Fig. 5a). Treatment of BRAF^V600E^ CRC cells with PGE2 led to an increase in SFK activation (Fig. 5b) and a two- to fourfold increase in resistance to vemurafenib, with CI scores indicating antagonism (Fig. 5c). In line with the SRC–β-catenin signaling cascade established in Fig. 4, we found that PGE2 treatment led to an increase in phosphorylation of CTNNB1 Y654 (Fig. 5d).

**Extended Data Fig. 5:**
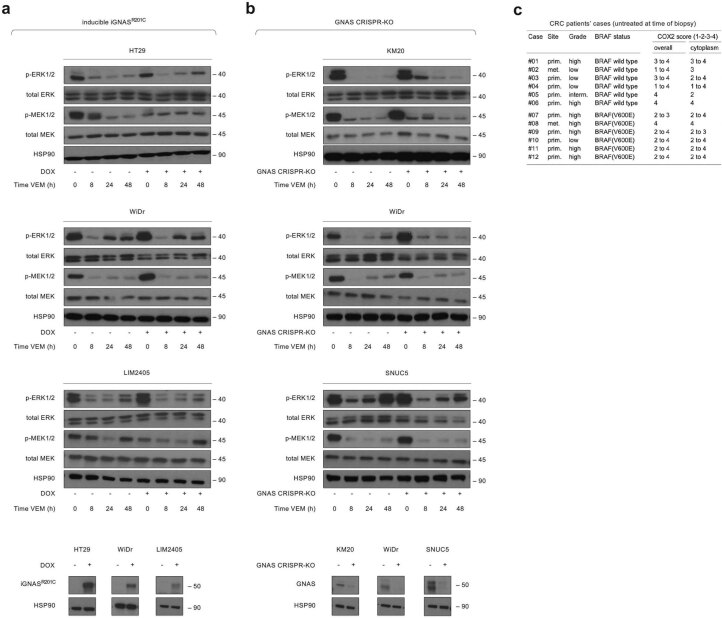
GNAS signaling in BRAF^V600E^ CRC cell lines and tumors. **a-b**, Western blots to detect phospho-T202/Y204 ERK1/2, total ERK1/2, phospho-S217/S221 MEK1/2, total MEK1/2 in BRAF^V600E^ CRC cell lines modified for GNAS expression, and treated with vemurafenib (VEM). The experiment was repeated 2 independent times per cell line with similar results. **a**, BRAF^V600E^ CRC cell lines engineered with a doxycycline-inducible constitutively active GNAS construct, iGNAS^R201C^ with or without doxycycline treatment (+ or – at the bottom of each panel). **b**, BRAF^V600E^ CRC cell lines knocked out for GNAS using CRISPR (GNAS-KO or CRIPSR control; indicated as + or – at the bottom of each panel). In panels **a**-**b**, molecular weight/size markers are indicated on the right (kDa). **c**, COX2 staining score by IHC in untreated patient CRC tumor specimens with or without a BRAF^V600E^ mutation, from primary (prim.) or metastatic (met.) sites. Source data

High levels of PGE2 promote tumor growth by eliciting aberrant extracellular signaling through its G-protein-coupled receptors (GPCRs), EP2 and EP4, and their key downstream effector, GNAS^21,23–25^. To mimic the effect of PGE2, we engineered three BRAF^V600E^ CRC cell lines with doxycycline-inducible expression of a constitutively active GNAS mutant (GNAS^R201C^). When these cells were treated with doxycycline, GNAS^R201C^ was induced, leading to increased SFK activation (Fig. 5e). Induction of GNAS also rendered the cells more resistant to vemurafenib ± gefitinib treatment; CI scores again indicated antagonism (Fig. 5f). On the other hand, suppressing GNAS expression by CRISPR knockout of GNAS in BRAF^V600E^ CRC cell lines (GNAS-KO) prevented SFK activation after vemurafenib treatment (Fig. 5g). GNAS-KO cells displayed increased sensitivity to BRAF ± EGFR inhibition, with CI scores showing synergy (Fig. 5h), indicating that treatment-dependent SFK activation in BRAF^V600E^ CRC cells is downstream of PGE2–GNAS signaling. We noted that neither GNAS^R201C^-induced activation of SFK nor GNAS CRISPR knockout-induced inhibition of SFK activity impacted rebound of ERK phosphorylation (Extended Data Fig. 5a,b), further indicating that feedback activation of the PGE2–GNAS–SRC signaling axis acts in concert with the MAPK cascade to drive therapeutic resistance.

It is well established that PGE2 expression and secretion are regulated by COX2 (ref. ^26^). COX2 upregulation in BRAF^V600E^ CRC PDX tumors treated with dabrafenib + trametinib for 3 or 21 d was corroborated by IHC (Fig. 5i,j), which parallels what was observed with SRC in these same PDX persists in residual tumors even at late time points. COX2 levels were similar in untreated primary patient colorectal tumor specimens with or without a *BRAF*^V600E^ mutation (Extended Data Fig. 5c), again corresponding to observations with SRC (Extended Data Fig. 1d). In summary, these findings suggest that, in BRAF^V600E^ CRC, COX2–SRC–β-catenin signaling does not overlap with EGFR–BRAF–MAPK signaling and it is treatment with BRAF ± MEK or EGFR targeted therapies that triggers the compensatory upregulation of a pre-existing COX2–PGE2–GPCR–GNAS autocrine loop, which in turn activates SRC (Fig. 5k).

### COX2 inhibition synergizes with BRAF/MEK/EGFR targeting

**Fig. 6:**
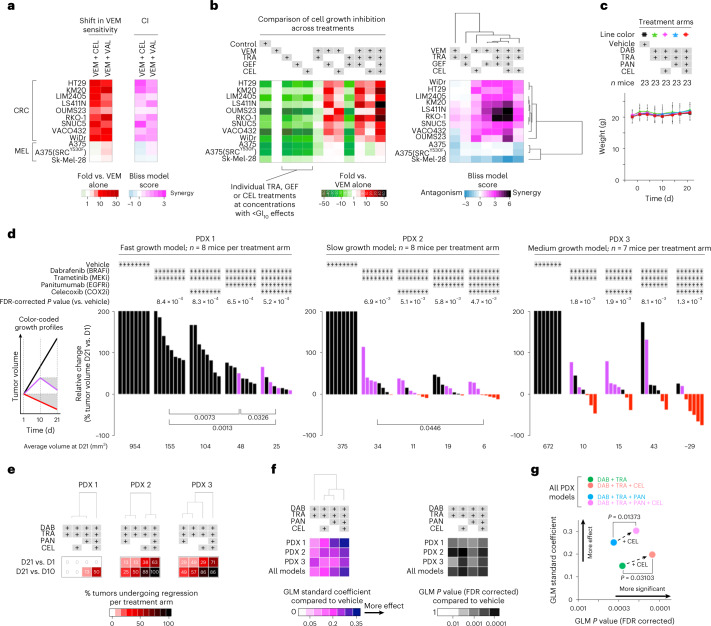
Coordinated targeting of COX2 with BRAF/MEK/EGFR improves efficacy in BRAF^V600E^ CRC cell lines and PDXs. **a**, Shift in vemurafenib sensitivity measured by cell viability assay (left) and calculation of CI (right) upon treatment of BRAF^V600E^ CRC or melanoma cell lines with vemurafenib together with a COX2 inhibitor (celecoxib (CEL) or valdecoxib (VAL)) for 3 d. CI is averaged from experimentally measured CIs at 1×GI_50_, 2×GI_50_ and 0.5×GI_50_ concentrations of each drug (*n* ≥ 2 independent experiments). **b**, Treatment of BRAF^V600E^ CRC or melanoma cell lines with up to four inhibitors, including trametinib, gefitinib and celecoxib at GI_10_. Cell growth inhibition across treatment permutations, normalized to vemurafenib monotherapy (left), was used to calculate CI relative to all other treatment arms and subjected to unsupervised hierarchical clustering comparing cell lines and treatment arms (right) (*n* = 24 independent experiments). **c**, Mouse weight as a surrogate for toxicity following treatment of BRAF^V600E^ CRC PDXs (23 mice per treatment arm) with vehicle control, dabrafenib, trametinib, celecoxib and/or panitumumab (PAN). Data are displayed as the average weight in grams ± s.d. **d**, Tumor growth inhibition in BRAF^V600E^ CRC PDX models following treatment with dabrafenib + trametinib ± celecoxib ± panitumumab or vehicle (control). Waterfall plots show the relative change in tumor volume: each bar represents one tumor, and the height of the bar compares the final volume at day 21 (D21) to the starting volume at day 1 (D1). Volume changes are capped at twofold the starting volume (that is, 200%). Tumors that regressed by day 21 are shown in red (compared to the volume at day 1) and purple (compared to the volume at mid-treatment, that is, day 10). Average final tumor volumes per treatment group are indicated underneath the graphs (black font). *P* values are indicated from two-sided Student’s *t* tests when *P* < 0.05. All raw and relative tumor volumes are available as Source Data. **e**, Semisupervised hierarchical clustering of the percentages of regressing tumors per treatment arm, comparing day 21 versus day 1 and day 21 versus day 10, from the data shown in **d** (that is, same number (*n*) of mice per treatment group and per PDX as in **d**). **f**, GLMs to test the association of change in tumor volume between treatment arms and vehicle over time. A GLM was applied to each PDX model, and all PDXs were combined. Left, effect size measured as the GLM standard coefficient; semi-unsupervised hierarchical clustering further compares the efficacy of the treatment arms. Right, ranking by GLM *P* values corrected for FDR. **g**, Comparison of effect size and FDR-corrected *P* values for treatment arms with and without the addition of celecoxib. Analyses in **f**,**g** used the same number (*n*) of mice per group as in **d**. Source data

COX2 is a rational drug target, given its robust association with CRC tumor progression in patients^27^, although no prior clinical trials have focused on BRAF^V600E^ CRC. COX2 inhibitors also represent a practical alternative to SRC-targeting therapies: the COX2 inhibitor celecoxib is FDA approved, has a favorable side-effect profile and is relatively inexpensive. Thus, the logical next step was to test BRAF-targeted therapies in combination with COX2 inhibition. As proof of concept, addition of a COX2 inhibitor produced a consistent, synergistic increase in sensitivity to vemurafenib across a panel of BRAF^V600E^ CRC cell lines, which was not recapitulated in BRAF^V600E^ melanoma (Fig. 6a). Using very low individual drug concentrations for trametinib, gefitinib and celecoxib (GI_10_ or below), we were able to demonstrate potentiation of cell growth inhibition with combinations of up to four targeted therapies (Fig. 6b, left). Addition of celecoxib systematically improved the efficacy of—and synergized with—vemurafenib + trametinib ± gefitinib (Fig. 6b, right). The most synergistic combination, across BRAF^V600E^ CRC but not melanoma cell lines, was the quadruple-treatment arm.

Next, we tested whether addition of celecoxib could improve upon two clinical benchmark regimens: the dabrafenib + trametinib doublet received by the patients from whom the PDX models were derived^4^ and a triplet regimen with addition of the anti-EGFR antibody panitumumab, which was tested in a subsequent clinical trial^6^. Toxicology studies were conducted before efficacy testing: mouse weight, a surrogate for drug toxicity, remained stable over the course of therapy for all inhibitor combinations (Fig. 6c). The critical finding from the efficacy studies was that addition of celecoxib to the clinical trial-tested doublet and triplet drug regimens resulted in consistently superior tumor growth inhibition in all three BRAF^V600E^ CRC PDX models (Fig. 6d). The majority of tumors treated with celecoxib in addition to dabrafenib + trametinib ± panitumumab exhibited regression by the second half of the 21-day treatment course (Fig. 6d,e). It is notable that 78% of tumors subjected to quadruple therapy with celecoxib exhibited regression (red and purple bars), versus 30% of tumors regressing in the no-celecoxib triplet-therapy counterpart arm. Across models, quadruple treatment resulted in the most statistically significant tumor growth inhibition (Fig. 6d; *P* values above the bar graphs).

When applying the GLM approach across PDX models, we found that the addition of celecoxib systematically increased effect size; the greatest effect size was observed for the quadruple therapy (Fig. 6f, left, and Fig. 6g, *y* axis). Moreover, addition of celecoxib to any treatment arm resulted in statistically significant improvements in tumor growth inhibition (Fig. 6f, right, and Fig. 6g, *x*-axis FDR-corrected *P* values and arrows).

### Addition of celecoxib causes durable tumor growth inhibition

**Fig. 7:**
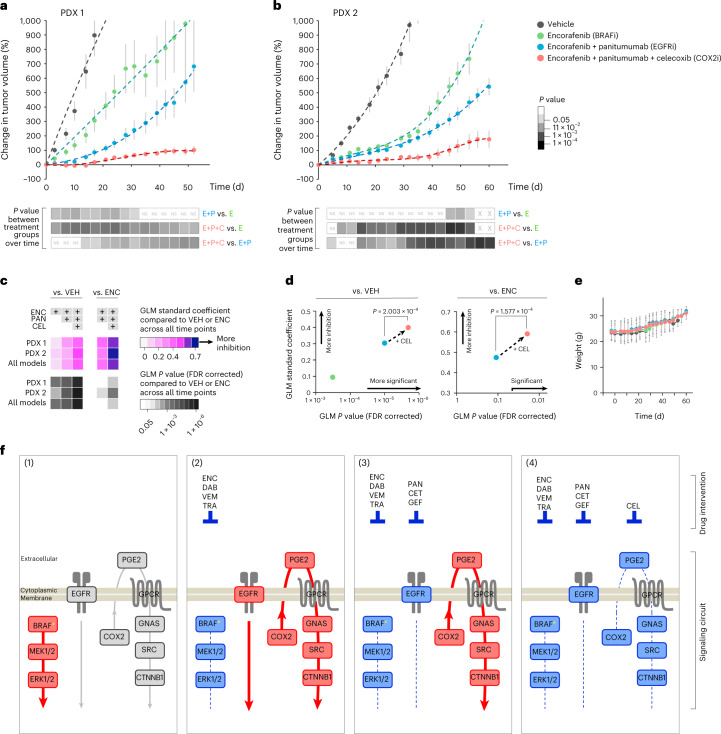
Coordinated targeting of COX2 with BRAF + EGFR improves long-term efficacy in BRAF^V600E^ CRC PDXs. **a**,**b**, Tumor growth profiles in BRAF^V600E^ CRC PDX models 1 and 2 treated with encorafenib (ENC) ± panitumumab ± celecoxib or vehicle (VEH; control). Changes in tumor volume relative to starting volume at day 1 (average ± standard error) are plotted over time. *P* values from two-sided Student’s *t* tests across all time points comparing treatment arms are shown as a grayscale underneath each graph. NS, not significant; X, not available. All raw and relative tumor volumes are available as Source Data. In **a**, *n* = 11 mice per treatment group; in **b**: VEH, *n* = 5 mice; ENC, *n* = 6 mice; ENC + PAN, *n* = 9 mice; ENC + PAN + CEL, *n* = 9 mice. **c**, GLMs to test the association of change in tumor volume over time, either between treatment arms and vehicle (left) or between combination therapy and encorafenib alone (right). A GLM was applied to each individual PDX model and to both PDXs combined. Top, effect size measured as the GLM standard coefficient comparing the efficacy of the treatment arms. Bottom, GLM FDR-corrected *P* values. **d**, Comparison of effect sizes and FDR-corrected *P* values of treatment arms with and without celecoxib, using vehicle or encorafenib treatment as the baseline (left and right, respectively). The same number of mice per group shown in **a**,**b** was used for the analyses in **c**,**d**. **e**, Mouse weight as a surrogate for treatment toxicity. Data are displayed as the average weight in grams ± s.d. All weights from the results shown in **a**,**b** were used: VEH, *n* = 16 mice; ENC, *n* = 17 mice; ENC + PAN, *n* = 20 mice; ENC + PAN + CEL, *n* = 20 mice. **f**, Schematic summary of the states of signaling pathways depending on treatment: (1) untreated tumors, with BRAF–MEK–ERK as the main driver of progression (red) and baseline activity of the EGFR and COX2–SRC signaling pathways (gray), and (2–4) tumors treated with drugs (listed on top) inhibiting the activity (blue) of the three distinct signaling axes: BRAF–MEK, EGFR and COX2–SRC–β-catenin In scenario (4), triple treatment collectively blocks the cooperative dependencies that drive resistance and progression. Source data

The findings in Fig. 6d prompted us to assess the durability of treatment effects in the two most drug-resistant PDXs (PDX models 1 and 2 in Fig. 6d). We measured changes in tumor volume for >50 d in mice treated with encorafenib (BRAF inhibitor) ± panitumumab (EGFR inhibitor) ± celecoxib (COX2 inhibitor) (Fig. 7a,b). We found that the triple treatment significantly improved tumor growth inhibition as compared to the dual drug combination, which is a current standard of care^5^ (*P* values across measurements underneath the graphs in Fig. 7a,b). This was confirmed by GLM analysis considering all time points and individual tumor volumes (Fig. 7c,d). Increased toxicity was not observed with the addition of celecoxib (Fig. 7e). These results indicate that COX2 inhibition represents a novel, low-cost and low-toxicity drug-repurposing strategy to overcome therapeutic resistance in BRAF^V600E^ CRC: supplementing standard-of-care encorafenib + panitumumab with celecoxib durably improves tumor growth inhibition.

## Discussion

Despite recent optimization of targeted therapy combinations^3–6^, *BRAF*^V600E^ mutation still predicts the form of mCRC with the worst prognosis. Thus, we endeavored to uncover orthogonal mediators of compensatory resistance to BRAF-, MEK- and EGFR-targeted inhibitors tested in patients. We discovered that SRC kinases act as a nexus of adaptive, druggable and EGFR-independent therapeutic resistance in vitro and in vivo. Our findings were reproducible across a variety of inhibitors in the same class, cell lines and mouse models, and yet were specific to BRAF^V600E^ mCRC. Activation of SRC in response to BRAF ± MEK or EGFR therapies did not contribute to MAPK signaling rebound. Instead, we found that SRC activation regulates transcriptional reprogramming through β-catenin activation and is mediated by an upstream pro-inflammatory pathway involving COX2. Addition of celecoxib to inhibitor combinations tested in clinical trials consistently resulted in superior and durable tumor growth suppression in BRAF^V600E^ CRC PDX models. Our study identified unanticipated cooperative dependencies of actionable targets, yielding strategies to overcome therapeutic resistance (summarized in Fig. 7f).

*BRAF*^V600E^ is a classic example of how the same activating mutation can have different roles depending on the cancer subtype-specific signaling context. BRAF inhibitors have produced an impressive response rate in BRAF^V600E^ melanoma, but not in CRC^3^. In CRC, synthetic lethality genetic dropout screens originally found that feedback activation of EGFR promotes intrinsic resistance to BRAF inhibition^12–14,28^. However, modest responses in patients treated with BRAF + EGFR combination therapy underline why, in situations where predicting therapeutic response cannot be reduced to a single genetic dependency, there is a role for functional proteomic approaches designed to more comprehensively reveal crosstalk between signaling pathways and to better dissect the dynamic processes induced by drug interventions^29–32^. Here we used the HT-KAM platform to directly capture the phospho-catalytic fingerprint of kinases in biological extracts and to identify ranked drug susceptibilities. This approach elucidated how the concerted rewiring of interdependent signaling pathways drives resistance to BRAF-, MEK- and EGFR-targeted therapies. SRC was identified as a central, conserved mediator of these signaling circuits in BRAF^V600E^ CRC.

SRC kinases are a non-receptor protein tyrosine kinase (NRTK) family of essential pleiotropic mediators of signaling cascades that connect extracellular cues to intracellular programs^17,18,33^. Knowing that SRC often acts downstream of receptor protein tyrosine kinases (RTKs), including in the context of acquired resistance to RAF inhibition^34,35^, one might expect SRC to be activated by EGFR in response to BRAF-targeted therapy^12,13,28^. Unexpectedly, however, in BRAF^V600E^ CRC, we found that SRC kinases function as integral components of a drug resistance circuit that is triggered independently of EGFR. Specifically, BRAF^V600E^ CRC cells adapt to targeted therapy by relying on a separate inflammatory loop that funnels through SRC. Possibly even more surprisingly, our in vivo and in vitro observations (Fig. 3) indicate that SRC may have a more dominant role than EGFR in the context of BRAF^V600E^ CRC. In fact, previously discovered transactivation effects of SRC on EGFR through intra- and extracellular mechanisms^23,36^ show that SRC acts as an upstream effector of EGFR. A SRC-driven transactivation mechanism would provide an alternative route to the previously noted feedback release activation of EGFR through reduced CDC25C phosphatase activity that is initiated by therapeutic inhibition of BRAF/MEK^13^. Together, CDC25C and SRC could functionally complement each other by converging on EGFR to coordinate its activation upon BRAF/MEK inhibition.

Although SRC kinases are known direct upstream effectors of c-RAF that regulate the signaling activity of RAF homo- and heterodimers^37–40^, inhibition of SRC did not impact the ERK rebound commonly associated with RAF therapy resistance. A SRC-driven drug-bypass mechanism might still explain how tumors can efficiently evade RAF targeting without acquired resistance mutations. Moreover, SRC kinases can directly promote the activity of other kinases involved in drug resistance, including AKT1 (ref. ^41^), an essential mediator of EGFR signaling. BRAF^V600E^ CRC cells may thus compensate for the strain of EGFR targeting through SRC, releasing cells from their dependency on EGFR to adapt to BRAF + EGFR combination therapies.

In addition to these direct regulatory effects on downstream kinases, SRC kinases are also known to propagate their pro-oncogenic activities through networks of transcription factors^42–44^. In fact, we found that SRC phosphorylates an understudied phospho-site of CTNNB1 (Y654). Upon BRAF ± EGFR inhibition, SRC-dependent phosphorylation of Y654 increases β-catenin’s transcriptional activity, leading to a reprogramming of the transcriptional profiles of BRAF^V600E^ CRC cells. The WNT–β-catenin signaling pathway is a key driver in the initiation and progression of CRC, differentiating it from other cancers, including *BRAF*-mutated melanoma. Such a SRC-driven reprogramming mechanism could rapidly and durably rewire signaling pathways in BRAF^V600E^ CRC cells, effectively promoting adjustment to therapeutic stress.

The signaling plasticity offered by these SRC-dependent mechanisms may usurp and/or reinforce cell-autonomous pathways that drive tumor survival while bypassing other dependencies, including under the influence of BRAF/MEK or EGFR targeted therapies. Altogether, this argues that blocking the pathways that activate SRC kinases represents a logical strategy to reinforce the inhibition of both the RAF–MEK–ERK and EGFR–PI3K–AKT axes and to prevent the emergence of a therapeutic resilience phenotype (Fig. 7f).

FDA-approved SRC inhibitors are available; however, toxicity in combination with other targeted agents is a major concern. This prompted us to determine what upstream pathways activate SRC to potentially leverage these mechanisms as clinically actionable targets. Our data show that PGE2 signaling drives SRC activation in BRAF^V600E^ CRC. Despite decades of work on SRC, its contribution as a regulator of cancer-related inflammation has remained largely unexplored. Here we demonstrated that BRAF^V600E^ CRC cells overcome BRAF ± MEK or EGFR therapies by upregulating a prosurvival, autocrine/oncocrine COX2–PGE2–GNAS–SRC–β-catenin signaling loop. This adaptive response was not observed in BRAF^V600E^ melanoma cells, highlighting how SRC is embedded in pre-existing signaling networks specific to BRAF^V600E^ CRC. Of note, >90% of CRC tumors also harbor alterations in the WNT signaling pathway, typically an initiating *APC* mutation^45^. Kinase circuits and drug response mechanisms are inevitably adapted to the WNT/APC-mutated background of BRAF^V600E^ CRC cells. Both the WNT and G-protein-regulated signaling networks are induced by extracellular inflammatory cues, and the two pathways share many intracellular signaling components, such as GSK3β or β-catenin^23,46^. This inherent predisposition of BRAF^V600E^ CRC cells to rely on inflammatory pathways to alleviate and withstand drug pressure is underscored by our finding that the SRC-relayed PGE2 signaling cascade causes therapeutic resistance (Fig. 7f).

The production of PGE2 is regulated by the COX2 enzyme. COX2 is a rational target, implicated in intestinal inflammation and, by association, CRC initiation and progression^27,47^. Although several prior clinical trials testing SRC or COX2 inhibitors in patients with CRC have failed to show meaningful clinical activity^48–53^, no trial has yet focused on BRAF^V600E^ mCRC or evaluated these agents in combination with BRAF-targeted therapies. Recent BRAF^V600E^ mCRC clinical trials have demonstrated the feasibility of administering three targeted therapies simultaneously^5^; however, quadruple therapy pushes the limits of acceptability—unless the fourth therapy is inexpensive and has an exceptionally favorable side-effect profile. On both counts, COX2 is a more attractive drug target than SRC. We showed that adding celecoxib to a current standard-of-care treatment, encorafenib (BRAF inhibitor) + panitumumab (EGFR inhibitor)^5^, resulted in sustainable and significant tumor growth inhibition in BRAF^V600E^ CRC PDXs. Addition of the inexpensive, FDA-approved COX2 inhibitor celecoxib to BRAF-targeted therapies could be rapidly translated in patients with BRAF^V600E^ mCRC, with few anticipated side effects.

We attempted to determine retrospectively whether use of celecoxib or other nonsteroidal anti-inflammatory drugs as concomitant medications conferred benefit to patients with CRC who participated on clinical trials testing BRAF inhibitor-based therapies; however, incomplete data collection and heterogeneous dosing precluded this analysis. Additionally, while PDX models faithfully recapitulate patients’ initial responses to targeted therapies^6^, it is unknown whether they can fully predict response, especially as the mice used lack a functional immune system. Furthermore, we acknowledge that there may be alternative routes to adaptive resistance, not represented by our models, in which case it may be possible to leverage the HT-KAM platform to develop predictive biomarkers to most effectively tailor therapy.

In conclusion, our results demonstrate that SRC has a dominant role in mediating the unresponsiveness of BRAF^V600E^ colorectal tumors to BRAF inhibitors and suggest that SRC and EGFR act in conserved, complementary, parallel circuits that drive resistance and can be jointly targeted to restore therapeutic sensitivity. SRC activation is nonredundant with the MAPK pathway. We determined that a COX2 inflammatory pathway drives SRC activation; the effects of inhibiting SRC were recapitulated in vitro and in vivo by targeting COX2. These results argue that drug resistance can result from a combination of pathways that are upregulated, working in concert and interdependent of each other, such that impeding their coordinated signaling activities is necessary to overcome resistance. The HT-KAM approach can identify a finite number of key cooperative dependencies, offering a curated selection of convergent targets to evaluate. Our hope is that, by expanding the scope of investigation, BRAF^V600E^ mCRC will one day become like HER2-positive breast cancer, where what was once a poor-prognosis subtype with few treatment choices has been transformed into an opportunity to receive effective targeted therapy options.

## Methods

Our research complies with all relevant ethical regulations. The UCSF Preclinical Therapeutics Core (PTC) Laboratory and Laboratory Animal Resource Center (LARC) operate under the Institutional Animal Care and Use Committee (IACUC), approval number 194778-01. Further information on research design is available in the Nature Portfolio Reporting Summary linked to this article.

### Cell lines, cell culture conditions and genetic alterations

Cell lines were purchased from ATCC or provided by R.B.: (1) BRAF^V600E^ CRC cells: WiDr, SNUC5, HT29, Colo-205, RKO-1, LIM2405, KM20, LS411N, VACO432, SW1417; (2) CRC MAP3K8^amp^ cells: OUMS23; (3) KRAS^mut^ CRC cells: HCT116, LoVo; (4) BRAF^V600E^ melanoma cells: A375, A375 (SRC^Y530F^), A375 (myr-AKT1), Sk-Mel-28, Mel888. Cells were cultured following ATCC’s instructions or as previously described^13^.

#### Transfections

siRNA transfections were performed using Lipofectamine 2000 according to the manufacturer’s instructions (Invitrogen). Cells were processed 72 h after siRNA transfection. Control siRNAs and siRNAs targeting CSK were obtained from Dharmacon (siCON: D-001206-13-5 and D-001206-14-20; siCSK: M-003110-02).

#### Generation of stable cell lines with control and SRC knockdown

For stable knockdown of SRC, oligonucleotides containing the shRNA hairpin were annealed and then ligated into the pLKO.1 vector. The two shRNA sequences used against SRC were shSRC 1 (hairpin sequence TRCN0000199313: 5′-GCTGACAGTTTGTGGCATCTT-3′) and shSRC 2 (hairpin sequence TRCN0000195339: 5′-CATCCTCAGGAACCAACAATT-3′). Lentiviruses were produced by UCSF Viracore. WiDr, KM20, LIM2405 and SNUC5 cell lines stably expressing control shRNA and shRNAs against SRC (shCON, shSRC 1, shSRC 2) were produced by transduction with the corresponding lentiviruses in the presence of 8 µg ml^–1^ polybrene (Sigma-Aldrich). After incubation in growth medium for 72 h, cells were treated with 2 µg ml^–1^ puromycin.

#### Generation of GNAS lenti-CRISPR knockout cell lines

GNAS lenti-CRISPR knockout cancer cell lines were generated by targeting exon 1 of the human *GNAS* locus with the CRISPR–Cas9 system as previously described^54,55^. The forward and reverse sgRNA-targeting sequences used were 5′-CACCGCTACAACATGGTCATCCGGG-3′ and 5′-AAACCCCGGATGACCATGTTGTAGC-3′, respectively. Guide sequences were provided by A. Inoue (Tohoku University). Oligonucleotides for the sgRNAs were phosphorylated and annealed for insertion into BsmBI-digested lenti-CRISPR v2 backbone (Addgene, 52961). Lentiviruses were produced by transfecting HEK293T17 cells with envelope, packaging and guide DNAs at a 1:2:3 ratio. Medium was collected at 48 and 72 h after transfection. Viral particles were concentrated by ultracentrifugation at 28,000 r.p.m. for 4 h at 4 °C. Cancer cell lines were seeded on poly(lysine)-coated six-well plates and transduced once the cells reached 70% confluence. The medium was refreshed after 48 h and cells were transduced again for 48 h. Polybrene (10 µg ml^–1^) was used to enhance transduction efficiency. Cells were selected with 1 μg ml^–1^ puromycin for 5 d.

#### Generation of Tet-inducible GNAS active mutant cell lines

The GNAS active mutant was previously generated by site-directed mutagenesis of R201 to cysteine^56^. GNAS^R201C^ cDNA was cloned into the pENTR backbone (pDONR221; ThermoFisher Scientific, 12536017) using the Gateway cloning BP reaction according to the manufacturer’s protocols (Invitrogen, 11789020). Activity of the GNAS^R201C^ active mutant was confirmed by cAMP-responsive element (CRE) luciferase assay (Dual-Glo Luciferase Assay System; Promega, E2920) or cAMP immunoassay (R&D Systems, KGE002B). The GNAS^R201C^-pENTR vector was then recombined with the lentiviral vector pLVX-TetOne FLAG Puro (provided by the Krogan group, UCSF) using the Gateway LR reaction according to the manufacturer’s protocol (Invitrogen, 11791020). Viral particles were collected from HEK293T17 cells and concentrated by ultracentrifugation. Cells were transduced two times for 48 h and then selected with 1 μg ml^–1^ puromycin for 5 d. Expression of GNAS^R201C^ was induced by adding 1 μg ml^–1^ doxycycline.

### Cell extracts

For samples to be analyzed with the HT-KAM platform, cells at ~85% confluence were washed three times with cold PBS and lysed with freshly prepared 1× cell lysis buffer (1 ml per 2.5 × 10^6^ cells) (10× Cell Lysis Buffer; Cell Signaling, 9803) complemented with 1× Halt Protease & Phosphatase (100×; ThermoScientific, 1861281). Cell lysates were collected and spun down at 16,500*g* for 15 min at 4 °C and supernatants were stored at –80 °C. For samples to be analyzed by western blot, cell lysates were prepared with RIPA lysis buffer supplemented with protease and phosphatase inhibitors. After clearing by centrifugation at 13,400*g* for 10 min at 4 °C, lysates were analyzed as described in the respective experiments. For samples to be analyzed with HT-KAM, WiDr cells were treated as detailed in ref. ^13^.

### Kinase inhibitors and cell treatment conditions

Details on inhibitors, blocking antibody and PGE2 reagents are provided in Supplementary Table 1. Drug treatment conditions (concentration and time) related to western blots, ELISAs, kinase activity profiles and qRT–PCR are provided in Supplementary Tables 2 and 3. For western blots, cells were serum starved (0.25% FBS) for 16 h before treatment (conditions available in Supplementary Table 3). We included a demarcation line where blots were not contiguous (see Figs. 3a, 4a and 5e,g (bottom)).

### Cell viability assays

To assess the growth/survival response of cell lines to single or combinatorial drug treatments, we used the CellTiter-Glo cell viability assay (Promega, G7571). Cell culture and luminescence readouts were performed in 96- and 384-well plates after 3-day treatments. GI_50_ corresponds to the concentration of a given drug that causes 50% inhibition of cell growth after 3 d of treatment. For figure panels displaying cell survival results (that is, the shift in vemurafenib sensitivity and synergy analysis shown in Figs. 2a,b,e, 3b, 5c,f,h and 6a,b and Extended Data Figs. 2a,c and 3b), experimental conditions for all 2-fold serial dilution treatments were started at a concentration of ≥8-fold GI_50_ and ended at ≥0.125-fold the GI_50_ concentration, where all concentrations were adapted/specific to each cell line and each drug. The effects of drug combinations on cell growth were assessed by calculating the fold change in vemurafenib sensitivity and CI, which were experimentally measured from ≥9 individual data points around drugs’ GI_50_, that is, at GI_50_, 0.5×GI_50_ and 2×GI_50_ concentrations of each drug for each drug combination experiment. To address the particular effects of some drug treatments on some cell lines, the choice of experimental data points centered around drugs’ GI_50_ effects was adjusted by including individual data points ranging from ≥GI_25_ to ≤GI_75_, leading to calculation of fold change in vemurafenib sensitivity and CI from 12 to 20 individual data points, as previously explained^7^. For Fig. 5c, cells were treated with serial dilutions of PGE2 starting at 128 pg ml^–1^ every 12 h over 3 d.

To calculate the CI values in Figs. 2a,b,e, 5c,f,h and 6a, we applied the Bliss independence model^57,58^, which uses experimental profiles and avoids inaccuracies that commonly occur with dose–effect curve estimation approaches. CI is calculated as CI = –log_2_(*E*_AB_/(*E*_A_ × *E*_B_)), where *E*_A_ and *E*_B_ correspond to the effects of drugs A and B alone at a given concentration and *E*_AB_ corresponds to the combined effects of drugs A and B at these same concentrations. In this model, CI > 0 indicates synergistic effect, CI = 0 indicates additive effect and CI < 0 indicates antagonistic effect.

To compare the effects of triple versus dual drug combinations in Fig. 3b, we calculated CI as follows: CI = –log_2_(*E*_ABC_/(*E*_A_ × *E*_B_)), where *E*_A_ and *E*_B_ correspond to the effects of drugs A/vemurafenib and B/gefitinib alone at a given concentration and *E*_ABC_ corresponds to the combined effects of drugs A and B at these same concentrations combined with a third drug (C/dasatinib) at the cell line-specific GI_50_ of dasatinib. The same method was applied to analyze the data shown in Fig. 6b.

### Colony formation assays

Colony formation assays were performed as previously described in ref. ^13^. In brief, to test the responses of CRC cells to different treatments, cells were plated in medium containing 10% FBS 24 h before being washed with serum-free medium and cultured for 24 h in medium containing 0.1% serum. After low-serum incubation, cells were treated with drugs for 30 min and stimulated with 10% FBS.

### Kinase activity mapping assay

The HT-KAM platform uses arrays of peptides that act as sensors of phosphorylation activity^7^. The phospho-catalytic signature of samples is established from simultaneously occurring ATP consumption tests measured in the presence of individual peptides that are experimentally isolated from each other. Assays were run in 384-well plates, where each experimental well contained one peptide. The final reaction mixtures in each well contained (1) kinase assay buffer (Cell Signaling, 9802), (2) 250 nM ATP (Cell Signaling, 9804), (3) 200 µg ml^–1^ of 11-mer peptide and (4) samples generated from cells with ~10 µg ml^–1^ total protein extract. All reagents were kept on ice and plates were placed on cold blocks until enzymatic reactions were started. Once dispensing of the reaction mixtures was complete, the plates were incubated for 1 h at 30 °C. ATP was detected using Kinase-Glo reagent (Promega, V3772), which stops the activity of kinases and produces a luminescence signal that directly correlates with the amount of remaining ATP in samples. Luminescence was acquired using the Synergy 2 Multi-Mode Microplate Reader from BioTek. For a more detailed description of the peptide sensor design, sequences and connectivity between peptides and kinases, as well as data normalization steps and analysis, refer to refs. ^7,9,10,59,60^. The activity of kinase enzymes was derived from their respective subset of biological peptide targets included in the assay.

### Antibodies and western blotting

For western blotting, samples were denatured by boiling in 1× Laemmli buffer and run on an 8% SDS–PAGE gel. Membranes were incubated with primary antibodies overnight at 4 °C, washed three times with TBST, incubated with secondary antibodies for 1 h at room temperature and developed using chemoluminescence (Pierce, 32209). Details on the antibodies used are provided in Supplementary Table 4.

### ELISA

To detect secreted PGE2, conditioned medium from BRAF^V600E^ CRC cell lines treated with vemurafenib ± gefitinib was collected. Particulates were removed by centrifugation, and samples were processed according to the manufacturer’s protocol (R&D Systems, KGE004B).

### RNA extraction, cDNA synthesis and qRT–PCR

RNA was purified using TRIzol followed by digestion with RNase-free DNase (Qiagen). cDNA synthesis was performed using the High-Capacity cDNA Reverse Transcription kit (ABI). Primer efficiencies were assessed by serial dilution. qRT–PCR reactions were performed in the QuantStudio 5 Real-Time PCR System using SYBR-Green Power master mix (ABI) with default cycling conditions; results were analyzed with QuantStudio 5 analysis software. All mRNA levels were assayed in quadruplicate; dissociation curves were checked and products were run in agarose gels to confirm amplification of only one product. Relative mRNA levels of β-catenin target genes (*MYC*, *AXIN2*, *ASCL2*, *S100A6*, *LEF1*, *NOTCH2*, *SP5*) were calculated by the 2^(–∆∆Ct)^ method using *ACTB* and *UBC* as controls. The sequences (5′→3′) of the primers we used to measure the mRNA levels of β-catenin target and housekeeping genes are provided in Supplementary Table 5. For statistical analysis of qRT–PCR results, we used a two-sample equal-variance *t* test (with a two-tailed distribution) to determine the significance of differences in gene expression.

### Automated IHC procedure

To identify changes in protein expression in tumors from PDXs treated or not with dabrafenib and/or trametinib (Figs. 1e and 5i and Extended Data Fig. 1b) and tumors from patients (Extended Data Figs. 1d and 5c), IHC staining was preformed using the Ventana DISCOVERY ULTRA autostainer system hosted at the UCSF Histology and Biomarker Core. A critical advantage of the fully automated DISCOVERY ULTRA pipeline is that, once an IHC protocol is set, all parameters and workflows are automated and repeated identically, thus allowing for all biospecimens to be processed in the exact same way, which further allows for automated image processing and comparative analysis.

In brief, slides where sectioned at 4-µm thickness, mounted on positively charged slides and air dried. To increase tissue adhesion, slides were baked in an oven at 60 °C for a minimum of 1 h to a maximum of 24 h. Deparaffinization was performed on the DISCOVERY ULTRA system in three cycles of 8 min each in EZ Prep solution warmed to 72 °C. Antigen retrieval was performed at high temperature, between 95 °C and 100 °C, in Cell Conditioning 1 Solution for 4 to 92 min as required by the tissue and antibody combination. Before primary antibody application, inhibitor (specifically Inhibitor CM from the DAB kit) was applied and slides were incubated for between 8 and 20 min. Primary antibody was diluted in Discovery Ab Diluent. Species-specific secondary antibody, either OmniMAP or HQ and enzyme conjugate, was applied and slides were incubated with low heat between 36 and 37 °C. DAB from the DISCOVERY ChromoMap DAB RUO kit was selected as the chromogenic detector for which the DISCOVERY UTLRA system hard codes the incubation settings. Hematoxylin nuclear counterstain (760-2021) was applied for 4 min followed by Bluing reagent for an additional 4 min. Slides were washed and dehydrated according to the Ventana standard operating procedure and were coverslipped using 0.17-mm-thick glass coverslips and Cytoseal XYL mounting medium (Richard-Allan Scientific, 22050262).

To detect SRC phosphorylated at Y419, phospho-Src (Y419) EGFR rabbit polyclonal antibody supplied by R&D Systems (AF2685) was used at a dilution of 1:50. Cell conditioning in CC1 was performed at 95 °C for 64 min. Inhibitor was applied for 16 min. Slides were incubated with the primary antibody at 37 °C for 32 min. Anti-rabbit HQ was used as the secondary antibody and slides were incubated at 37 °C for 16 min followed by anti-HQ–HRP enzyme conjugate, which was applied for 16 min. DAB was used as the chromogenic detector with hematoxylin selected for the counterstain.

To detect total SRC, total SRC (36D1) rabbit monoclonal antibody manufactured by Cell Signaling Technology (2109) was used at a dilution of 1:800. Cell conditioning in CC1 was performed at 95 °C for 32 min. Inhibitor was applied for 16 min. Slides were incubated with the primary antibody at 37 °C for 32 min. OmniMAP anti-rabbit was used as the secondary antibody and slides were incubated at 37 °C for 12 min. DAB was used as the chromogenic detector with hematoxylin selected for the counterstain.

To detect COX2, COX2 (SP21) rabbit monoclonal antibody manufactured by Abcam (ab16708) was used at a dilution of 1:100. Cell conditioning in CC1 was performed at 95 °C for 64 min. Inhibitor was applied for 16 min. Slides were incubated with the primary antibody at 37 °C for 32 min. OmniMAP anti-rabbit was used as the secondary antibody and slides were incubated at 37 °C for 8 min. DAB was used as the chromogenic detector with hematoxylin selected for the counterstain.

### Automated processing and analysis of IHC images

IHC images were processed with inForm Tissue Finder software (Akoya Biosciences). The feature recognition algorithms available in inForm automate the detection and segmentation of tissues and cells, as well as the quantification of immunostaining intensities (Figs. 1f and 5j and Extended Data Fig. 1c). Automation provides consistent, reproducible results and enables comparative studies across specimens, samples and images.

The image processing workflow is a machine learning process defined by, and adjusted through, an iterative sequence of user-trained modules and variables. The spectral components of each image are first unmixed using a spectral library. Next, all cells composing the immunostained tissue image are detected and annotated using tissue and cell segmentation modules. These steps locate individual cellular objects by identifying nuclei from the spectrum corresponding to the hematoxylin stain. On the basis of nucleus boundaries and definable cytoplasmic/cell membrane features, the inForm software finds and draws the boundaries of each cell. This enables systematic quantification of immunostaining intensity for each individual cell composing the tissue and across the different tissue types (for example, tumor versus stroma). The pattern recognition detection/segmentation and the immunostaining intensity scoring of tissues and cells are initially run on 3–5 images and then reiterated using an additional set of 15–25 randomly chosen images to further fine-tune all parameters and validate the consistency of processing across images and visualization output. Once the ‘training’ of the inForm algorithm is considered final, batch processing of all images is applied, allowing for systematic processing with identical parameters across hundreds of images and generation of results that are fully comparable among all individual cells from all tissues and biospecimens.

To quantify protein expression at the single-cell level in tumor areas, we automated the scoring of immunostaining intensities using a binning tool available in the inForm software. The same binning thresholds were used for each antibody (for example, the same four-bin intensity levels across treatments and across PDXs for SRC (total) to provide comparable results between conditions and tumor cases). However, the thresholds were specifically adapted to each antibody (that is, different four-bin intensity levels for SRC versus SRC pY419 versus COX2).

### PDX models and BRAF^V600E^ CRC cell line xenograft studies

PDX models were established from the tumor biopsy samples of consenting UCSF patients as previously described (UCSF Institutional Review Board, 12-09139)^4,11^. All patients went on to receive dabrafenib + trametinib as part of a clinical trial^4^. JAX NOD-scid-gamma mice bearing subcutaneous PDXs were randomized into vehicle or treatment groups when tumor volumes reached 100–150 mm^3^ with rolling enrollment. Six- to 9-week-old female mice were treated with vehicle (0.1% Tween-20 or 0.5% hydroxypropyl methylcellulose and 0.2% Tween-80) or targeted therapies for 21 d (Figs. 3d,e and 6d) or up to 60 d (Fig. 7a,b) in the UCSF Preclinical Therapeutics Core (San Francisco, CA).

Inhibitors administered by oral gavage were purchased from Selleck Chemicals and dosed daily as follows: celecoxib 50 mg kg^–1^ by mouth daily; encorafenib 20 mg kg^–1^ by mouth daily (see ref. ^61^); dabrafenib 30 mg kg^–1^ by mouth daily; dasatinib 20 mg kg^–1^ by mouth daily; gefitinib 50 mg kg^–1^ by mouth daily; saracatinib 25 mg kg^–1^ by mouth daily; trametinib 0.6 mg kg^–1^ by mouth daily; vemurafenib 50 mg kg^–1^ by mouth daily. Panitumumab (used in Fig. 7a,b) was provided by Amgen Oncology and was administered at 200 µg by intraperitoneal injection twice weekly. For WiDr and KM20 cell line xenograft studies (Fig. 3d), 8 and 7 mice per treatment group, respectively, were dosed daily with a combination of the following inhibitors: PLX4702 (50 mg kg^–1^ per day orally), gefitinib (50 mg kg^–1^ per day orally), dasatinib (50 mg kg^–1^ per day orally) and saracatinib (25 mg kg^–1^ per day orally).

Mice were monitored for signs of toxicity (for example, weight loss), and tumor size was evaluated twice a week with digital caliper measurements. The 15% body weight reduction threshold for holding drug was not met. The same procedures were followed for cell line-derived xenograft models. The maximum tumor size permitted by the LARC and IACUC is 2,000 mm^3^; this maximum tumor size was not exceeded.

To test the significance of the changes in tumor volume over time between treatment arms and vehicle in Figs. 3d–i, 6d,f,g and 7a,b, we used the following statistical tests: Student’s *t* test, GLM, GLM *P* values corrected for FDR. Besides plotting the relative tumor volume in Figs. 3d,e, 6d and 7a,b, we also calculated effect size measured as the GLM standard coefficient. A GLM was applied to each tumor model separately or combined. In Fig. 7b, none of the mice treated with encorafenib alone were available at the last two time points (these mice were killed because of tumor size), which is why no *P* value could be calculated (represented as an ‘X’ in the table underneath the plot). All raw and relative tumor volumes (and the number of mice per group) can be found in the Source Data.

### Patient specimens

Tumor specimens were used in research following patient consent and approval by the UCSF Institutional Review Board (12-09139).

### Statistics and reproducibility

We provide general information on how statistical analyses of data were conducted and general information on the reproducibility of experiments. We used XLS (versions 14.0 and 16.0), R (version 4.0.2), Rstudio (version 1.1.463), Prism (version 6.0e), MATLAB (version 9.6), QuantStudio (version 5) and inForm (version 2.0) to collect and analyze data. No data were excluded from analyses. No statistical method was used to predetermine sample size, but our sample sizes are similar to those reported in previous publications^6,7,12–14^. Data distribution was assumed to be normal, but this was not always formally tested. Data collection and analysis were not performed with blinding to the conditions of the experiments.

For the analysis leading to the heatmap in Fig. 1a, the average value of ATP consumption in sample-containing wells measured across 228 peptides and 14 peptide-free controls was used for internal normalization for each experimental run (that is, the mean-centering value established from 242 data points/wells per 384-well plate; previously explained in refs. ^7,9,10^). Other normalization schemes were used for further analysis and cross-validation (that is, (1) the subset of 14 peptide-free control wells (that is, cell extract alone), (2) the subset of 16 Y/S/T-free peptides or (3) the subset of 63 reference peptides). The activity for each peptide was then calculated as the difference in ATP consumption between each peptide and the internal mean. ATP consumption measurements associated with each peptide were then averaged across all biological and technical replicates. Next, phosphorylation activity profiles across all 228 individual peptides were compared between treated (vemurafenib or vemurafenib + gefitinib or vemurafenib + cetuximab) and control (UNT) (calculated as the difference in ATP consumption). Finally, phospho-catalytic activity signatures measured across the 228-peptide sensors were subjected to unsupervised hierarchical clustering. Phospho-catalytic activities are color coded according to the relative level of activity measured in the presence of each peptide for each treatment.

For the analysis to generate the heatmaps in Fig. 1b (and related plots in Fig. 1c,d), the activity of kinases was calculated as the average of the phosphorylation activities measured in the presence of their respective biological peptide subsets (that is, derived from values/calculations used to generate Fig. 1a). We systematically converted peptide phosphorylation profiles into kinase activity signatures for individual kinases and kinase families that were detected with ≥3 distinct biological peptide sensors.

In Figs. 3d,e and 7c,d, changes in tumor volume at each time point are shown as the mean volume of all tumors per treatment arm (± standard error). *P* values from Student’s *t* tests comparing treatment versus vehicle, or between drug treatment arms, are shown.

In Fig. 6d, changes in tumor volume converted to cumulative tumor volume are shown for all individual tumors per treatment arm at day 21 (the final time point to assess and compare the efficacy of different drug combinations). Significance when comparing treatment versus vehicle at day 21 was assessed, and *P* values (Student’s *t* test) are shown in the plot.

In Figs. 3f–i, 6f,g and 7c,d, we used a GLM to analyze profiles of tumor growth. We first zero-normalized the data compared to control vehicle for each model and for time point. We then ran a GLM (available in R) on the normalized data to test the association of change in tumor volume between treatment and vehicle across all time points. The effect size of each treatment compared to vehicle was calculated (that is, GLM standard coefficient) along with significance (that is, FDR-corrected GLM *P* value). This GLM approach and data normalization also allowed us to compare the effects of different treatments (for example, with or without dasatinib (Fig. 3h,i); with or without celecoxib (Figs. 6g and 7d)), as well as to combine the distinct models (for example, all cell line tumor xenografts (Fig. 3f,h) or all PDXs (Figs. 3g,i, 6f,g and 7c,d)) to measure the overall effect of drug combinations and their significance.

For Figs. 2d, 3a, 4a and 5b,d,e,g and Extended Data Figs. 1a, 2b, 4c and 5a,b, each experiment was repeated independently at least twice with similar results.

Other statistical and predictive methods to compare sample groups and reproducibility between signatures included unsupervised or semisupervised hierarchical clustering using Euclidean distance or (absolute) correlation (centered or uncentered) and Ward linkage or complete or average linkage to group phospho-activity signatures on the basis of their similarities or differences; Student’s *t* tests with or without Benjamini–Hochberg FDR correction; Wilcoxon rank-sum tests with or without FDR correction (*P* < 0.05); and GLMs.

### Reporting summary

Further information on research design is available in the Nature Portfolio Reporting Summary linked to this article.

## Supplementary information

Reporting Summary

Supplementary Tables Reagents and experimental conditions.

## Data Availability

Data supporting the findings of this study are available from the corresponding author on reasonable request. PhosphoAtlas is available at https://cancer.ucsf.edu/phosphoatlas. Source data are provided with this paper.

## Source data

Source Data Figs.1–7 Statistical source data.
Source Data Figs. 2–5 and Extended Data Figs. 1, 2, 4 and 5 Supplementary western blot images.

## References

[1] Venderbosch S (2014). Mismatch repair status and *BRAF* mutation status in metastatic colorectal cancer patients: a pooled analysis of the CAIRO, CAIRO2, COIN, and FOCUS studies. Clin. Cancer Res..

[2] Morris V (2014). Progression-free survival remains poor over sequential lines of systemic therapy in patients with *BRAF*-mutated colorectal cancer. Clin. Colorectal Cancer.

[3] Kopetz S (2015). Phase II pilot study of vemurafenib in patients with metastatic *BRAF*-mutated colorectal cancer. J. Clin. Oncol..

[4] Corcoran RB (2015). Combined BRAF and MEK inhibition with dabrafenib and trametinib in BRAF^V600^-mutant colorectal cancer. J. Clin. Oncol..

[5] Kopetz S (2019). Encorafenib, binimetinib, and cetuximab in BRAF^V600E^-mutated colorectal cancer. N. Engl. J. Med..

[6] Corcoran RB (2018). Combined BRAF, EGFR, and MEK inhibition in patients with BRAF^V600E^-mutant colorectal cancer. Cancer Discov..

[7] Coppe JP (2019). Mapping phospho-catalytic dependencies of therapy-resistant tumours reveals actionable vulnerabilities. Nat. Cell Biol..

[8] Olow A (2016). An atlas of the human kinome reveals the mutational landscape underlying dysregulated phosphorylation cascades in cancer. Cancer Res..

[9] Mori, M., Pan, B. & Coppé, J.-P. High-throughput kinase activiy mapping (HT-KAM) system: biochemical assay. *Nat. Protoc. Exch.*10.1038/protex.2019.029 (2019).

[10] Yau, C., Wolf, D. M. & Coppé, J. High-throughput kinase activity mapping (HT-KAM) system: analysis of phospho-catalytic profiles. *Nat. Protoc. Exch.*10.1038/protex.2019.030 (2019).

[11] Rajaram S (2019). A multi-modal data resource for investigating topographic heterogeneity in patient-derived xenograft tumors. Sci. Data..

[12] Corcoran RB (2012). EGFR-mediated re-activation of MAPK signaling contributes to insensitivity of *BRAF* mutant colorectal cancers to RAF inhibition with vemurafenib. Cancer Discov..

[13] Prahallad A (2012). Unresponsiveness of colon cancer to BRAF^V600E^ inhibition through feedback activation of EGFR. Nature.

[14] Yang H (2012). Antitumor activity of BRAF inhibitor vemurafenib in preclinical models of *BRAF*-mutant colorectal cancer. Cancer Res..

[15] Manning G, Whyte DB, Martinez R, Hunter T, Sudarsanam S (2002). The protein kinase complement of the human genome. Science.

[16] Erpel T, Courtneidge SA (1995). Src family protein tyrosine kinases and cellular signal transduction pathways. Curr. Opin. Cell Biol..

[17] Parsons SJ, Parsons JT (2004). Src family kinases, key regulators of signal transduction. Oncogene.

[18] Thomas SM, Brugge JS (1997). Cellular functions regulated by Src family kinases. Annu. Rev. Cell Dev. Biol..

[19] Xi Y (2013). Identification of pY654-β-catenin as a critical co-factor in hypoxia-inducible factor-1α signaling and tumor responses to hypoxia. Oncogene.

[20] Herbst A (2014). Comprehensive analysis of β-catenin target genes in colorectal carcinoma cell lines with deregulated Wnt/β-catenin signaling. BMC Genomics.

[21] Pai R (2002). Prostaglandin E_2_ transactivates EGF receptor: a novel mechanism for promoting colon cancer growth and gastrointestinal hypertrophy. Nat. Med..

[22] Buchanan FG (2006). Role of β-arrestin 1 in the metastatic progression of colorectal cancer. Proc. Natl Acad. Sci. USA.

[23] Castellone MD, Teramoto H, Williams BO, Druey KM, Gutkind JS (2005). Prostaglandin E_2_ promotes colon cancer cell growth through a G_s_–axin–β-catenin signaling axis. Science.

[24] Dorsam RT, Gutkind JS (2007). G-protein-coupled receptors and cancer. Nat. Rev. Cancer.

[25] Wang D, Dubois RN (2010). Eicosanoids and cancer. Nat. Rev. Cancer.

[26] Simmons DL, Botting RM, Hla T (2004). Cyclooxygenase isozymes: the biology of prostaglandin synthesis and inhibition. Pharmacol. Rev..

[27] Wang D, Dubois RN (2010). The role of COX-2 in intestinal inflammation and colorectal cancer. Oncogene.

[28] Montero-Conde C (2013). Relief of feedback inhibition of HER3 transcription by RAF and MEK inhibitors attenuates their antitumor effects in *BRAF*-mutant thyroid carcinomas. Cancer Discov..

[29] Petricoin EF, Zoon KC, Kohn EC, Barrett JC, Liotta LA (2002). Clinical proteomics: translating benchside promise into bedside reality. Nat. Rev. Drug Discov..

[30] Blume-Jensen P, Hunter T (2001). Oncogenic kinase signalling. Nature.

[31] Fleuren ED, Zhang L, Wu J, Daly RJ (2016). The kinome ‘at large’ in cancer. Nat. Rev. Cancer.

[32] Konieczkowski DJ, Johannessen CM, Garraway LA (2018). A convergence-based framework for cancer drug resistance. Cancer Cell.

[33] Roskoski R (2015). Src protein-tyrosine kinase structure, mechanism, and small molecule inhibitors. Pharmacol. Res..

[34] Girotti MR (2015). Inhibitors that also target SRC are effective in drug-resistant *BRAF* mutant melanoma. Cancer Cell.

[35] Girotti MR (2013). Inhibiting EGF receptor or SRC family kinase signaling overcomes BRAF inhibitor resistance in melanoma. Cancer Discov..

[36] Kopetz S (2007). Targeting SRC and epidermal growth factor receptor in colorectal cancer: rationale and progress into the clinic. Gastrointest. Cancer Res..

[37] Fabian JR, Daar IO, Morrison DK (1993). Critical tyrosine residues regulate the enzymatic and biological activity of Raf-1 kinase. Mol. Cell. Biol..

[38] Marais R, Light Y, Paterson HF, Marshall CJ (1995). Ras recruits Raf-1 to the plasma membrane for activation by tyrosine phosphorylation. EMBO J..

[39] Marais R, Light Y, Paterson HF, Mason CS, Marshall CJ (1997). Differential regulation of Raf-1, A-Raf, and B-Raf by oncogenic *ras* and tyrosine kinases. J. Biol. Chem..

[40] Hu J (2013). Allosteric activation of functionally asymmetric RAF kinase dimers. Cell.

[41] Chen R (2001). Regulation of Akt/PKB activation by tyrosine phosphorylation. J. Biol. Chem..

[42] Coluccia AM (2006). SKI-606 decreases growth and motility of colorectal cancer cells by preventing pp60^c-Src^-dependent tyrosine phosphorylation of β-catenin and its nuclear signaling. Cancer Res..

[43] Brembeck FH, Rosario M, Birchmeier W (2006). Balancing cell adhesion and Wnt signaling, the key role of β-catenin. Curr. Opin. Genet. Dev..

[44] Silva CM (2004). Role of STATs as downstream signal transducers in Src family kinase-mediated tumorigenesis. Oncogene.

[45] Cancer Genome Atlas Network. Comprehensive molecular characterization of human colon and rectal cancer. *Nature* **487**, 330–337 (2012). 10.1038/nature11252 PMC3401966 22810696

[46] Anastas JN, Moon RT (2013). WNT signalling pathways as therapeutic targets in cancer. Nat. Rev. Cancer.

[47] Kosumi K (2019). Prognostic association of PTGS2 (COX-2) over-expression according to *BRAF* mutation status in colorectal cancer: results from two prospective cohorts and CALGB 89803 (Alliance) trial. Eur. J. Cancer.

[48] Sharma MR (2012). Dasatinib in previously treated metastatic colorectal cancer: a phase II trial of the University of Chicago Phase II Consortium. Invest. New Drugs.

[49] Parseghian CM (2017). Dual inhibition of EGFR and c-Src by cetuximab and dasatinib combined with FOLFOX chemotherapy in patients with metastatic colorectal cancer. Clin. Cancer Res..

[50] Reddy SM (2015). Phase II study of saracatinib (AZD0530) in patients with previously treated metastatic colorectal cancer. Invest. New Drugs.

[51] Chen EY (2018). A phase II study of celecoxib with irinotecan, 5-fluorouracil, and leucovorin in patients with previously untreated advanced or metastatic colorectal cancer. Am. J. Clin. Oncol..

[52] André T (2007). Phase II study of an optimized 5-fluorouracil–oxaliplatin strategy (OPTIMOX2) with celecoxib in metastatic colorectal cancer: a GERCOR study. Ann. Oncol..

[53] Maiello E (2006). FOLFIRI with or without celecoxib in advanced colorectal cancer: a randomized phase II study of the Gruppo Oncologico dell’Italia Meridionale (GOIM). Ann. Oncol..

[54] Sanjana NE, Shalem O, Zhang F (2014). Improved vectors and genome-wide libraries for CRISPR screening. Nat. Methods.

[55] Shalem O (2014). Genome-scale CRISPR–Cas9 knockout screening in human cells. Science.

[56] Iglesias-Bartolome R (2015). Inactivation of a Gα_s_–PKA tumour suppressor pathway in skin stem cells initiates basal-cell carcinogenesis. Nat. Cell Biol..

[57] Foucquier J, Guedj M (2015). Analysis of drug combinations: current methodological landscape. Pharmacol. Res. Perspect..

[58] Berenbaum MC (1989). What is synergy?. Pharmacol. Rev..

[59] Pan, B. et al. Functional detection and inhibition of the targetable oncogenic kinome of chemotherapy-treated triple negative breast cancer cells. *Cancer Res.*10.1158/1538-7445.SABCS15-P6-08-03 (2016).

[60] Mori, M. et al. Kinase-sensing system to identify the oncogenic phospho-fingerprint of breast cancer. *J. Clin. Oncol.*10.1200/jco.2013.31.26_suppl.23 (2013).

[61] Krepler C (2016). Personalized preclinical trials in BRAF inhibitor-resistant patient-derived xenograft models identify second-line combination therapies. Clin. Cancer Res..

