# Supplementary material for: A reversible SRC-relayed COX2 inflammatory program drives resistance to BRAF and EGFR inhibition in BRAFV600E colorectal tumors
Source: Nat Cancer. 2023 Feb 9;4(2):240–56. doi: 10.1038/s43018-022-00508-5 (PMC9970872; doi:10.1038/s43018-022-00508-5)
Supplement: Supplementary file 1 — Reporting Summary [file 43018_2022_508_MOESM1_ESM.pdf]

nature portfolio

Corresponding author(s): Jean-Philippe Coppé

Last updated by author(s): Dec 8, 2022

Reporting Summary

Nature Portfolio wishes to improve the reproducibility of the work that we publish. This form provides structure for consistency and transparency in reporting. For further information on Nature Portfolio policies, see our Editorial Policies and the Editorial Policy Checklist.

Statistics

For all statistical analyses, confirm that the following items are present in the figure legend, table legend, main text, or Methods section.

n/a	Confirmed	
☐	☒	The exact sample size (*n*) for each experimental group/condition, given as a discrete number and unit of measurement
☐	☒	A statement on whether measurements were taken from distinct samples or whether the same sample was measured repeatedly
☐	☒	The statistical test(s) used AND whether they are one- or two-sided<br>*Only common tests should be described solely by name; describe more complex techniques in the Methods section.*
☐	☒	A description of all covariates tested
☐	☒	A description of any assumptions or corrections, such as tests of normality and adjustment for multiple comparisons
☐	☒	A full description of the statistical parameters including central tendency (e.g. means) or other basic estimates (e.g. regression coefficient) AND variation (e.g. standard deviation) or associated estimates of uncertainty (e.g. confidence intervals)
☐	☒	For null hypothesis testing, the test statistic (e.g. *F*, *t*, *r*) with confidence intervals, effect sizes, degrees of freedom and *P* value noted<br>*Give P values as exact values whenever suitable.*
☒	☐	For Bayesian analysis, information on the choice of priors and Markov chain Monte Carlo settings
☐	☒	For hierarchical and complex designs, identification of the appropriate level for tests and full reporting of outcomes
☐	☒	Estimates of effect sizes (e.g. Cohen's *d*, Pearson's *r*), indicating how they were calculated

Our web collection on statistics for biologists contains articles on many of the points above.

Software and code

Policy information about availability of computer code

Data collection	XLS, TXT, CSV
Data analysis	XLS (versions 14.0 and 16.0), R (version 4.0.2), Rstudio (version 1.1.463), Prism (version 6.0e), MATLAB (version 9.6), QuantStudio™ (version 5), inForm (version 2.0)

For manuscripts utilizing custom algorithms or software that are central to the research but not yet described in published literature, software must be made available to editors and reviewers. We strongly encourage code deposition in a community repository (e.g. GitHub). See the Nature Portfolio guidelines for submitting code & software for further information.

Data

Policy information about availability of data

All manuscripts must include a data availability statement. This statement should provide the following information, where applicable:

- Accession codes, unique identifiers, or web links for publicly available datasets
- A description of any restrictions on data availability
- For clinical datasets or third party data, please ensure that the statement adheres to our policy

Data supporting the findings of this study are available from the corresponding author on reasonable request. PhosphoAtlas is available at https://cancer.ucsf.edu/phosphoatlas.

March 2021

Human research participants

Policy information about studies involving human research participants and Sex and Gender in Research.

Reporting on sex and gender	This study does not involve human research participants, hence no report on sex and gender is available
Population characteristics	This study does not involve human research participants, hence no population characteristics is available
Recruitment	This study does not involve human research participants, hence no recruitment is available
Ethics oversight	This study does not involve human research participants, hence no ethics committee oversight is available

Note that full information on the approval of the study protocol must also be provided in the manuscript.

Field-specific reporting

Please select the one below that is the best fit for your research. If you are not sure, read the appropriate sections before making your selection.

☒ Life sciences ☐ Behavioural & social sciences ☐ Ecological, evolutionary & environmental sciences

For a reference copy of the document with all sections, see nature.com/documents/nr-reporting-summary-flat.pdf

Life sciences study design

All studies must disclose on these points even when the disclosure is negative.

Sample size	Sample size was defined by technical replicates and biological replicates. Sample sizes were sufficient based on the confidence of the data output of computational methods we applied.
Data exclusions	No data were excluded.
Replication	Unsupervised and Supervised Clustering, Principal Component Analysis, FDR-corrected t-test and Wilcoxon rank sum test, averages/median and SD, and general linear model (GLM), were systematically used to compare samples. All independently repeated experiments generated successfully replicated results and allowed cross-validation/replication between experiments and within experiments. Biological replicates: n≥2; technical replicates: n≥2.
Randomization	Samples were grouped based on the characteristics of cell lines used in the assay that was analyzed, to minimize confounding factors. For example, patient-derived xenografts were grouped, or cell-line derived xenograft mouse models were grouped.
Blinding	Owing computational/statistical analyses and how samples were generated, all investigators were effectively blinded to group allocation during data collection and analysis.

Reporting for specific materials, systems and methods

We require information from authors about some types of materials, experimental systems and methods used in many studies. Here, indicate whether each material, system or method listed is relevant to your study. If you are not sure if a list item applies to your research, read the appropriate section before selecting a response.

Materials & experimental systems

n/a	Involved in the study	
☐	☒	Antibodies
☐	☒	Eukaryotic cell lines
☒	☐	Palaeontology and archaeology
☐	☒	Animals and other organisms
☒	☐	Clinical data
☒	☐	Dual use research of concern

Methods

n/a	Involved in the study	
☒	☐	ChIP-seq
☒	☐	Flow cytometry
☒	☐	MRI-based neuroimaging

Antibodies

Antibodies used	Antibodies anti-HSP90 (H-114, cat# 7947) and anti-phospho-beta-catenin (CTNNB1 Y654, cat# 57533) were procured from Santa Cruz Biotechnologies. Anti-phospho Src (Y419) (cat#AF2685) used for IHC was obtained from R&D Systems. Anti-phospho Src Family

(Y416) (D49G4, cat# 6943) used for western-blots, anti-non-phospho-Src (Y527) (cat# 2107); anti-Src (32G6, cat# 2123) used for western-blots and anti-Src (cat#2109) used for IHC, anti-phospho-p44/42 MAPK (ERK1/2 T202/204, cat# 9101); anti-p44/42 MAPK (ERK1/2, cat# 9102), anti-phospho-MAP2K1/2 (MEK1/2 T202/204, cat# 9121); anti- MAP2K1/2 (MEK1/2, cat# 9122) and anti-CSK (C74C1, cat# 4980) were from Cell Signaling. Anti-Gsalpha-Subunit (GNAS, cat# 371732) was obtained from CalBiochem (now Millipore/Sigma), anti-Gs alpha subunit, C-terminal (385-394) antibody was purchased from Calbiotech (cat# 371732), anti-COX2 was purchased from Spring Bioscience (cat# M3210) and Abcam (cat# ab16708, rabbit monoclonal antibody, clone SP21), anti-beta-catenin was purchased from BD Transduction Laboratories (CTNNB1, cat# 610153). Secondary antibodies Horseradish peroxidase (HRP)-conjugated were from GE Healthcare (rabbit cat# LNA934). We followed dilutions indicated by the manufacturers.

Validation	The validation of each primary antibody for the species and application is available from manufacturers (statements on manufacturers' website) as well as from the results provided in our study, which confirm and are confirmed by our kinase activity profiling system.

Eukaryotic cell lines

Policy information about cell lines and Sex and Gender in Research

Cell line source(s)	Cell lines A375, Sk-Mel-28, HCT116, LoVo, HEK293T17 were purchased from ATCC. Cell lines WiDr, SNUC5, HT29, Colo-205, RKO-1, LIM2405, KM20, LS411N, VACO432, SW1417, OUMS23, Mel888 were provided by Dr. R. Bernards' laboratory (also originally purchased from ATCC).
Authentication	The cell lines were not authenticated.
Mycoplasma contamination	The cell lines were not tested for mycoplasma contamination.
Commonly misidentified lines (See ICLAC register)	No cell lines used in this study were found in the database of commonly misidentified cell lines that is maintained by ICLAC and NCBI Biosample

Animals and other research organisms

Policy information about studies involving animals; ARRIVE guidelines recommended for reporting animal research, and Sex and Gender in Research

Laboratory animals	6-9 week old, female, NOD SCID gamma mice
Wild animals	The study did not involve wild animals.
Reporting on sex	Findings don't apply to only one sex. Sex was not considered in the study design. BRAF(V600E) mCRC impacts both women and men.
Field-collected samples	The study did not involve samples collected from the field.
Ethics oversight	The University of California San Francisco (UCSF) Institutional Animal Care and Use Committee (IACUC) approved the study protocol. UCSF Preclinical Therapeutics Core (PTC) Laboratory and Laboratory Animal Resource Center (LARC) operate under the Institutional Animal Care and Use Committee (IACUC), approval number: AN194778-01. Mice were monitored for signs of toxicity (e.g. weight loss) and tumor size was evaluated twice per week by digital caliper measurements. The 15% body weight reduction threshold for holding drug was not met. The same procedures were followed for cell-line derived xenograft models. Maximal tumor size permitted by LARC and IACUC is 2,000 mm3, and this maximal tumor size was not exceeded.

Note that full information on the approval of the study protocol must also be provided in the manuscript.
