# Supplementary material for: A reversible SRC-relayed COX2 inflammatory program drives resistance to BRAF and EGFR inhibition in BRAFV600E colorectal tumors
Source: Nat Cancer. 2023 Feb 9;4(2):240–56. doi: 10.1038/s43018-022-00508-5 (PMC9970872; doi:10.1038/s43018-022-00508-5)
Supplement: Source Data Figs. 2–5 and Extended Data Figs. 1, 2, 4 and 5 — Supplementary western blot images. [file 43018_2022_508_MOESM4_ESM.pdf]

A reversible SRC-relayed COX2-inflammatory program drives resistance to BRAF and EGFR inhibition in $BRAF^{V600E}$ colorectal tumors

Ana Ruiz-Saenz[1,2,§], Chloe E. Atreya[1,§], Changjun Wang[1,#,§], Bo Pan[1,#,§], Courtney A. Dreyer[1], Diede Brunen[3], Anirudh Prahallad[3], Denise P. Muñoz[1], Dana J. Ramms[4,5], Valeria Burghi[4,5], Danislav S. Spassov[1], Eleanor Fewings[1,#], Yeonjoo C. Hwang[1], Cynthia Cowdrey[1], Christina Moelders[1], Cecilia Schwarzer[1], Denise M. Wolf[1], Byron Hann[1], Scott R. VandenBerg[1], Kevan Shokat[1], Mark M. Moasser[1], René Bernards[3], J. Silvio Gutkind[4,5], Laura J. van 't Veer[1], and Jean-Philippe Coppé[1]

[1] *Helen Diller Family Comprehensive Cancer Center, University of California at San Francisco, 2340 Sutter Street, San Francisco, CA 94115, USA*
[2] *Departments of Cell Biology & Medical Oncology, Erasmus University Medical Center Rotterdam, The Netherlands*
[3] *Division of Molecular Carcinogenesis and Oncode Institute, The Netherlands Cancer Institute, Plesmanlaan 121, 1066 CX Amsterdam, The Netherlands*
[4] *Department of Pharmacology, University of California, San Diego, 9500 Gilman Drive, La Jolla, CA 92093, USA*
[5] *Moores Cancer Center, University of California, San Diego, 3855 Health Sciences Drive, La Jolla, CA 92093, USA*

[#] Current address: C.W. and B.P.: Department of Breast Surgery, Peking Union Medical College Hospital, Peking Union Medical College (PUMC) and Chinese Academy of Medical Sciences (CAMS), Beijing, China; D.S..S: Faculty of Pharmacy, Medical University of Sofia, Sofia, Bulgaria; E.F.: Institute for Computational Biomedicine, Heidelberg, Germany.

[§] Contributed equally

Supplementary File

Western blot – original images

2d

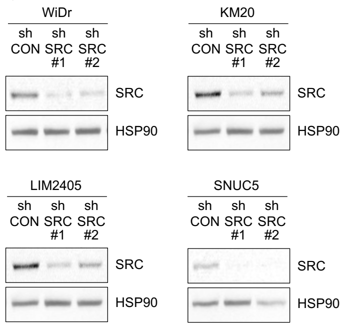

Lane # vs. cell names:

1. WiDr sh ctr
2. WiDr shSRC 3
3. WiDr shSRC 9

4. KM20 sh ctr
5. KM20 shSRC 3
6. KM20 shSRC 9

7. SNUC5 sh ctr
8. SNUC5 shSRC 3
9. SNUC5 shSRC 9

10. LIM2405 sh ctr
11. LIM2405 shSRC 3
12. LIM2405 shSRC 9

total SRC

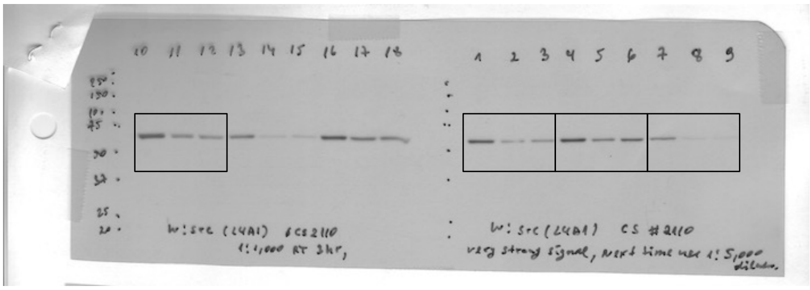

HSP90

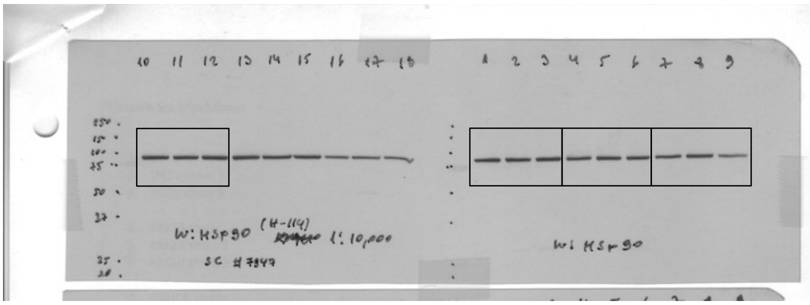

Figure 2d – western blot

WiDr and HT29 panels

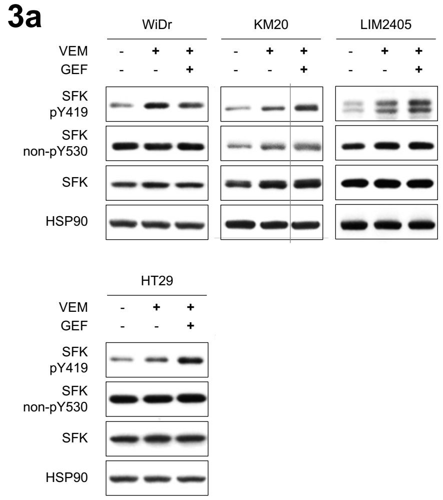

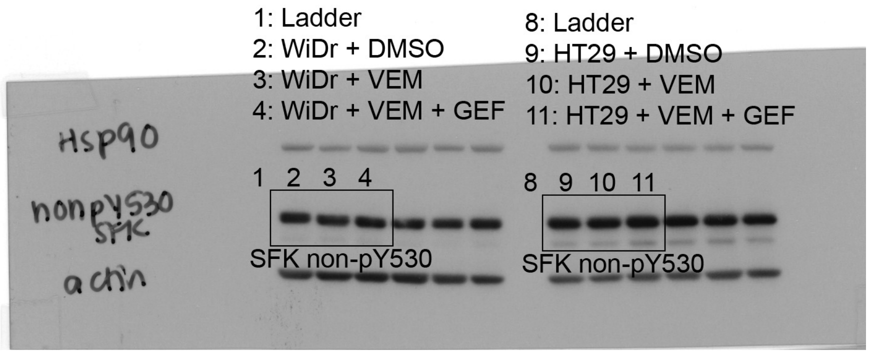

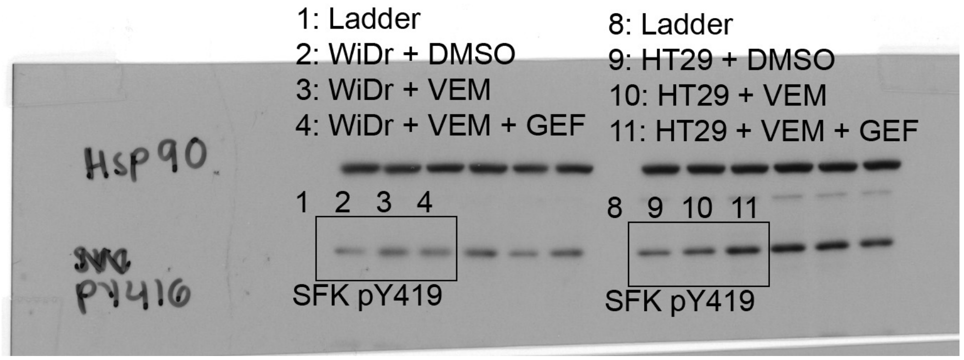

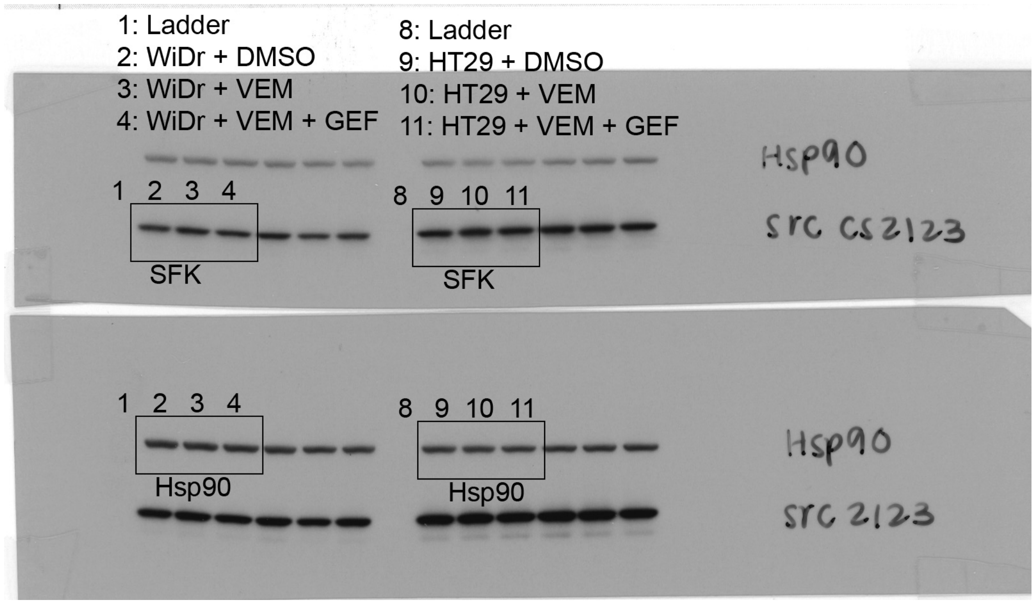

Figure 3a – western blot part 1

KM20 panels

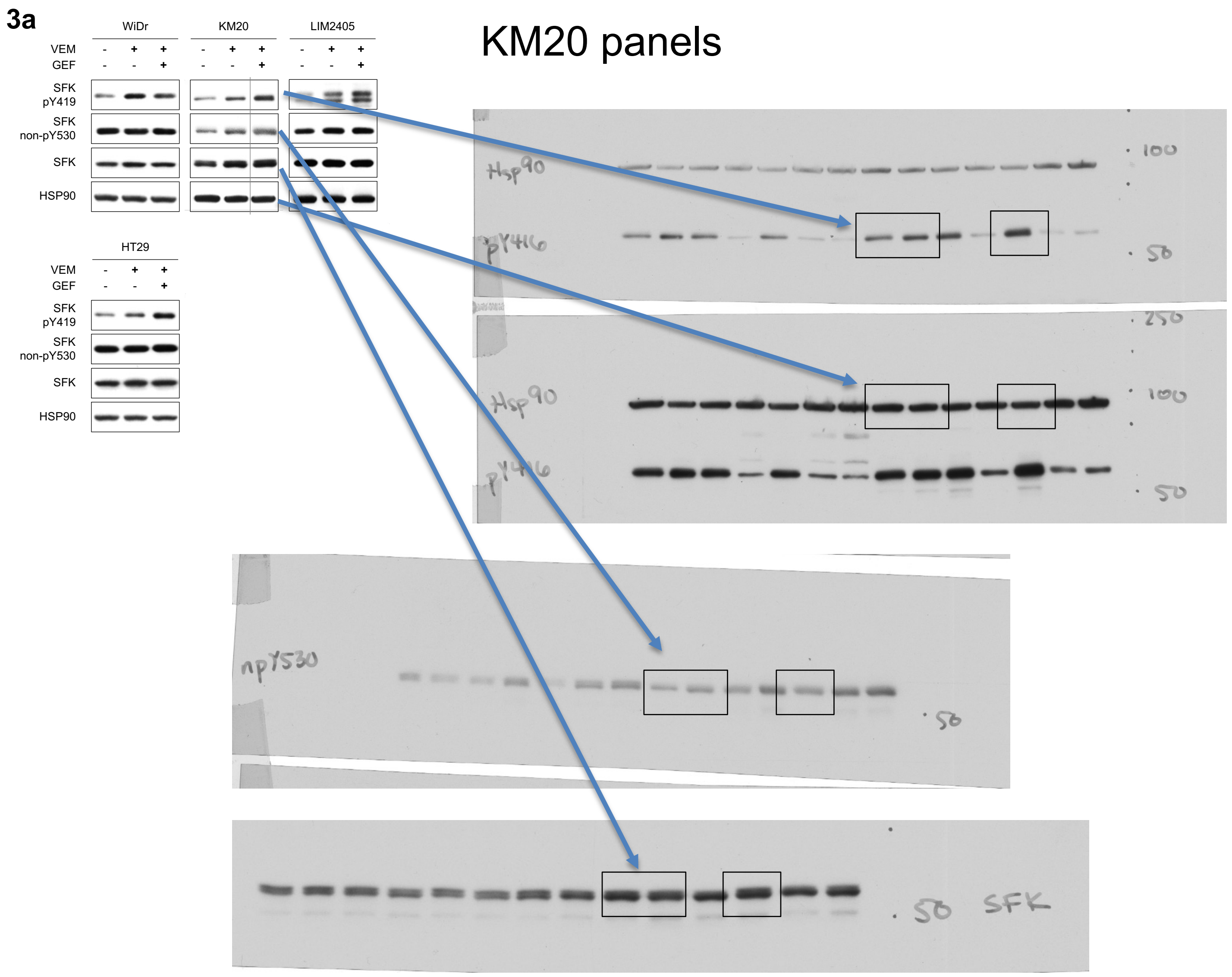

Figure 3a – western blot part 2

LIM2405 panels

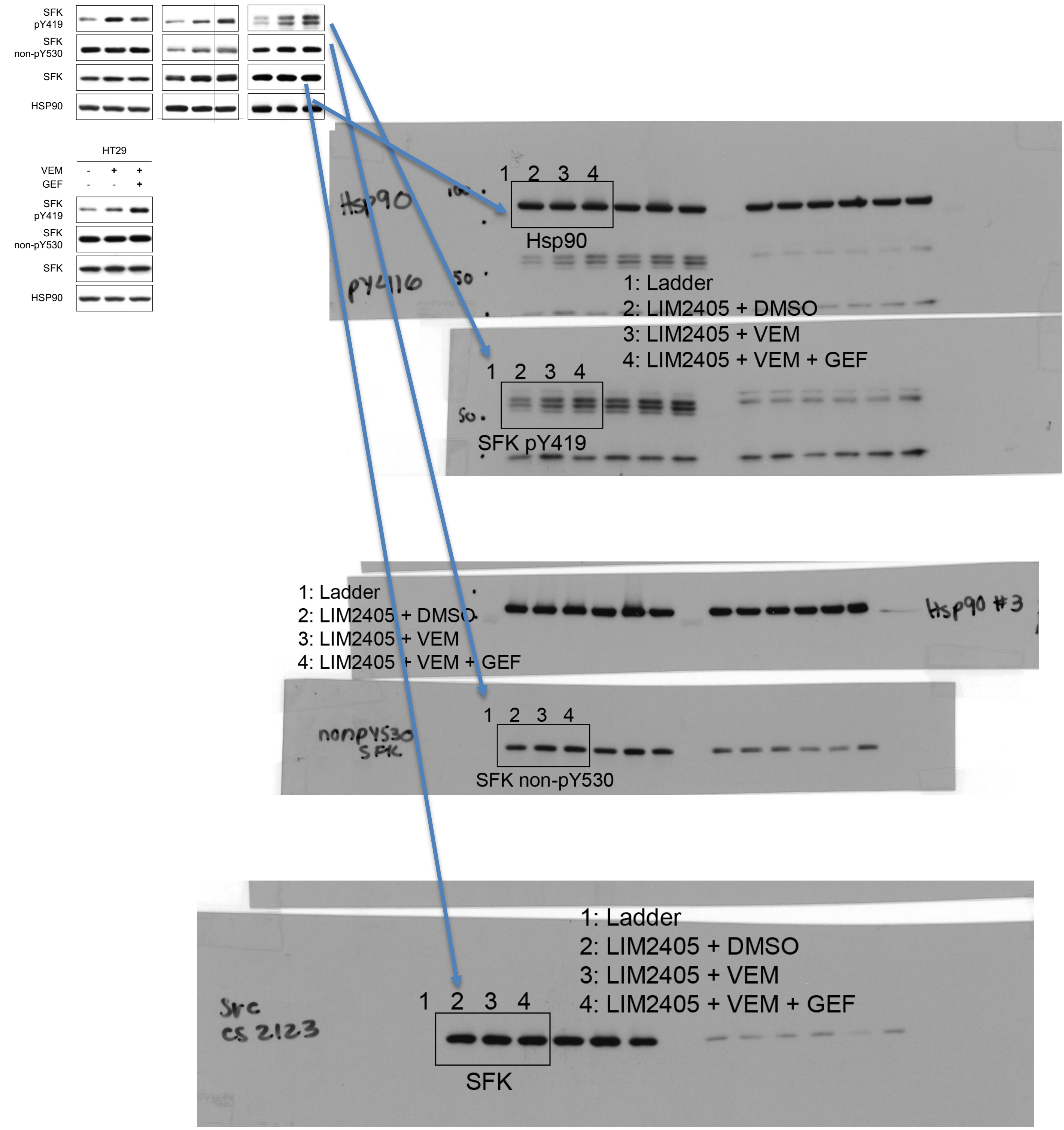

Figure 3a – western blot part 3

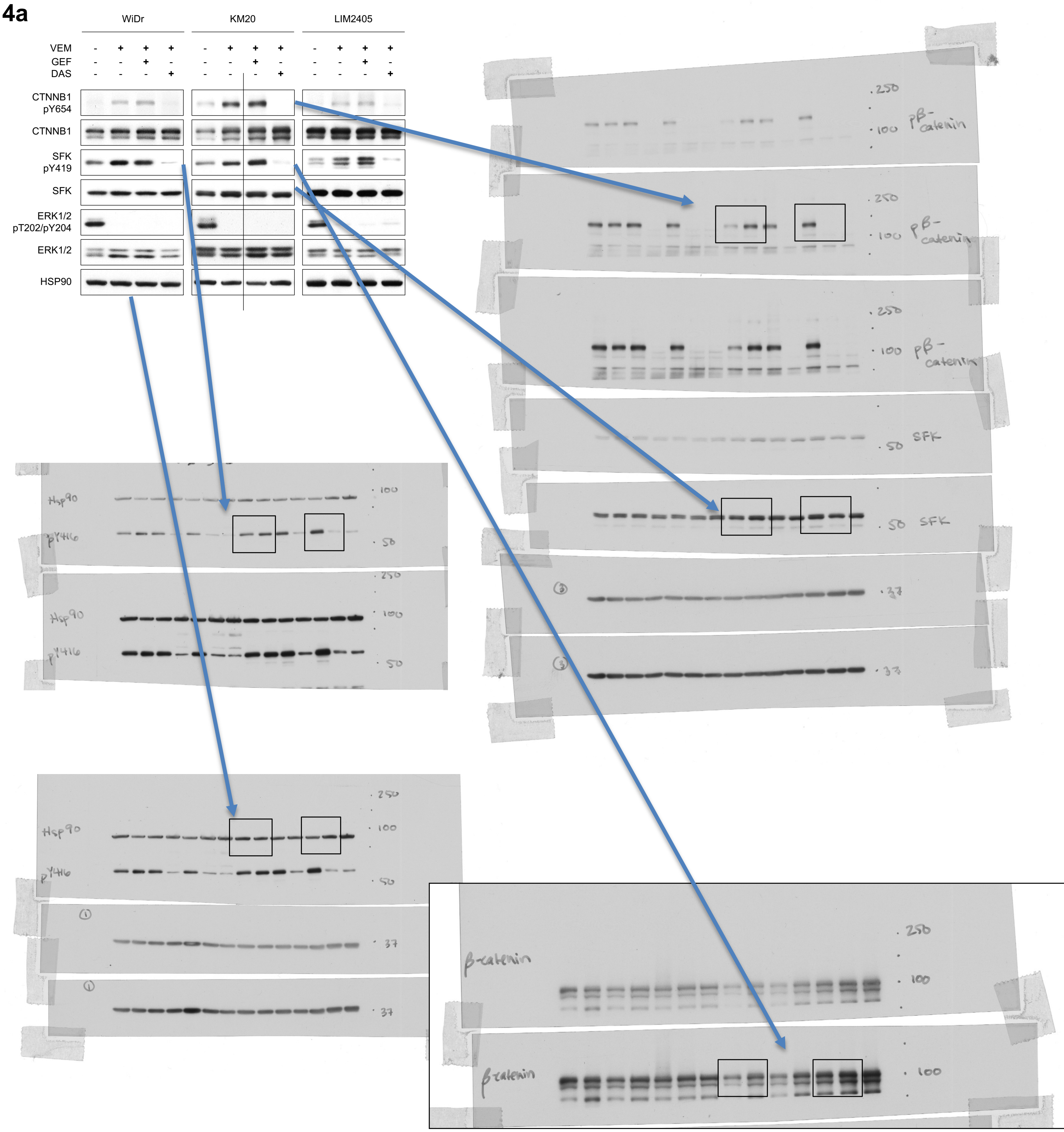

Figure 4a – western blot part 1

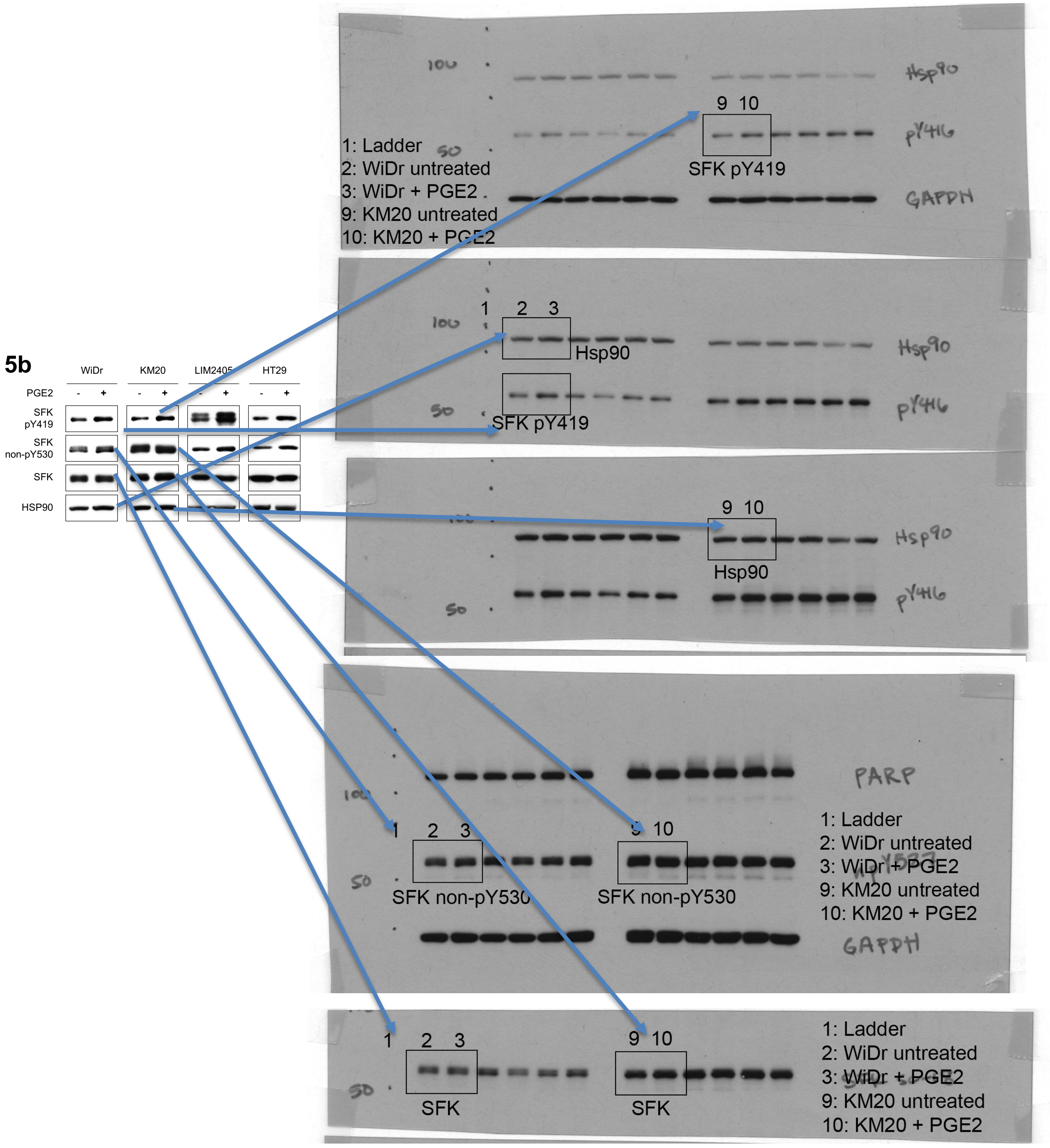

Figure 5b– western blot part 1

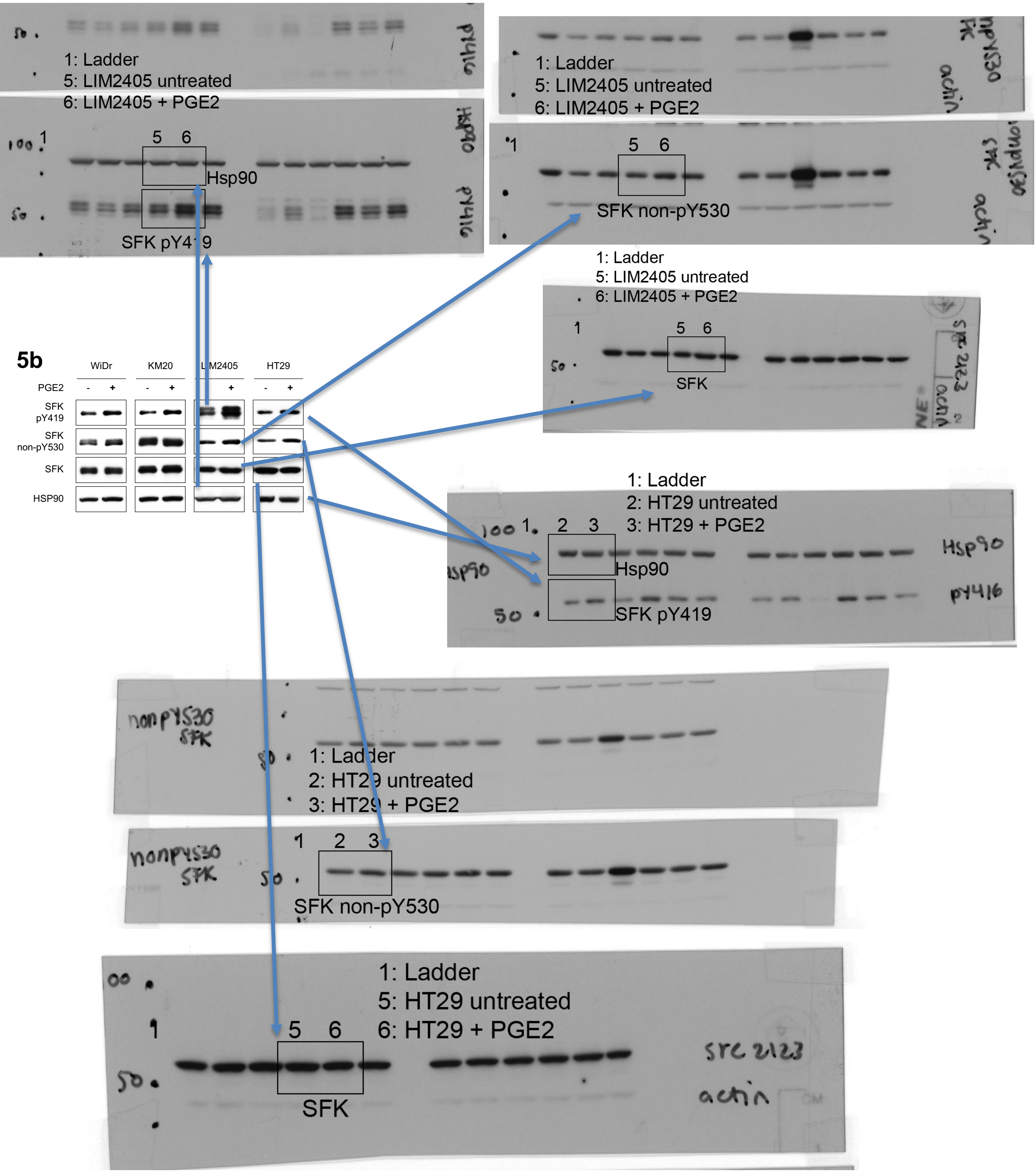

Figure 5b– western blot part 2

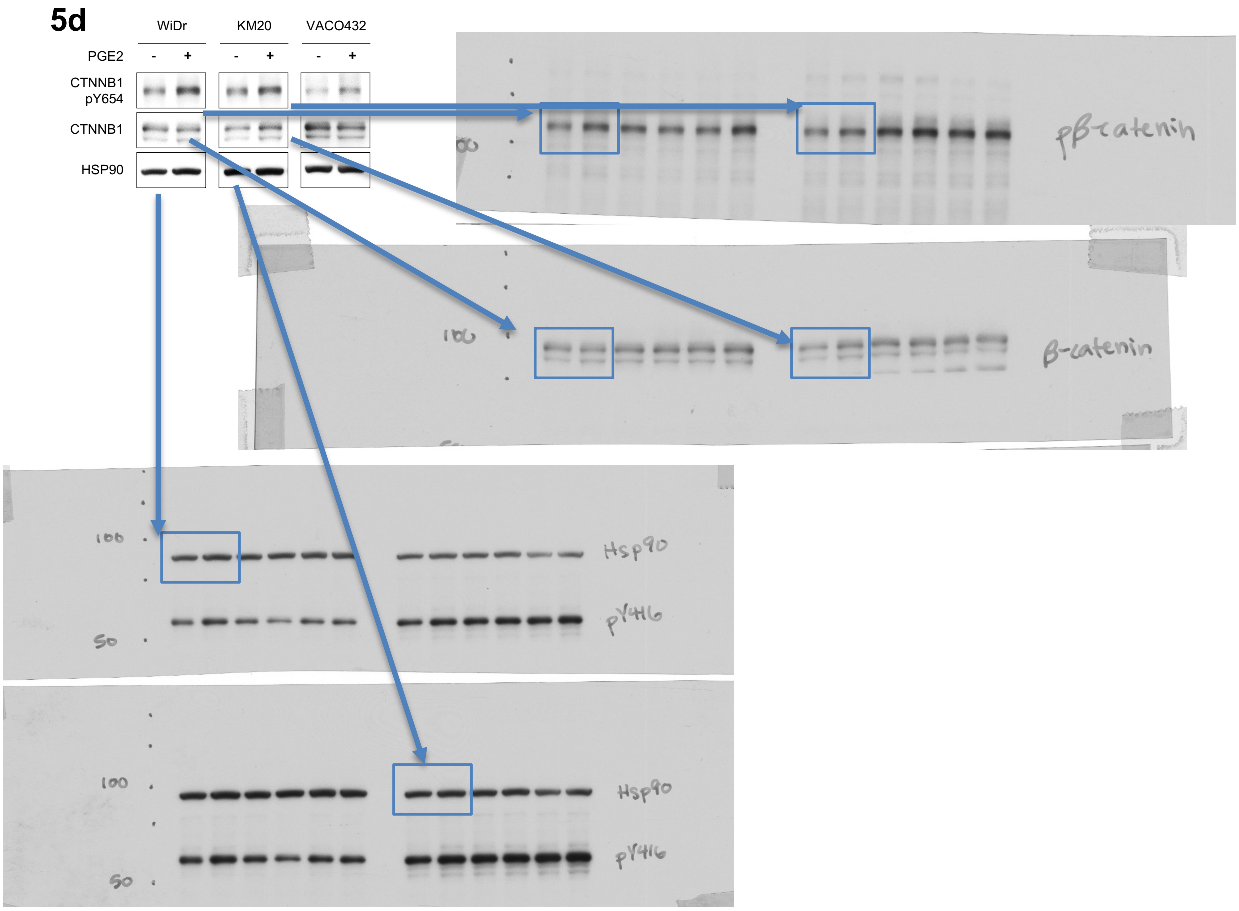

Figure 5d– western blot part 1

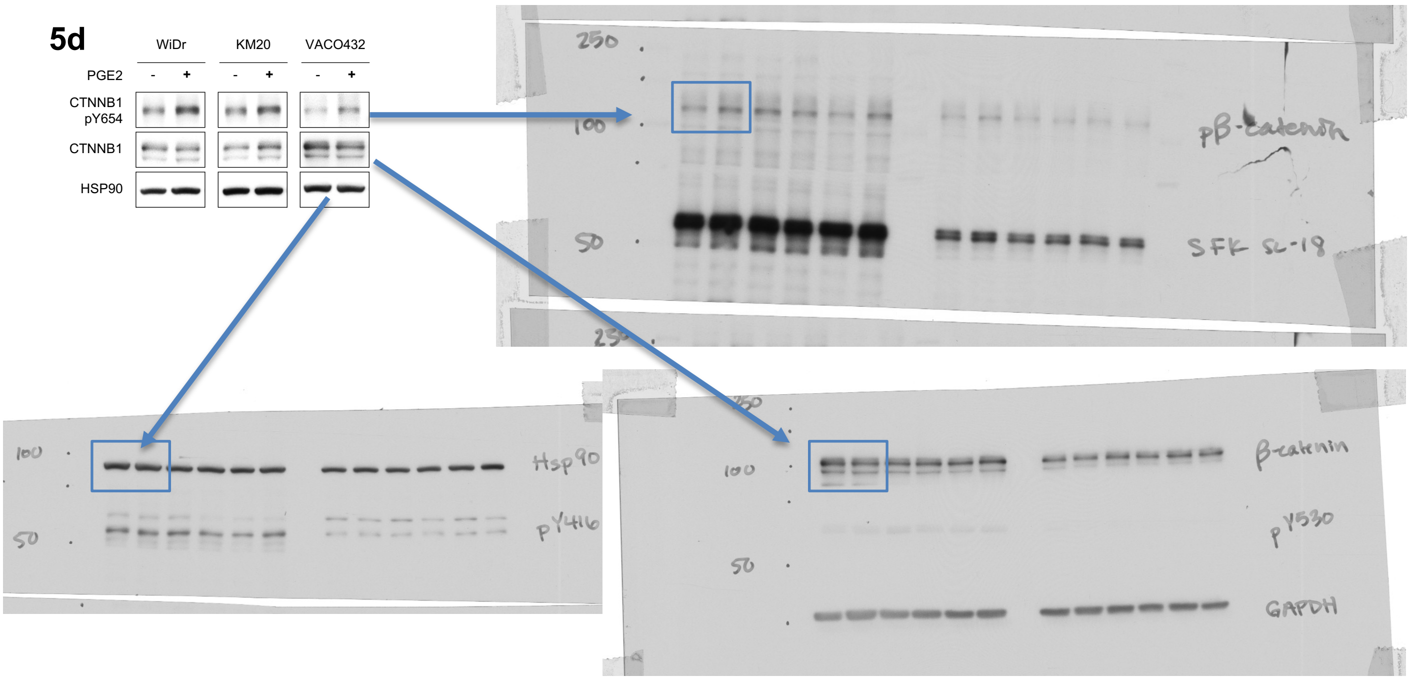

Figure 5d– western blot part 2

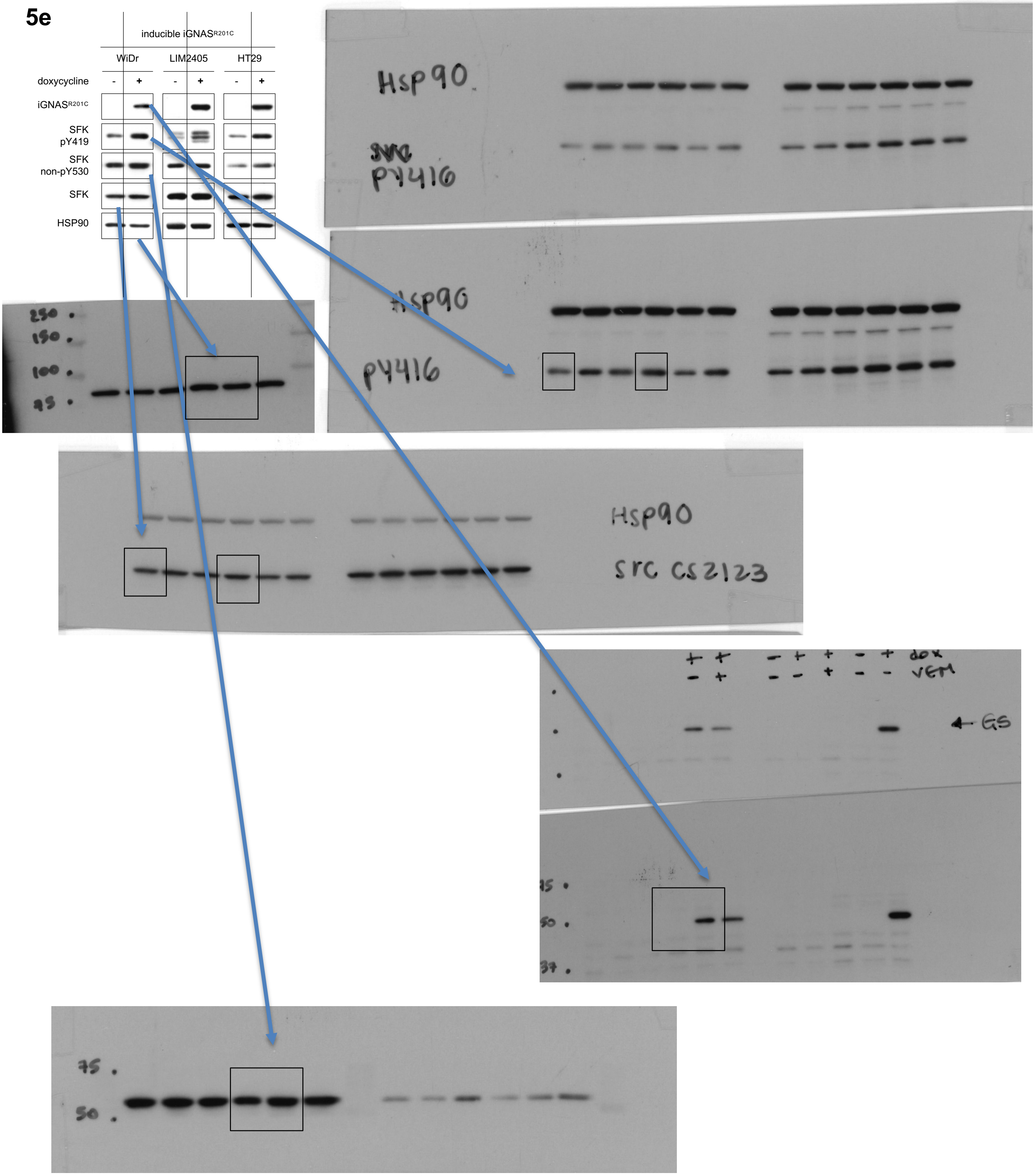

Figure 5e– western blot part 1

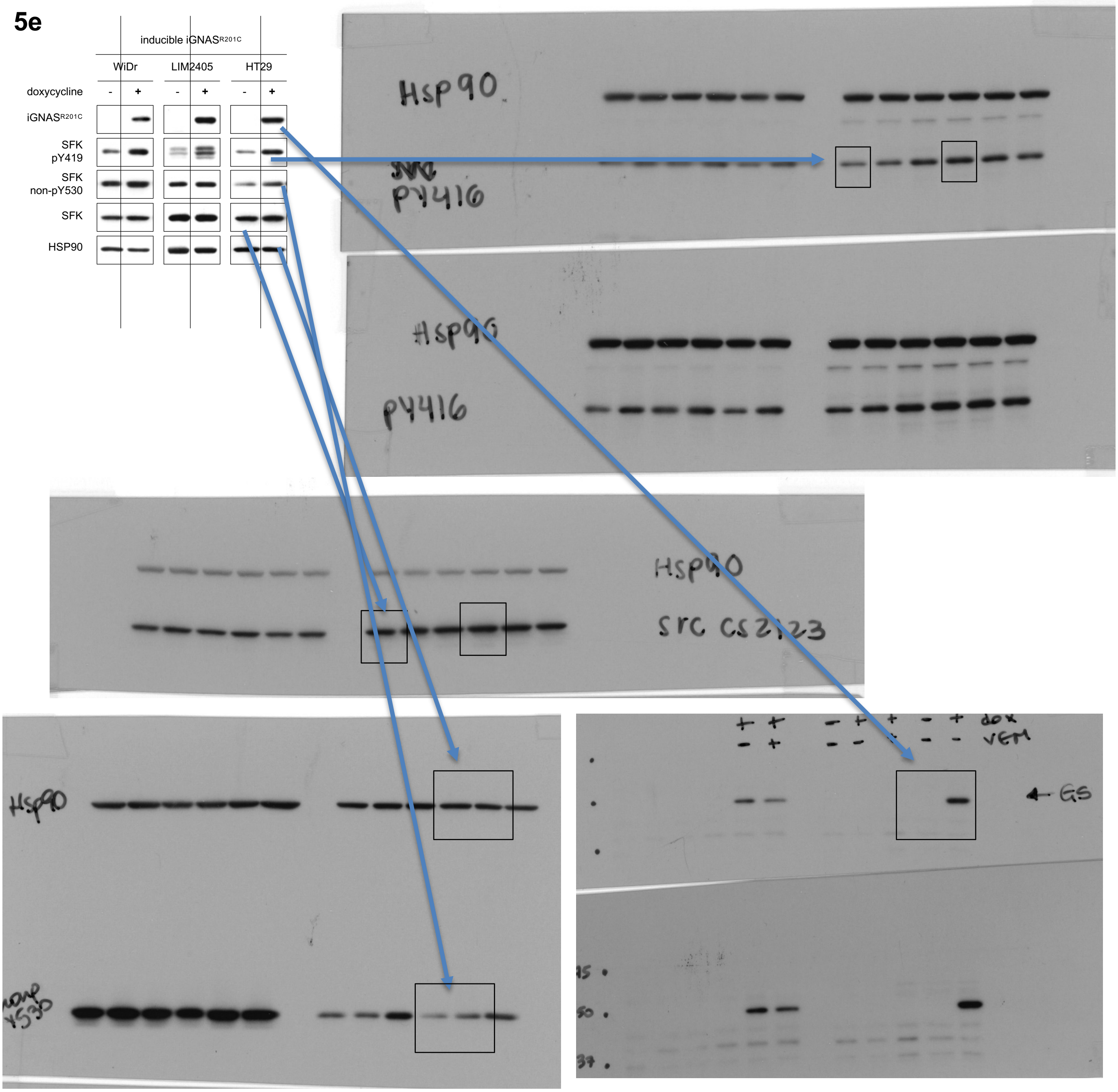

Figure 5e– western blot part 2

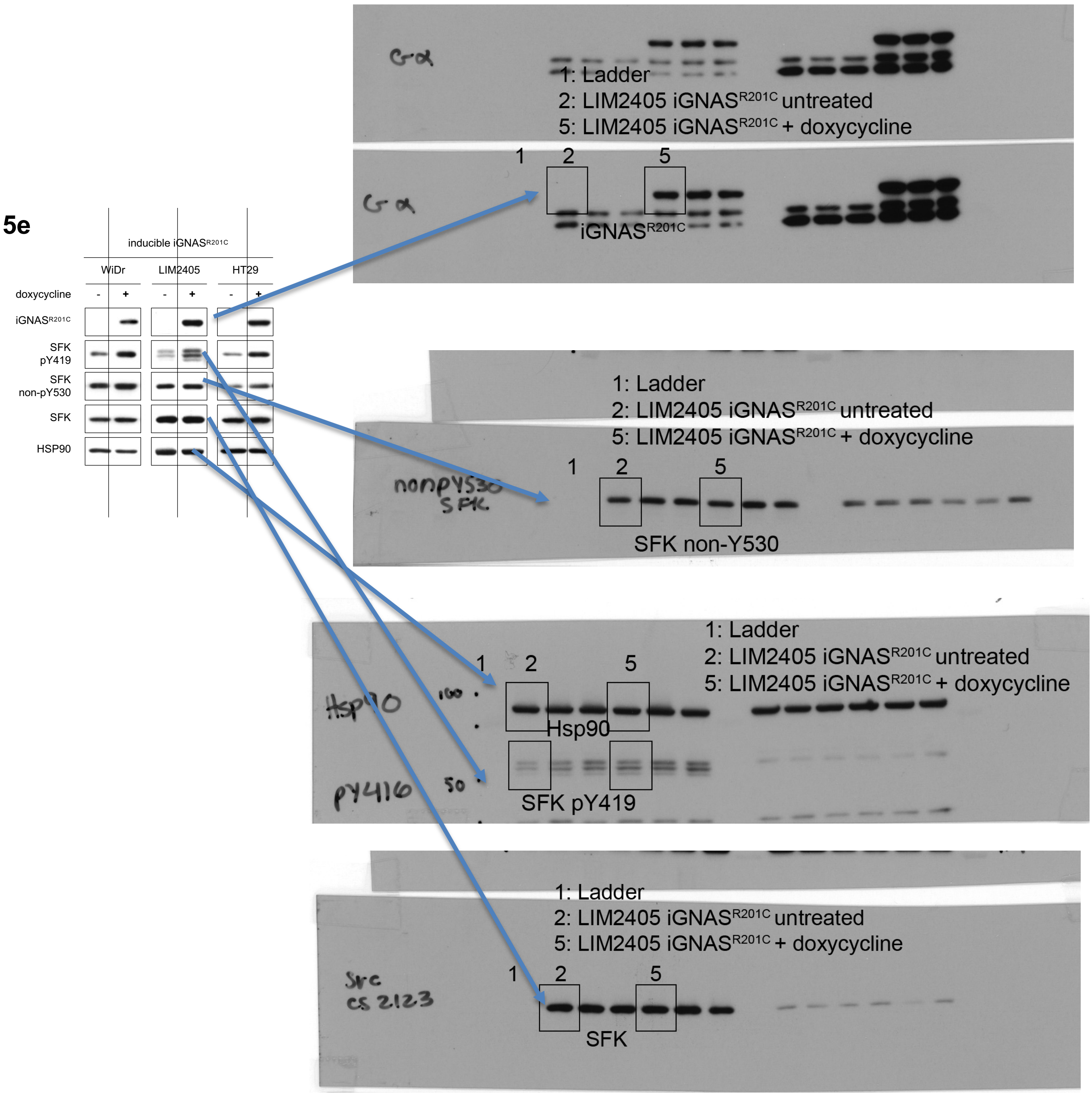

Figure 5e– western blot part 3

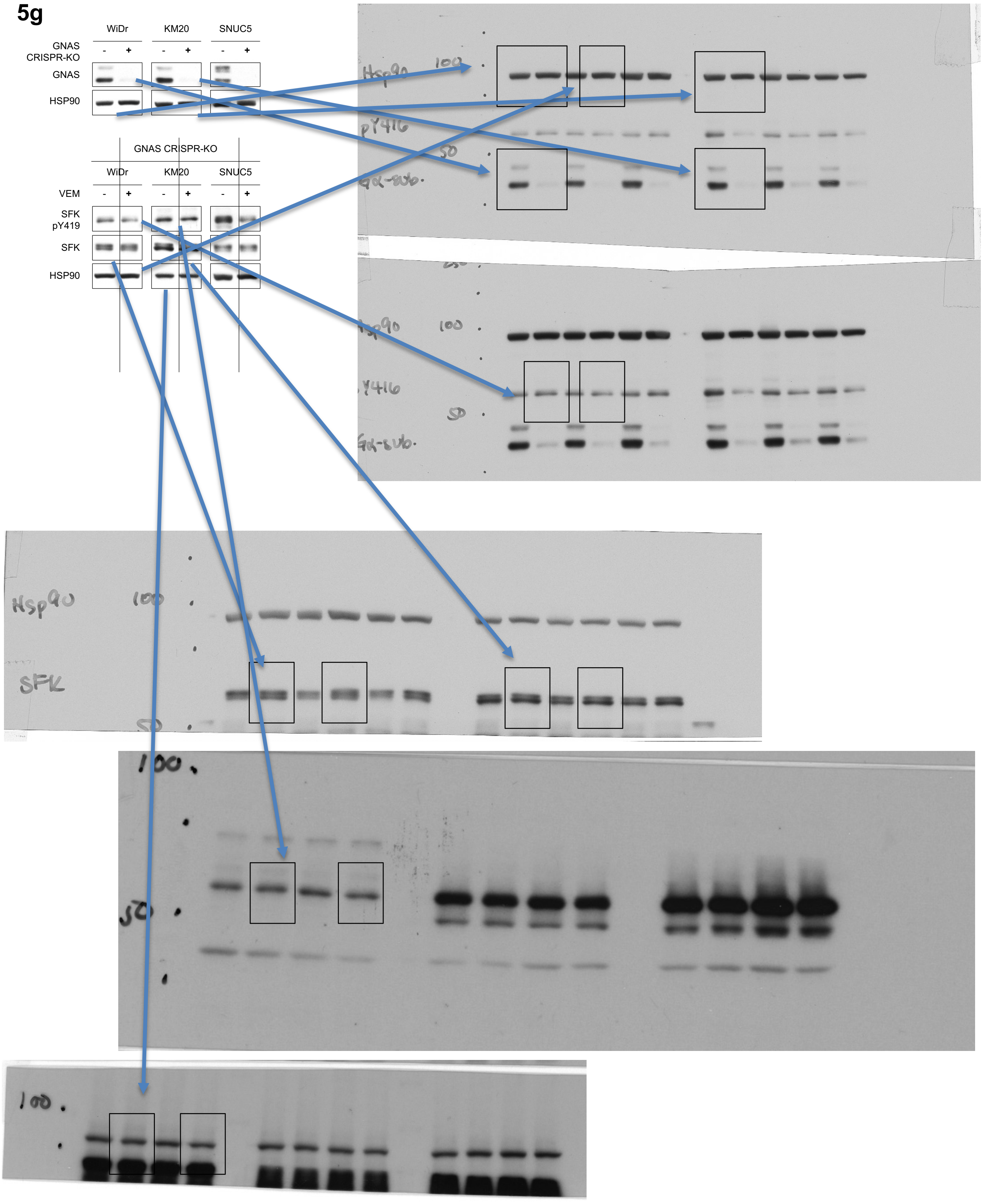

Figure 5g – western blot part 1

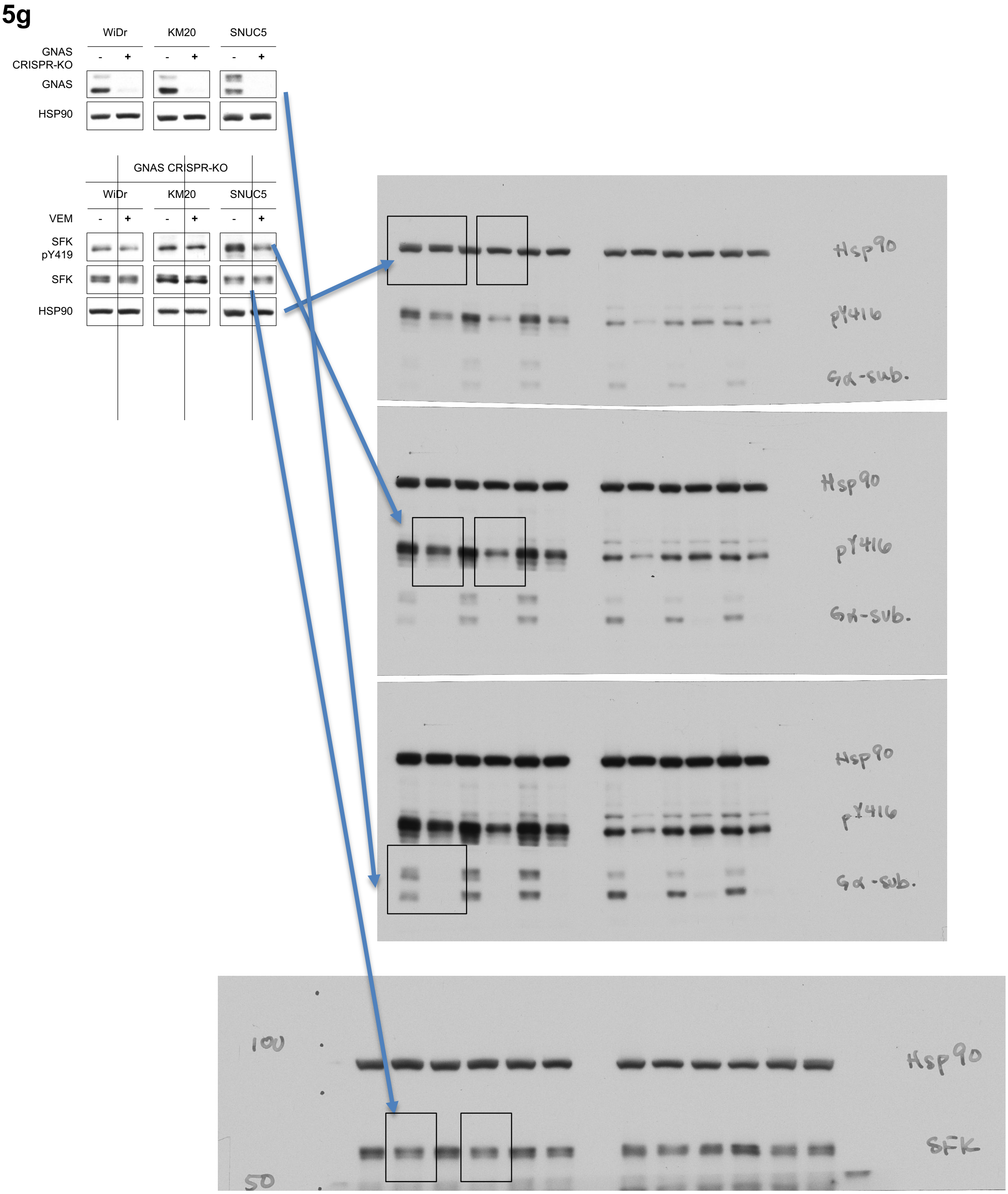

Figure 5g – western blot part 2

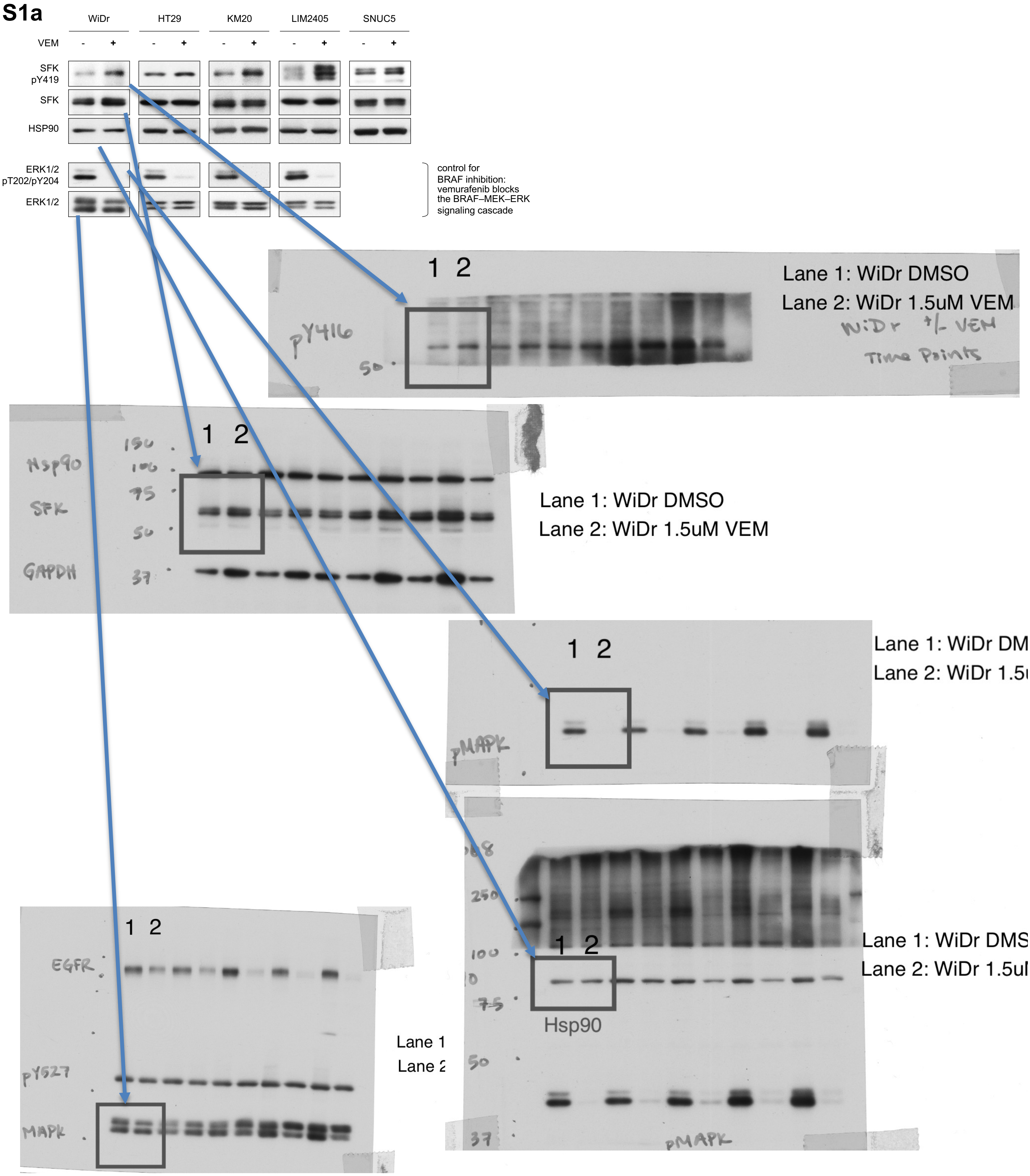

Extended Data Figure S1a – western blot part 1

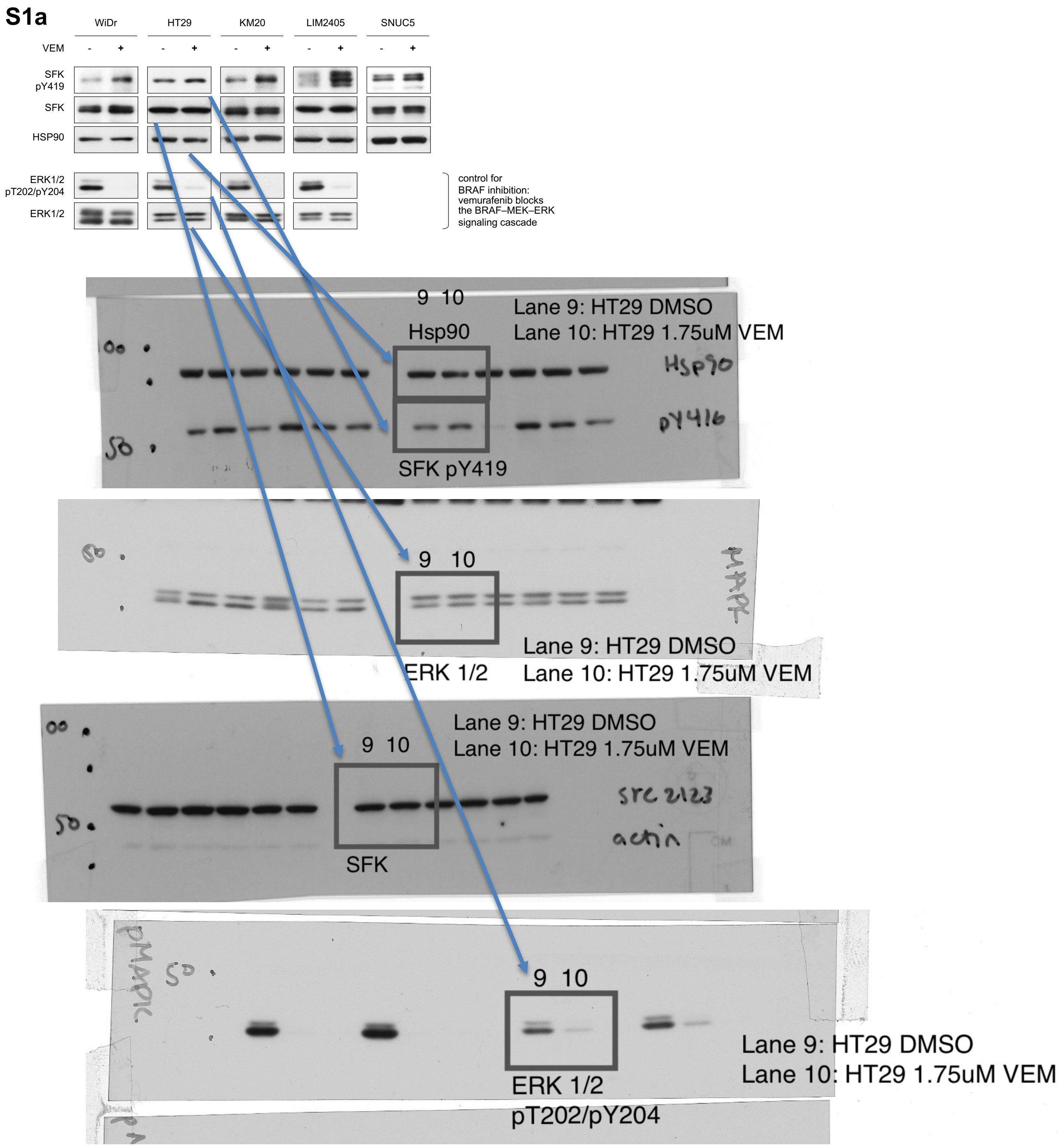

Extended Data Figure S1a – western blot part 2

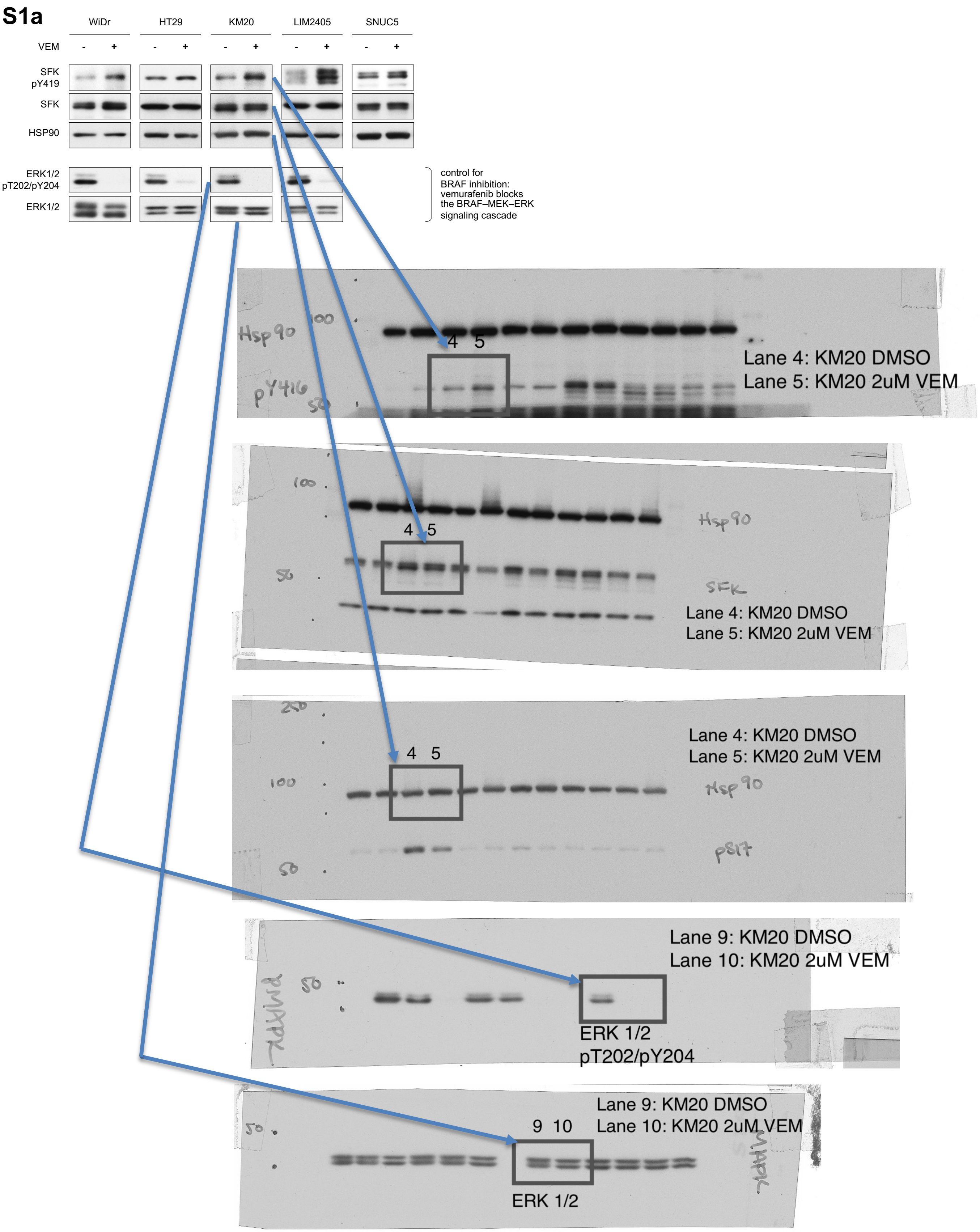

Extended Data Figure S1a – western blot part 3

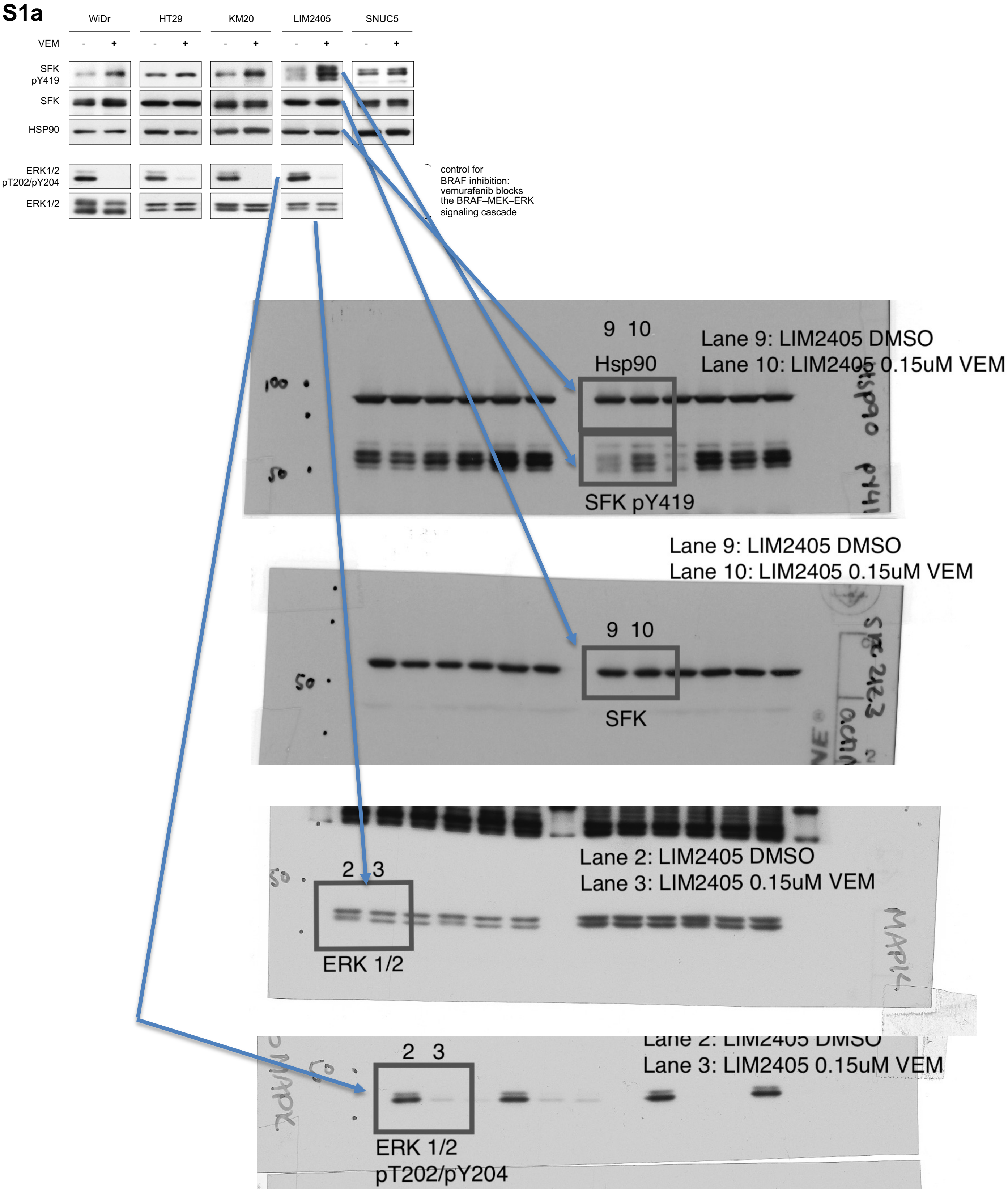

Extended Data Figure S1a – western blot part 4

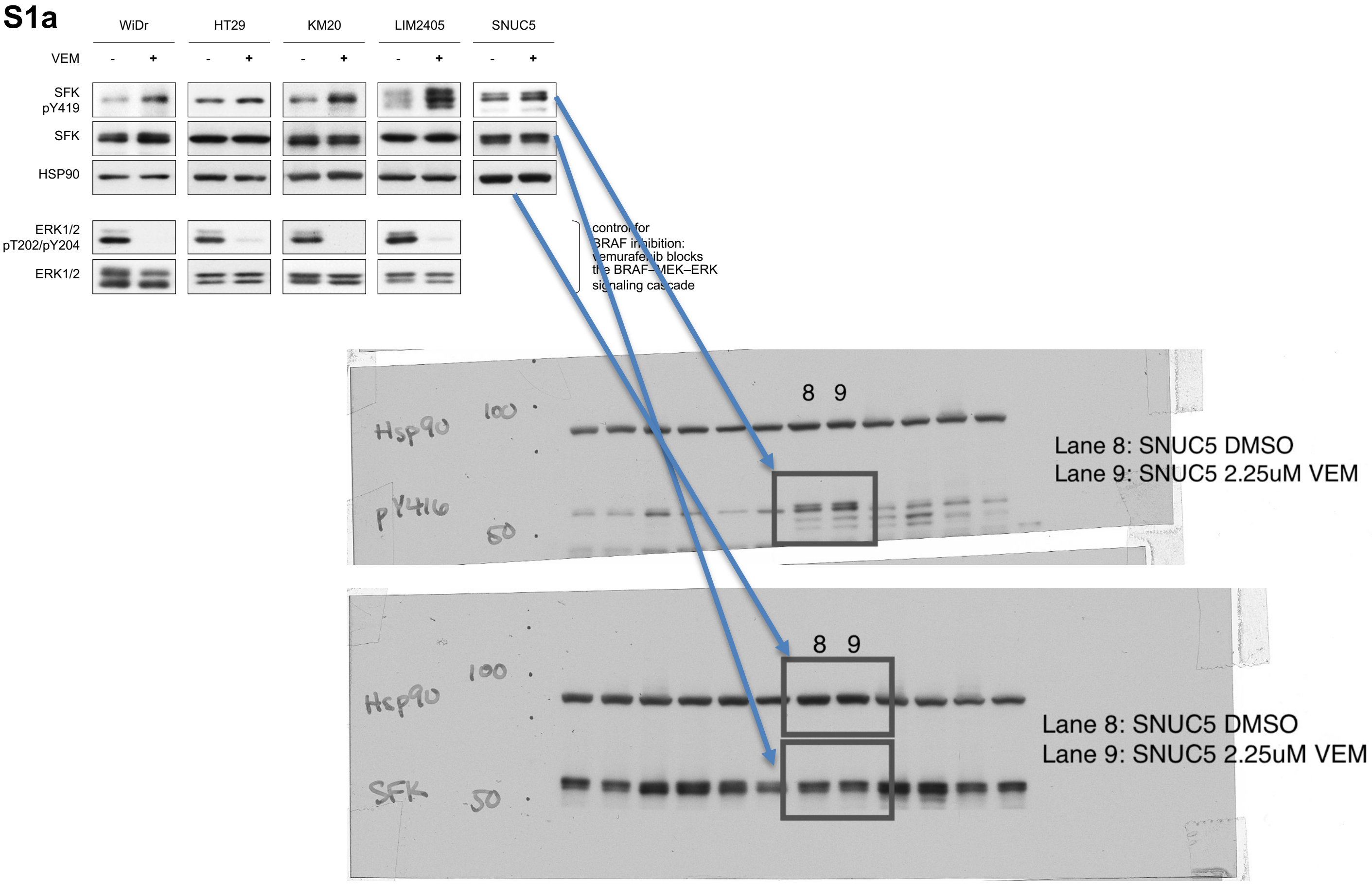

Extended Data Figure S1a – western blot part 5

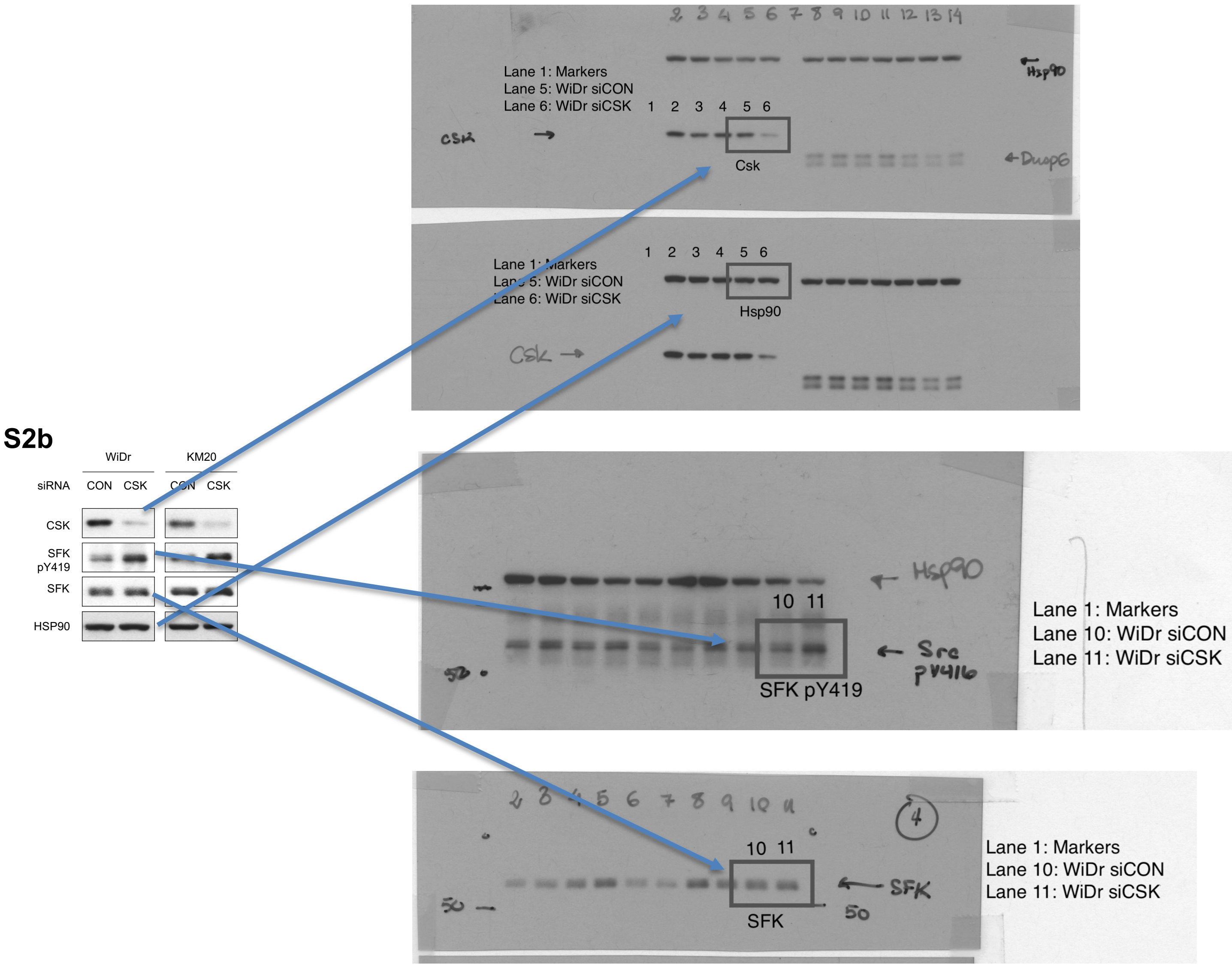

Extended Data Figure S2b – western blot part 1

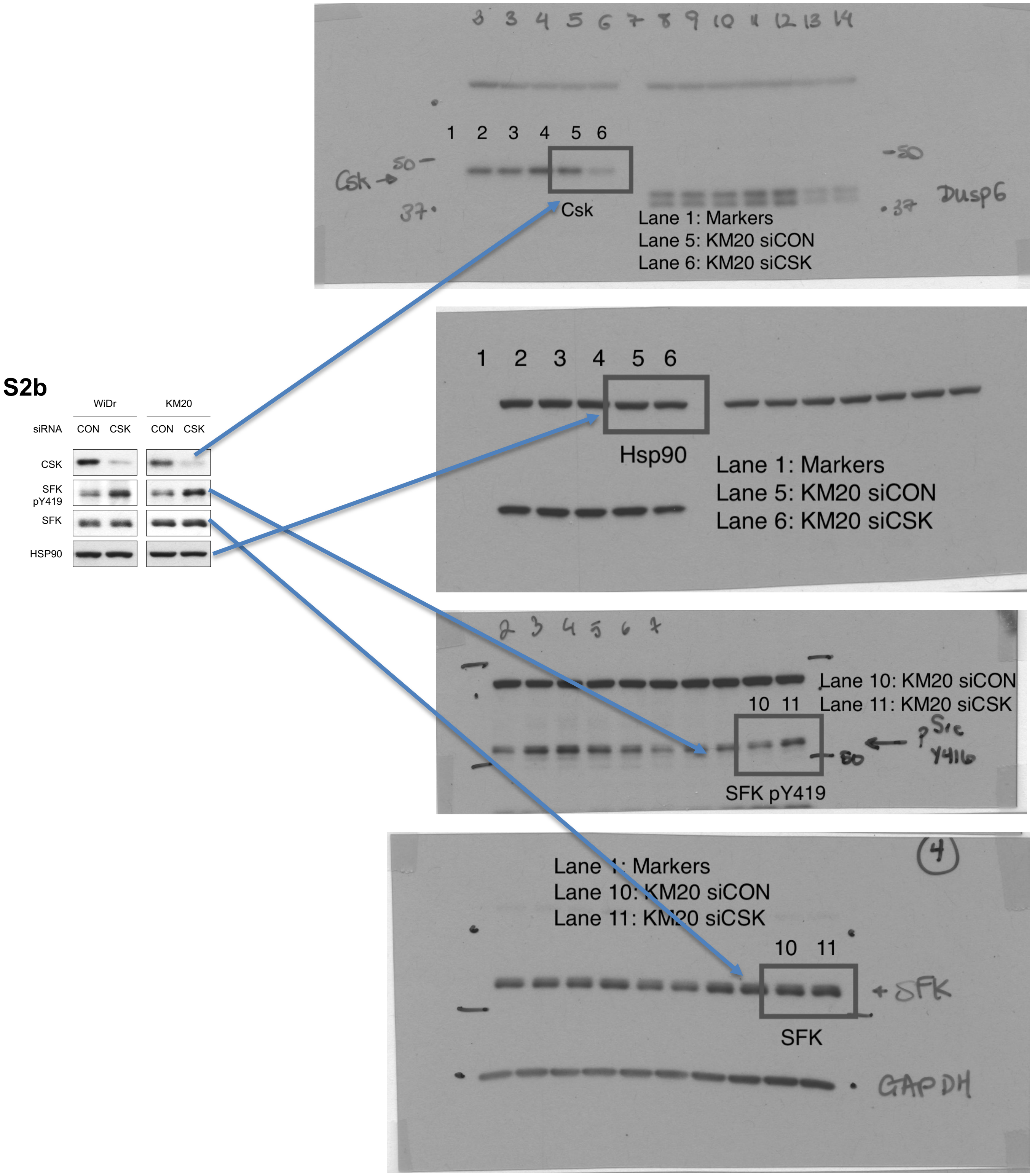

Extended Data Figure S2b – western blot part 2

S4a

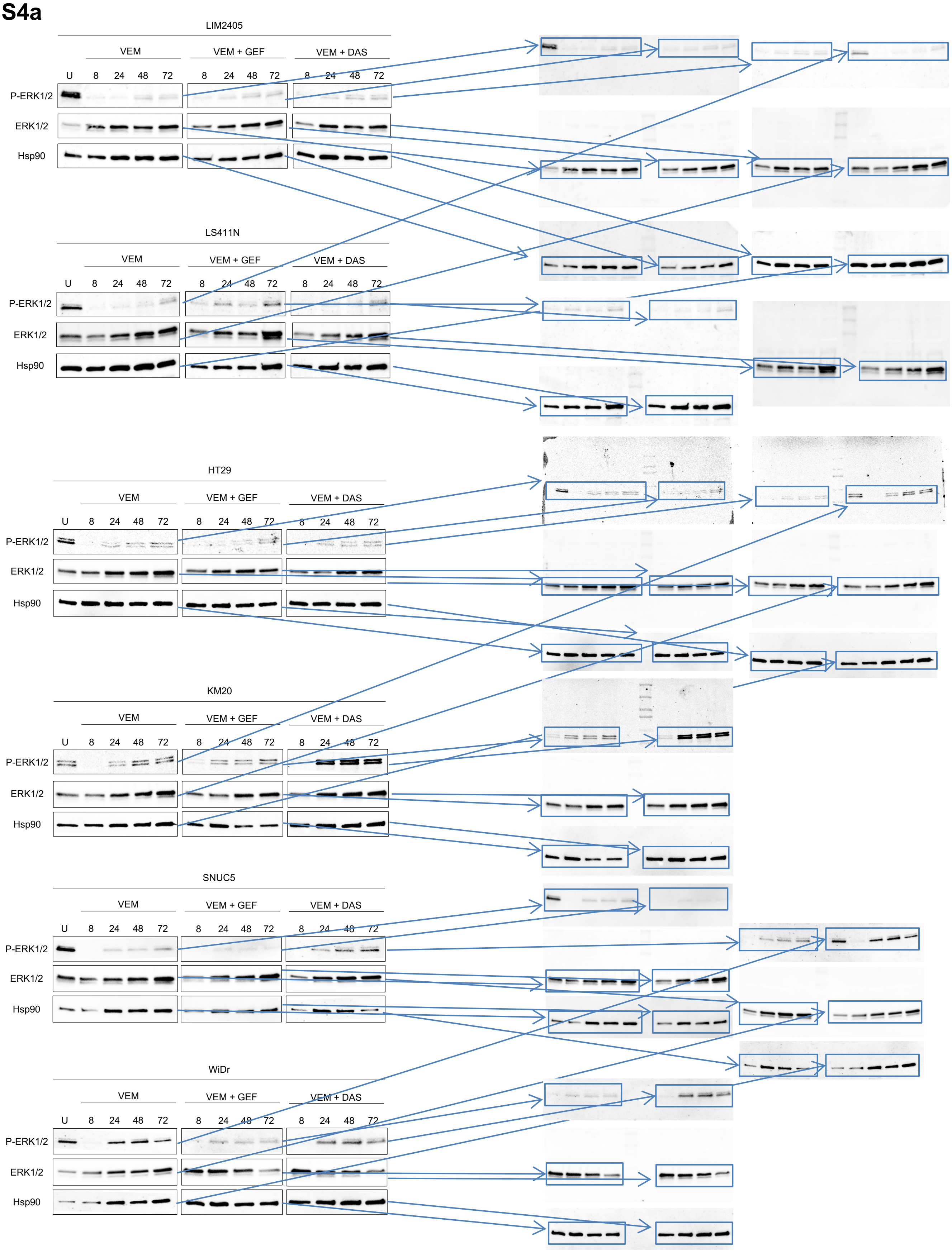

Extended Data Figure S4a – western blot part 1

S4a

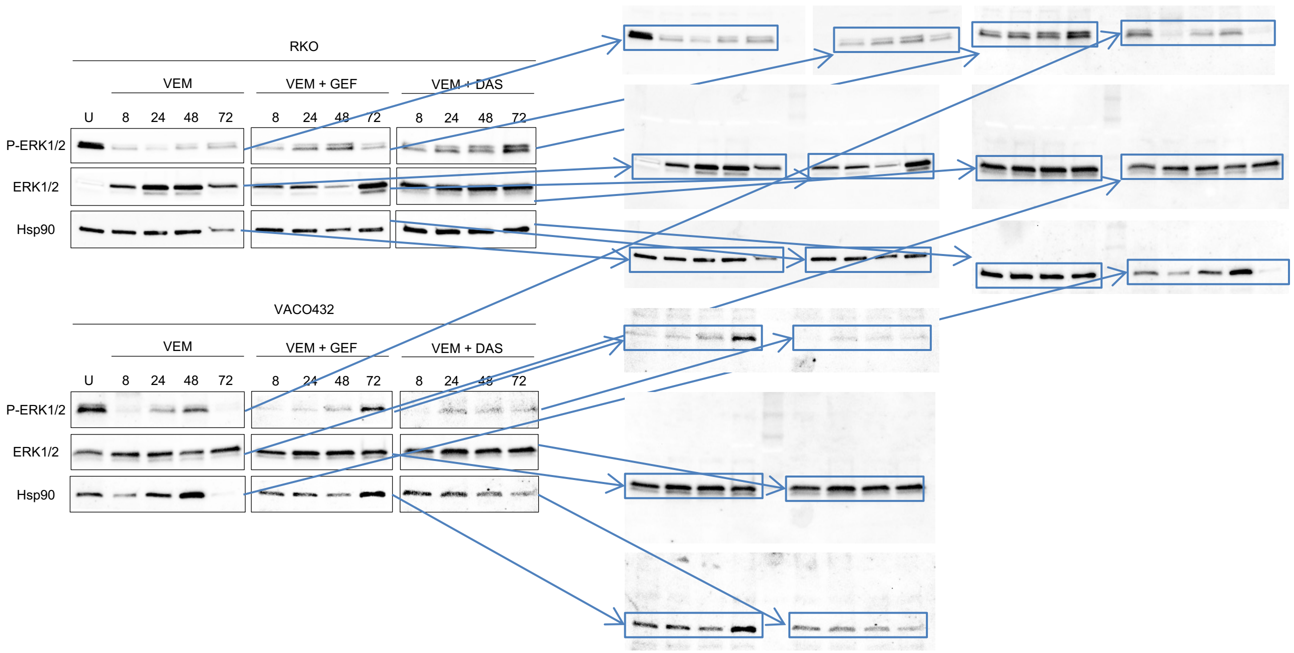

Extended Data Figure S4a – western blot part 2

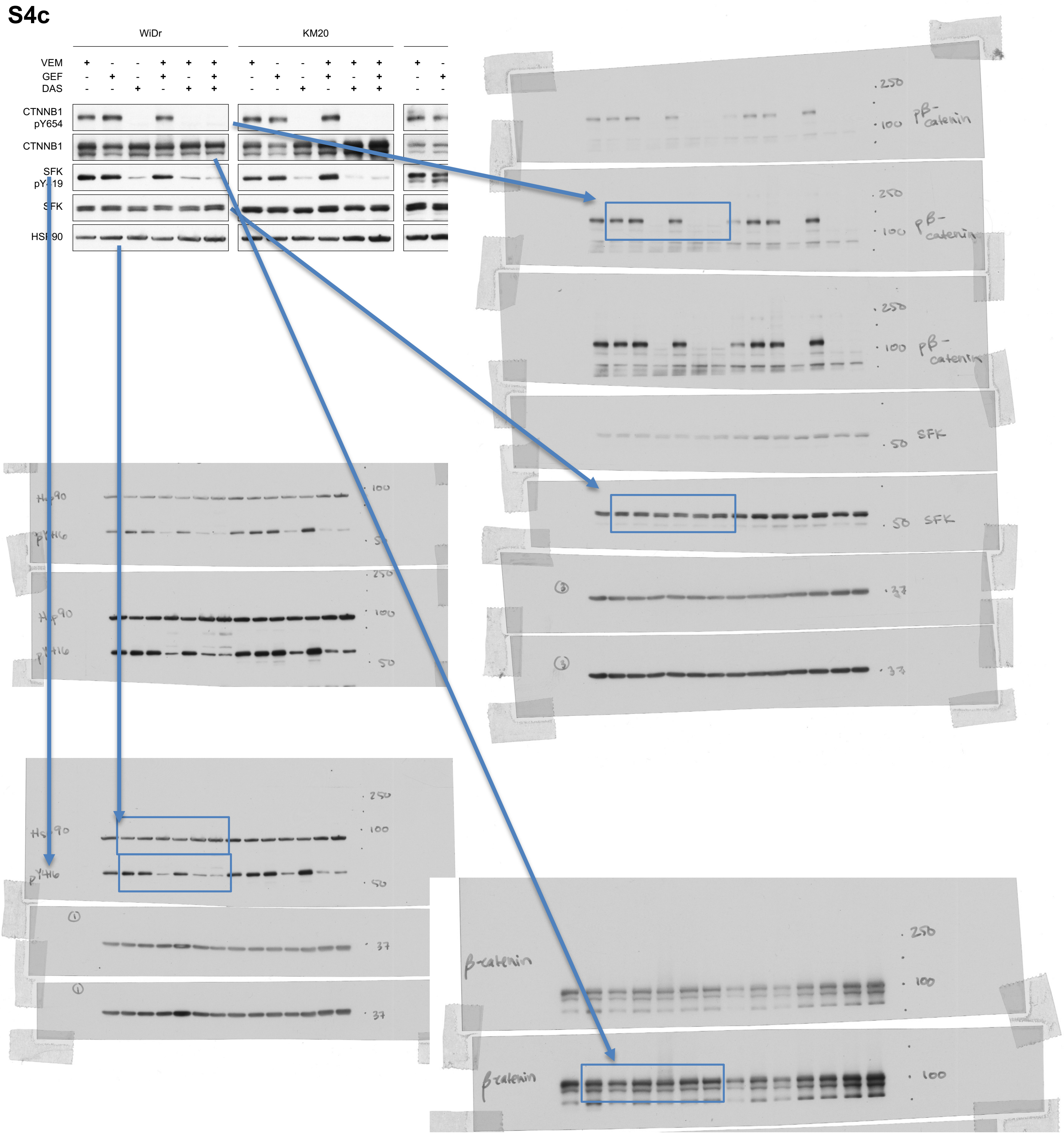

Extended Data Figure S4c – western blot part 1

S4c

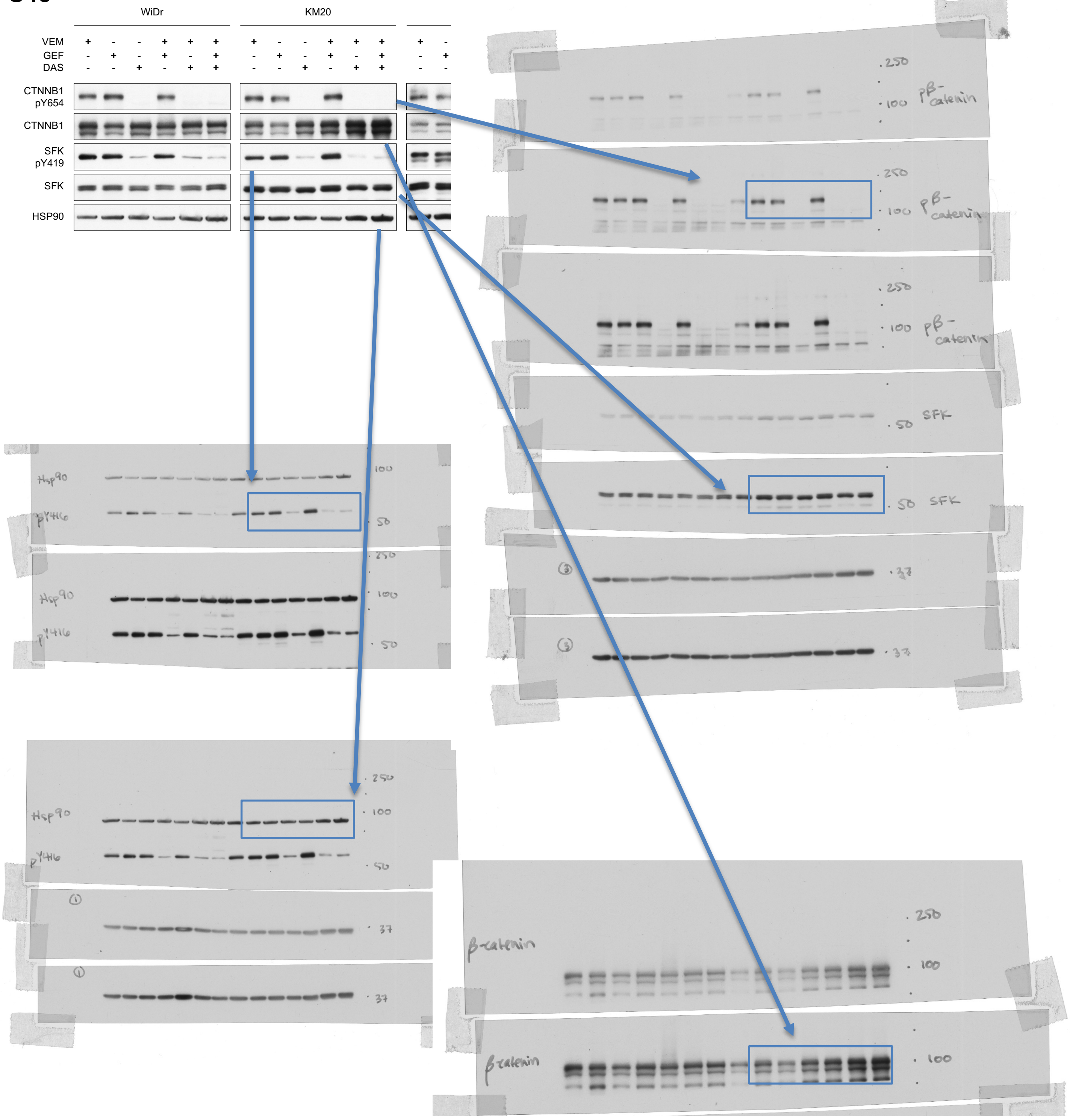

Extended Data Figure S4c – western blot part 2

S4c

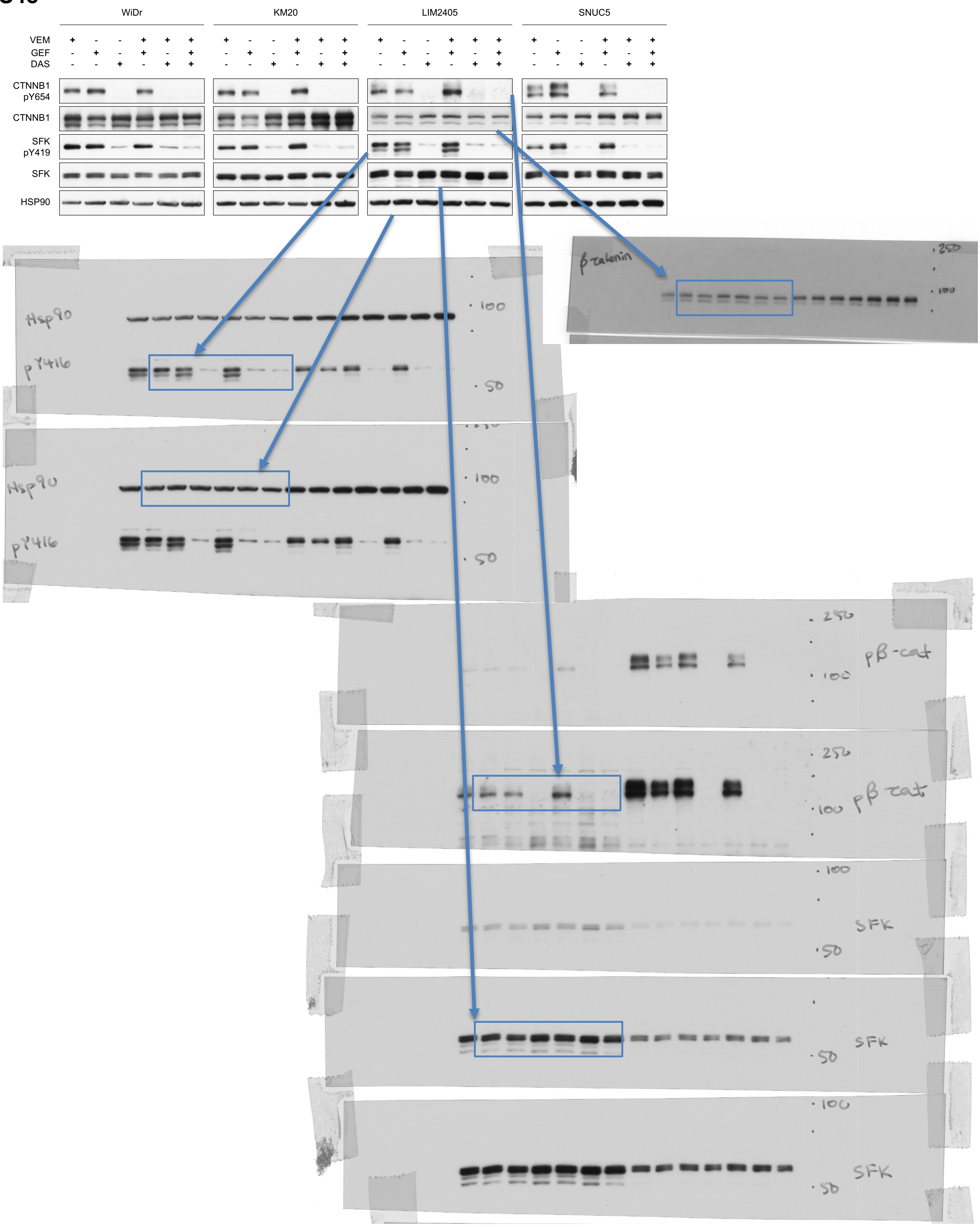

Extended Data Figure S4c – western blot part 3

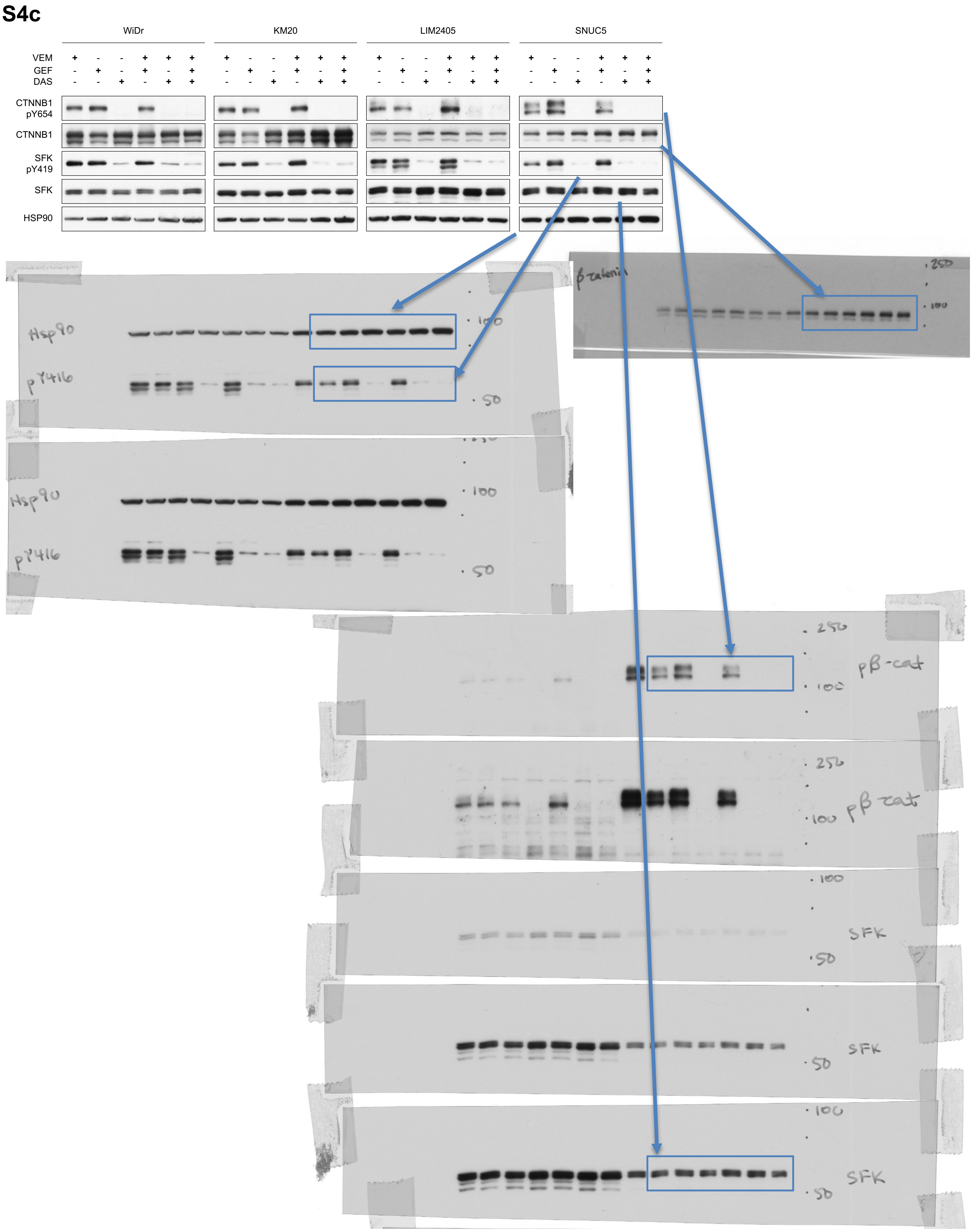

Extended Data Figure S4c – western blot part 4

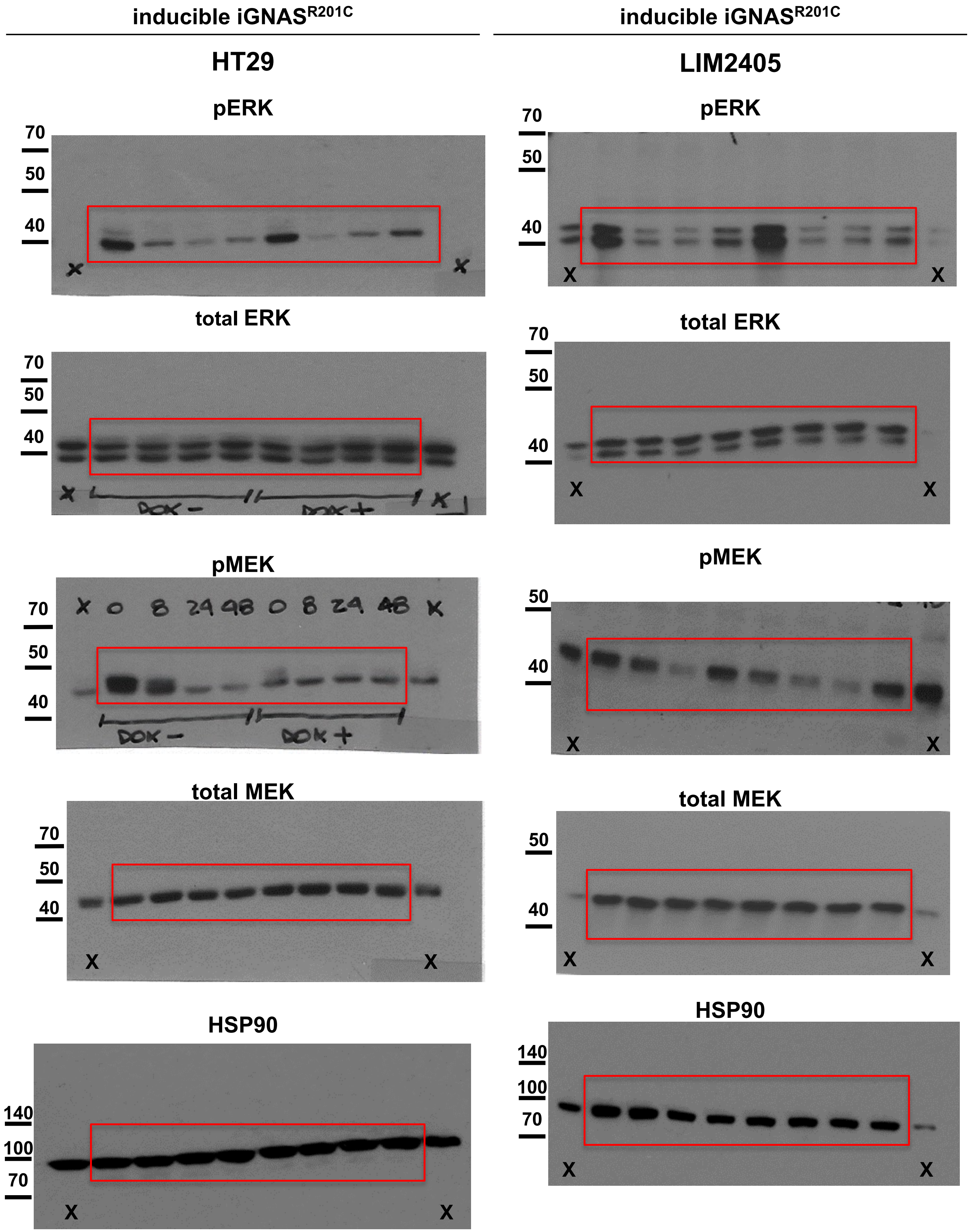

Extended Data Figure S5a-b – western blot part 1

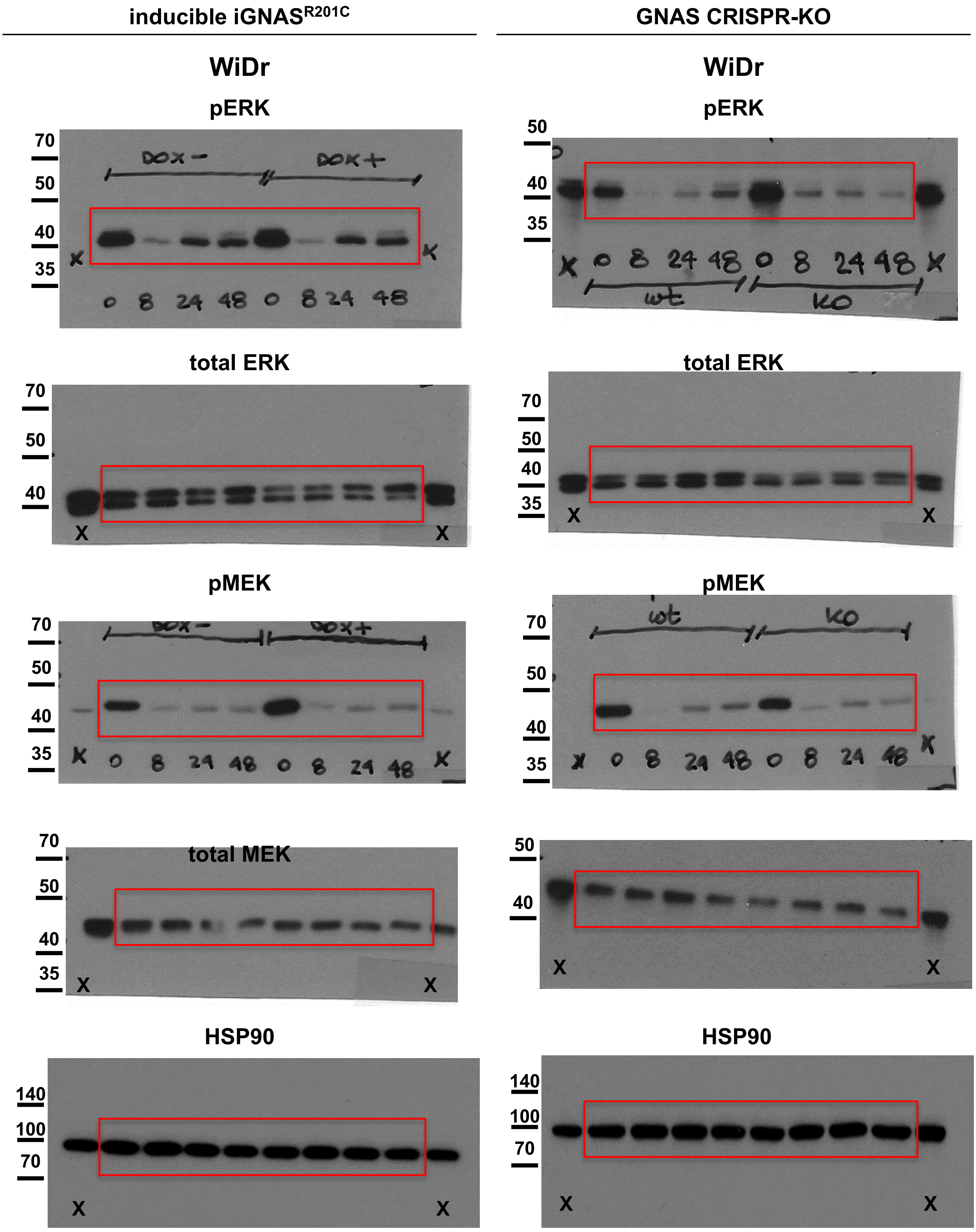

Extended Data Figure S5a-b – western blot part 2

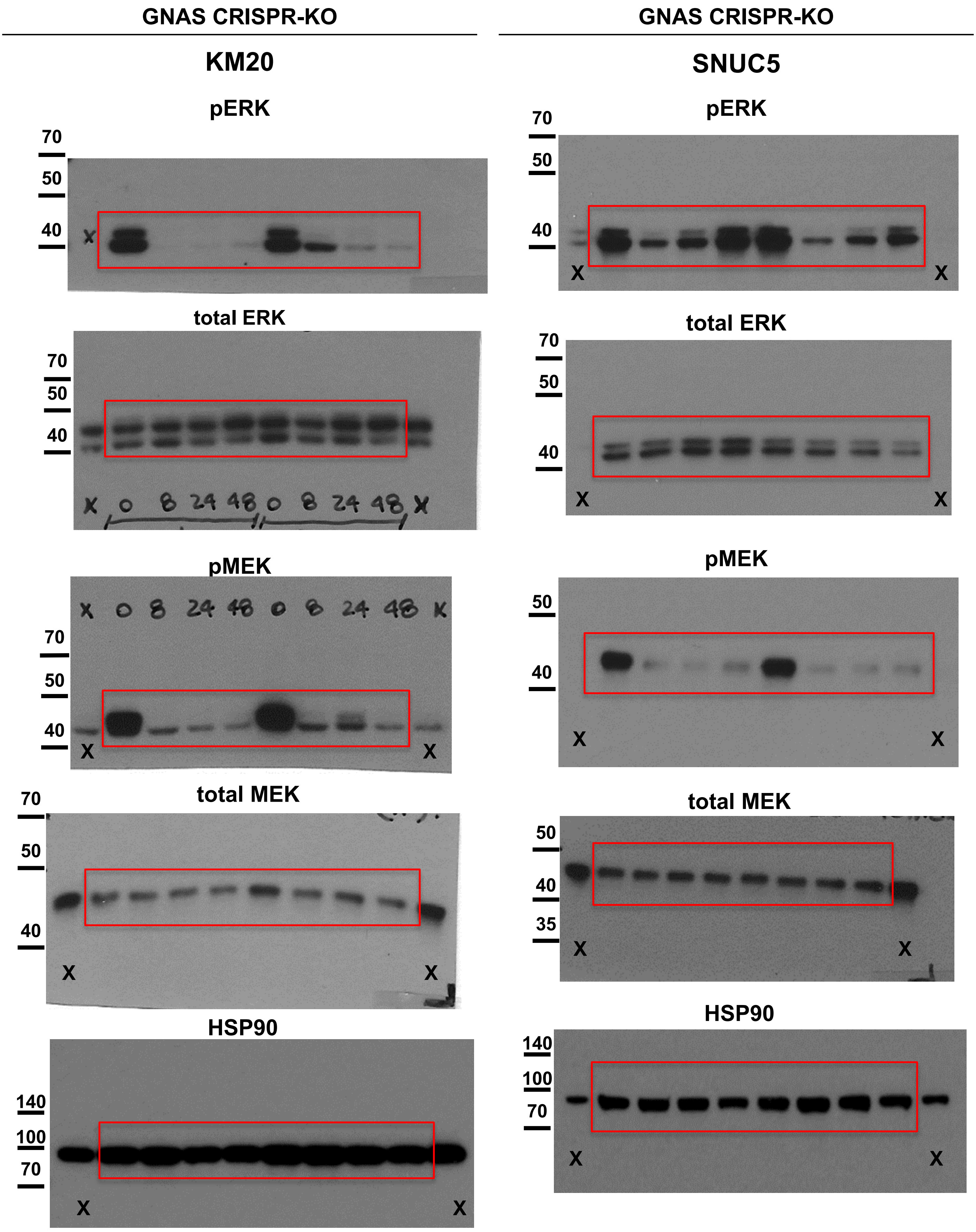

Extended Data Figure S5a-b – western blot part 3

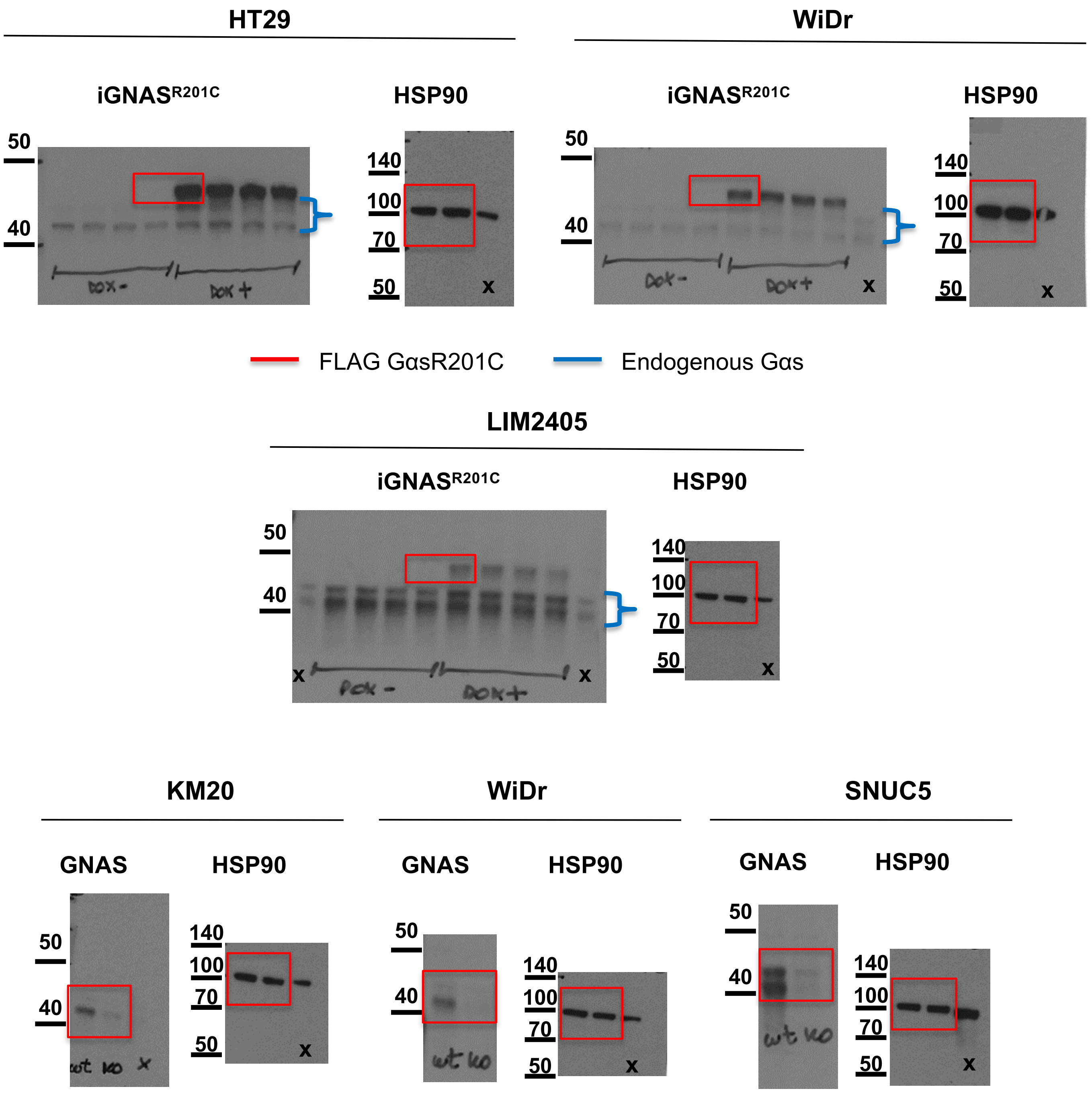

Extended Data Figure S5a-b – western blot part 4
